# Remarkable Enhancement of Catalytic Activity of Cu‐Complexes in the Electrochemical Hydrogen Evolution Reaction by Using Triply Fused Porphyrin**

**DOI:** 10.1002/cssc.202201146

**Published:** 2022-12-14

**Authors:** Shubhadeep Chandra, Arijit Singha Hazari, Qian Song, David Hunger, Nicolás. I. Neuman, Joris van Slageren, Elias Klemm, Biprajit Sarkar

**Affiliations:** ^1^ Lehrstuhl für Anorganische Koordinationschemie Institut für Anorganische Chemie Universität Stuttgart Pfaffenwaldring 55 70569 Stuttgart Germany; ^2^ Institut für Technische Chemie Universität Stuttgart Pfaffenwaldring 55 70569 Stuttgart Germany; ^3^ Institut für Physikalische Chemie Universität Stuttgart Pfaffenwaldring 55 70569 Stuttgart Germany; ^4^ Instituto de Desarrollo Tecnológico para la Industria Química CCT INTEC, UNL-CONICET Predio CONICET Santa Fe Dr. Alberto Cassano Ruta Nacional N° 168, Km 0, Paraje El Pozo S3000ZAA Santa Fe Argentina

**Keywords:** copper, electrocatalysis, fused porphyrin, proton reduction, turnover number

## Abstract

A bimetallic triply fused copper(II) porphyrin complex (**1**) was prepared, comprising two monomeric porphyrin units linked through β–β, *meso*–*meso*, β′–β′ triple covalent linkages and exhibiting remarkable catalytic activity for the electrochemical hydrogen evolution reaction in comparison to the analogous monomeric copper(II) porphyrin complex (**2**). Electrochemical investigations in the presence of a proton source (trifluoroacetic acid) confirmed that the catalytic activity of the fused metalloporphyrin occurred at a significantly lower overpotential (≈320 mV) compared to the non‐fused monomer. Controlled potential electrolysis combined with kinetic analysis of catalysts **1** and **2** confirmed production of hydrogen, with 96 and 71 % faradaic efficiencies and turnover numbers of 102 and 18, respectively, with an observed rate constant of around 10^7^ s^−1^ for the dicopper complex. The results thus firmly establish triply fused porphyrin ligands as outstanding candidates for generating highly stable and efficient molecular electrocatalysts in combination with earth‐abundant 3d transition metals.

## Graphical abstract

**Triple threat**: Bimetallic triply fused copper containing metalloporphyrin (**1**) displays excellent activity towards electrochemical proton reduction. The activity is far better compared to its monomeric counterpart (**2**).

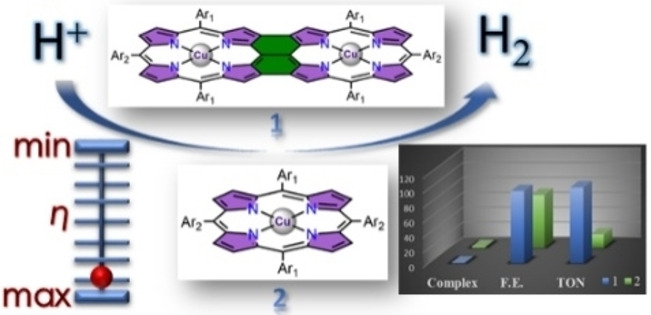

## Introduction

Rapid depletion of fossil fuels, limited resources, and growing energy demands have led to a global search for alternative energy sources.^[1]^ Hydrogen as an energy carrier is considered to be one of the promising alternatives as it is carbon‐free and only generates the environmentally benign by‐product H_2_O upon combustion.^[2]^ Electrocatalytic reduction of protons is one of the reliable strategies for sustainable production of hydrogen.^[3]^ In the recent past, the electrocatalytic hydrogen evolution reaction (HER) (2H^+^+e^−^→H_2_) has garnered considerable attention as a new paradigm for energy storage, delivery, and transport.^[4]^ Currently, elemental platinum is considered to be one of the most efficient catalysts for HER; however, low natural abundance and high cost limit its large‐scale applications, prompting researchers to seek alternative non‐noble metal‐based catalysts.^[5]^ Consequently, substantial effort and time have been devoted towards finding cheap, efficient, and robust catalysts comprised of Earth‐abundant elements.^[6]^ As a result, several Earth‐abundant transition metals like Fe,^[7]^ Co,^[8]^ Mo,^[9]^ Cu,^[10]^ and Ni^[11]^ have been recognized as active molecular electrocatalysts for HER.

In nature, hydrogenase enzymes containing [Fe−Fe] or [Fe−Ni] active sites catalyze reversible interconversion of protons into hydrogen with low overpotential and high turnover, suggesting the importance of noble‐metal‐free bimetallic catalysts.^[12]^ However, difficulties in isolation and utilization of these enzymes under non‐natural environments prompted researchers to design different structural and functional models mimicking the active site of hydrogenase.^[13]^ On the other hand, several functional analogues based on bimetallic electrocatalysts inspired by natural enzymes have also been designed, and in some cases they were found to exhibit superior catalytic efficiency as compared to their monomeric counterparts.^[14]^ For example, a recent study by Apfel and co‐workers demonstrated superior catalytic efficiency of a bimetallic macrocycle featuring two cofacially linked Ni^II^‐porphyrins, using a linker molecule.^[15]^

In this context, transition metal complexes of tetrapyrrolic macrocycles such as porphyrins are important due to their unique electrochemical and photophysical properties and the unprecedented reactivity in energy‐relevant small molecule activation.^[16]^ Over the years, several Fe, Ni, Co, Mo, and Cu complexes of porphyrins have gained substantial attention as HER catalysts.[^17^, ^18^] The advantage of porphyrins over other molecular catalysts lies in the ease with which the substitution pattern at the periphery controls proton transfer ability, substrate accessibility, and selectivity in product formation.[^19^, ^20^] For example, hanging carboxylic groups at the backbone of Ni^II^ hangman porphyrins were shown to facilitate HER mediated by proton‐coupled electron transfer.^[19]^

Copper, due to its rich redox chemistry and prominent role in various redox processes in biological systems, has been shown to exhibit catalytic activity in CO_2_ reduction or water oxidation reactions.^[21]^ However, the use of Cu‐based materials in HER is comparatively rare, with a handful of examples reported from Cao and co‐workers and Gross and co‐workers.^[22]^ Limited use of Cu‐containing complexes for HER is due to the enhanced propensity of Cu‐based molecular catalyst to undergo demetallation under reducing conditions, forming Cu‐nanoparticles or depositing on electrodes, all of which can catalyze HER in their own right. Therefore, the contemporary challenges in developing molecular electrocatalysts for HER is to develop systems that will operate at low overpotentials with high turnover frequencies and numbers, remaining stable under electrocatalytic conditions, while generating high current densities.

**Figure 1:**
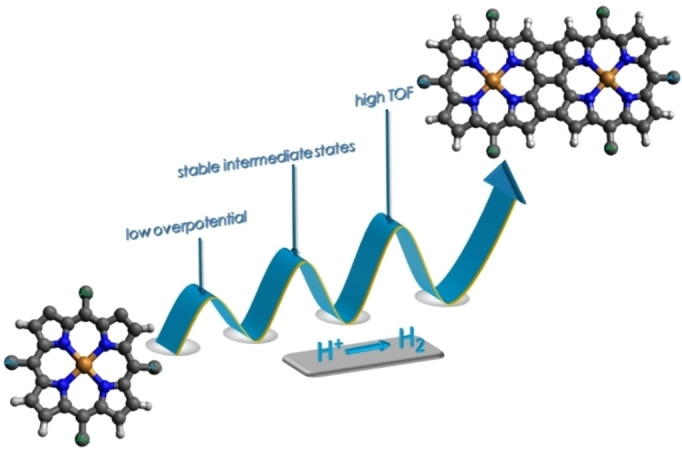
Advantages of fused porphyrin for electrocatalytic studies.

While metal complexes of monoporphyrins have been exploited extensively for electrocatalytic HER, oxygen evolution reaction (OER), and CO_2_ reduction reactions, similar studies comprising bimetallic fused porphyrins are limited to a single example (see below). Fused porphyrins are unique structural motifs, where two monoporphyrin units are connected via β–β, *meso*–*meso*, and β′–β′ triple covalent linkages.^[23]^ Metal complexes of fused porphyrins are promising candidates towards electrochemical HER attributed to their ability to undergo reductions or oxidations at lower applied potentials compared to their monomeric counterparts and extensive delocalization of π‐electrons across the framework (Figure 1). In this context, Moore and co‐workers for the very first time illustrated enhanced catalytic efficiency of doubly fused bimetallic copper porphyrins over the analogous nonfused monomeric copper porphyrin, in electrocatalytic HER.^[24]^

**Figure 2:**
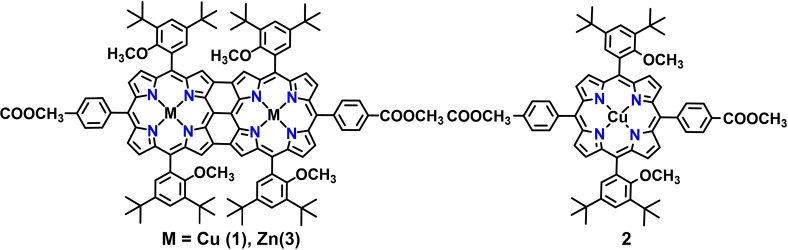
Molecular structures of the complexes studied in this work.

In the present contribution, we report the first example of HER catalysts based on the triply fused bimetallic copper porphyrin (**1**) and draw a comparison of the catalytic activity with analogous monomeric Cu‐porphyrin (**2**) and bimetallic zinc porphyrin (**3**) (Figure 2). Molecular identity of the complexes was confirmed via a combination of experimental techniques including cyclic voltammetry, electron paramagnetic resonance (EPR)/UV/Vis/near‐IR (NIR) spectroelectrochemistry, and density functional theory (DFT) calculations. Further, catalytic activity of the fused and monoporphyrin complexes towards electrochemical HER was investigated using trifluoroacetic acid (TFA) as a proton source. Different kinetic and thermodynamic factors governing the catalytic activity of the complexes were extracted from various electrochemical experiments. The primary objective of the work is the development of a molecular catalyst for electrochemical HER that will allow generation of high current densities while operating at low overpotentials and high turnover frequencies. To the best of our knowledge, this is the first report on the electrocatalytic activity of a metal complex with a triply fused porphyrin ligand, and the system presented here fulfils all the aforementioned sought‐after parameters for molecular electrocatalysts.

## Results and Discussion

Dinuclear and mononuclear copper complexes **1** and **2** were synthesized by insertion of copper into the free‐base fused porphyrin and its tetrasubstituted monomeric analogue by the reaction of copper acetate in chloroform/methanol (10 : 1) solvent mixture, at room temperature (**2**) and under refluxing condition (**1**). Purification of the crude material via column chromatography and recrystallization from chloroform/methanol yielded violet‐colored pure compounds in moderate yields. Molecular identity of all the precursor materials (complexes **3**, **5**, **6**, **7**) and the fused and monomeric porphyrin complexes (**1** and **2**) was confirmed via a combination of experimental techniques such as electron spray ionization mass spectrometry (ESI‐MS), proton nuclear magnetic resonance (^1^H NMR) spectroscopy, UV/Vis/NIR absorption, and EPR spectroscopy (Figures 5, S1–S10, S14, S15, and S17–S19, see the Experimental Section). The free‐base triply fused porphyrin exhibited characteristic NMR signals, as reported in the literature,^[25]^ in which peaks are shifted to higher fields due to the effect of π‐electron delocalization on the ring current. Crystals suitable for X‐ray diffraction (XRD) were obtained via slow diffusion of a benzene solution of the complex into ethanol; however, due to the poor quality of the crystals, reliable bond parameters could not be determined. Nevertheless, the core structure of the ligand along with the coordination mode of the metal is unequivocally established (Figure S11). From the molecular structure, a planar conformation of the fused ligand scaffold is observed, where two copper centers coordinate to four tetrapyrrolic nitrogen atoms of the individual porphyrin units, indicating coplanar arrangements of the copper and ligand framework.

**Figure 3:**
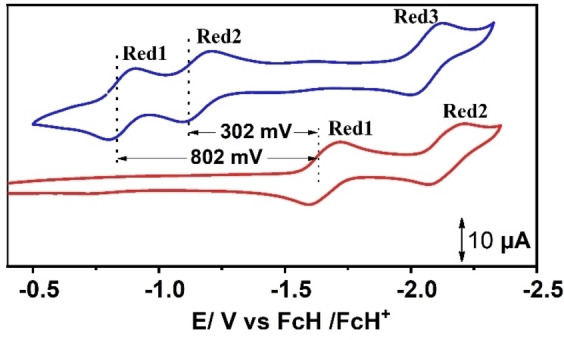
Cyclic voltammograms of 0.25 mm triply fused metalloporphyrin **1** (blue) and monomeric analogue **2** (red) in DMF. A larger potential window is shown in Figure S8.

Electrochemical properties of the complexes have been evaluated via cyclic voltammetric (CV) and differential pulse voltammetric (DPV) analysis in *N*,*N*,‐dimethylformamide (DMF) solution containing 0.1 m tetrabutylammonium hexafluorophosphate (*n*Bu_4_PF_6_) as the supporting electrolyte, at 100 mV s^−1^ scan rate. Cyclic voltammograms of the dinuclear complex **1** display multiple redox processes in the potential window spanning from +1 to −3.3 V (Figure S8 and Table S1). The cyclic voltammogram of the fused complex **1**, within the potential window ranging from −0.35 to −2.35 V, reveals three reversible redox features with half wave potentials, $E_{1/2}^{red1}$ =−0.86 V, $E_{1/2}^{red2}$ =−1.16 V, and $E_{1/2}^{red3}$ =−2.06 V, against the ferrocene/ferrocenium (FcH/FcH^+^) redox couple (Figures 3, S12, S13, and Table S1). On the other hand, monomeric complex **2** under identical experimental conditions display two reduction processes at $E_{1/2}^{red1}$ =−1.66 V and $E_{1/2}^{red2}$ =−2.12 V, respectively. The peak separation of (Δ*E*_p_) of around 85 mV between the cathodic and anodic waves of all redox processes in both complexes implies one‐electron redox processes (considering Δ*E*_p_=80 mV for the Fc/Fc^+^ redox couple). Notably, the first and second reduction processes in complex **2** appear at potentials that are significantly negative ($\Delta E_{1/2}^{red1}$ =802 mV and $\Delta E_{1/2}^{red2}$ =302 mV) compared to the first and second reduction processes in complex **1**, respectively.

This difference in peak potentials between triply fused dinuclear metalloporphyrin (**1**) and the monomeric analogue (**2**) in the present investigation is more pronounced than the previously reported Cu‐complexes of doubly fused and monomeric metalloporphyrins,^[24]^ which is consistent with our motivation for preparing these complexes. Considerable differences in the redox potentials in this case can be rationalized from the extensive delocalization of π‐electrons across the covalently linked porphyrin units. The corresponding Zn^II^‐fused porphyrin displayed identical behavior under similar experimental conditions The shift in potential is consistent with the increase in electrochemical highest occupied molecular orbital (HOMO)–lowest unoccupied molecular orbital (LUMO) gap from **1** to **2** (1.10 and 3.20 eV). Electrochemical data of the complexes have been summarized in Table S1. Thus, the bimetallic fused porphyrin, with significantly shifted redox potentials in comparison to the monomeric complex, encouraged further investigation of these complexes towards electrochemical HER.

**Figure 4:**
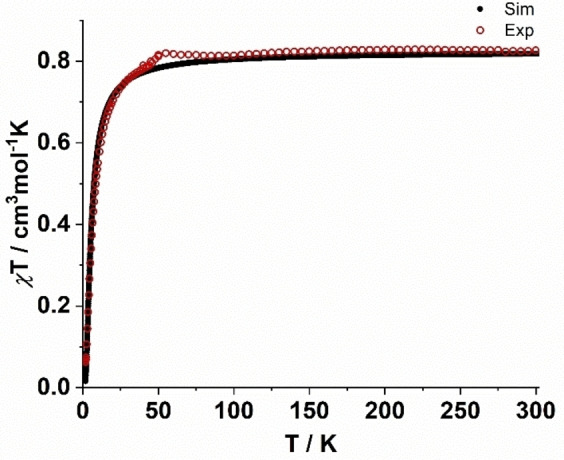
Magnetic susceptibility temperature product as a function of temperature, recorded on a powder sample of **1** in the temperature range 1.8–300 K.

**Figure 5:**
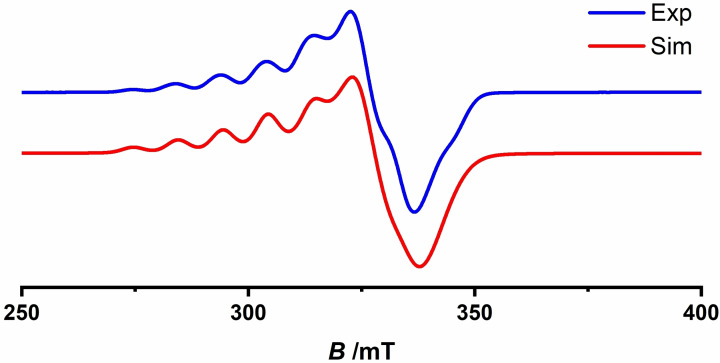
Experimental (blue) and simulated (red) EPR spectra of the complex **1** at 98 K.

EPR spectroscopic and variable temperature magnetic susceptibility (*χT*) measurements were performed to investigate the nature of the magnetic interactions between copper centers in complex **1** and **2** (Figure 4). The room‐temperature *χT* value of 0.85 cm^3^ mol^−1^ K corresponds to that expected for two uncoupled spin doublets with *g*=2.13 (Figure 5). On cooling, the *χT* value remains essentially constant down to until 40 K, following which a sharp drop in the value was observed reaching a *χT* value of 0.15 cm^3^ mol^−1^ K at the lowest temperatures.

The exchange coupling constant of *J*=−6.69 cm^−1^, obtained via least‐squares fitting of the Bleaney–Bowers equation^[26]^ using spin‐Hamiltonian *Ĥ*=−2*JŜ*_1_ *Ŝ*_2_, agrees well with the literature reported data.^[27]^ Furthermore, the value of the magnetic susceptibility (*χT*) was found to be consistent with the EPR derived parameters (*g*‐values, see below).

EPR spectra of pure solid **1** at 98 K revealed a broad resonance line containing a multiline pattern due to hyperfine coupling of the electron spin (*S*=1) to both ^63,65^Cu nuclei (*I*=3/2), consistent with the previously reports (Figures 5, S15, and Table S2).^[28]^ The best fit to the experimental EPR data was obtained by fitting the spectra obtained from a spin‐Hamiltonian consisting of an *S*=1 spin coupled to two equivalent ^63,65^Cu^II^ nuclei (*I*=3/2). The good agreement between the simulated and experimental spectra allowed determination of the rhombic *g*‐matrix, with *g*‐values (*g*_x_, *g*_y_, *g*_z_) of 2.06, 2.00, 2.22. The *g_z_* region shows a partially resolved hyperfine pattern consisting of seven lines with a 1 : 2 : 3 : 4 : 3 : 2 : 1 under this condition has a value of

lines clearly resolved), typical of hyperfine interaction with two equivalent ^63,65^Cu^II^ nuclei (*I*=3/2). The associated *A*_z_‐value=308 MHz is roughly half of the one observed for the monomer, which is indicative of an exchange coupled *S*=1 state.^[29]^ The absence of half‐field transitions (ΔMs=±2) even at lower temperature implies weak zero‐field splitting interaction, despite the triplet state spin structure, which has also been reported in the literature for diporphyrins with large center to center distances.^[30]^ Thus, rhombic distortion of the *g*‐values and lower *A*_z_‐value in the case of **1** indicate sharing of two unpaired electrons between the two porphyrin units. The spectrum of **1** arises from the thermally populated triplet state (*S*=1) above the singlet ground state (*S*=0).

EPR spectra of mononuclear copper porphyrin (**2**) measured at 98 K display a multiline pattern arising from the hyperfine interaction to a single ^63,65^Cu (*I*=3/2) and four ^14^N (*I*=1) nuclei (Figure S15 and Table S2). The experimental spectrum has been reasonably fitted through simulation of an *S*=1/2 spin‐Hamiltonian with hyperfine interaction to the previously described nuclei with an axial *g*‐matrix. The values of $g_{∥}=2.19$ ($A_{∥}=596\;\;MHz$ ) and $g_{⊥}=2.045$ (*A* perp=62 MHz) agree well with reported literature data for related Cu‐monoporphyrin complexes.^[31]^

The small but non‐negligible exchange constant, as well as the seven‐line hyperfine pattern in the EPR spectrum of **1** reflects the delocalization of the spin caused by, and underlines the important role of, the conjugated bond between individual porphyrin units, which results in medium‐range antiferromagnetic coupling.

**Figure 6:**
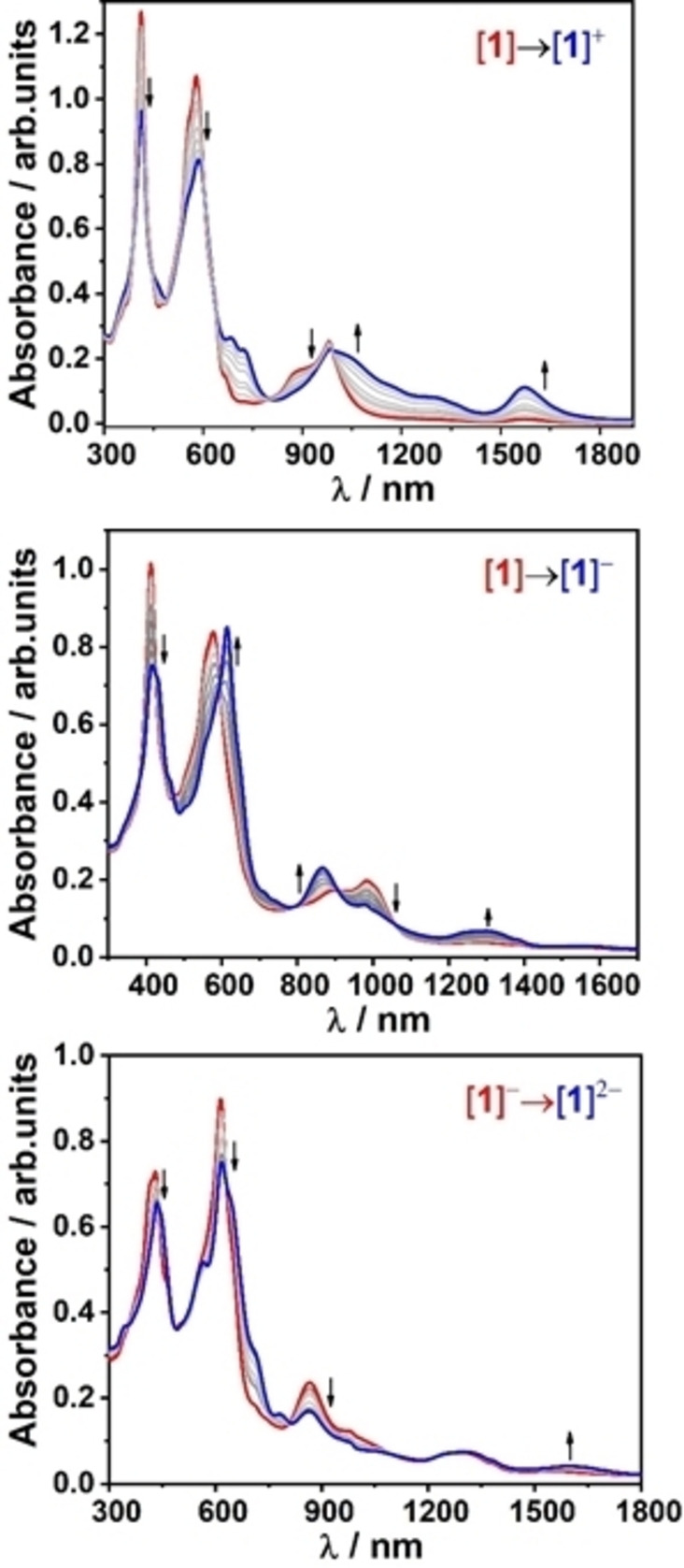
UV/Vis/NIR spectroelectrochemical responses of **1** ^*n*^ in DMF/0.1 m *n*Bu_4_PF_6_ solution.

Successive changes in the electronic and vibrational structure of the complexes **1**, **2**, and **3** upon consecutive reductions have been evaluated via UV/Vis/NIR spectroelectrochemical studies (Figures 6, S17–S19, and Table S3). UV/Vis/NIR absorption profiles of complexes **1** and **2** in DMF reveal significant differences with three distinct absorption bands in **1**, in contrary to two bands in **2** (Figures 6, S17, S18, and Table S3).^[25]^

Monomeric complex **2** exhibited absorption bands at 417 and 536 nm, respectively, corresponding to Soret and Q‐band transitions. In contrast, the Soret‐like transitions in complex **1** are split into two bands at 414 and 576 nm (band I and II, respectively), owing to the excitonic coupling between the individual porphyrin units. On the other hand, the Q‐band like features in **1** are significantly red shifted at 887 and 986 nm compared to the Q‐bands of **2**, attributed to the extensive conjugation between the diporphyrin π‐electron systems.

In the case of complex **1**, upon reduction, the Soret bands at 414 and 576 nm underwent bathochromic shifts to 415 and 611 nm with distinct isobestic points, inferring stable conversion without an involvement of transient species or decomposition products. On the other hand, the Q‐band transitions display hypsochromic shifts to 865 nm along with an appearance of a new band at 1293 nm possibly due to the intra‐ligand charge transfer transitions. The monomeric complex **2** upon reduction exhibits similar behavior, with an appearance of a band at 877 nm in addition to the Q‐band transition at 666 nm. Further reduction of the monoanionic species leading to dianionic species **1** ^2−^, resulted in the growth of a low energy NIR band at around 1600 nm with concomitant reduction in intensity of the existing absorption bands. Notably, application of starting potential of 0 V to the in‐situ generated species resulted in the recovery of the UV/Vis spectrum identical to the native species, underlining the reversibility of the redox processes. Spectroelectrochemical response of **1** upon 1 e^−^ oxidation resulted in the decrease in intensity of the Soret bands and blue shift of the Q‐bands to 688 and 723 nm, in addition to formation of new low‐energy band at 1572 nm, with clearly defined isobestic points, implying formation of porphyrin π‐cation radical **1** ^+^. Successive oxidation of the intermediate monocationic species **1** ^+^ to the respective dication resulted in further decrease in intensity of the existing absorption bands.

**Figure 7:**
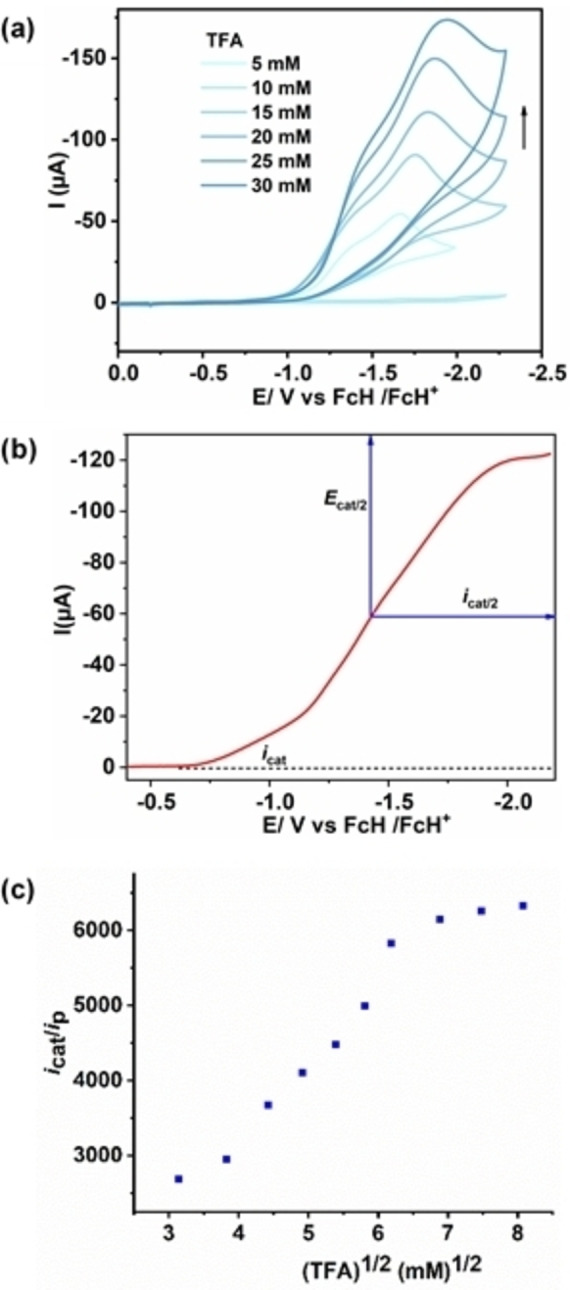
(a) Cyclic voltammetric responses of 0.01 mm DMF solution of **1** with increasing concentration of TFA (condition: 100 mV s^−1^ scan rate, under Ar, 0.1 m *n*Bu_4_PF_6_). (b) Cyclic voltammogram of 0.01 mm DMF solution of **1** containing 15 mm of TFA and 0.1 m of electrolyte at 300 mV s^−1^ scan rate, showing an ideal S‐shape plot. (c) Plot of *i*_cat_/*i*_p_ versus square root of the concentration of the TFA.

Catalytic activity of the fused and monoporphyrin complexes **1** and **2** towards electrochemical HER was investigated in DMF with 0.1 m *n*Bu_4_PF_6_ as the supporting electrolyte under Ar in the presence of TFA (p*K*_a_=6.1 in DMF) (Figure 7a). All the measurements have been performed in a three‐electrode configuration electrochemical cell equipped with glassy carbon working electrode, platinum wire as a counter electrode, and Ag‐wire as a pseudoreference with FcH/FcH^+^ redox couple as an internal reference. Significantly, upon addition of varying concentrations of TFA to 0.01 mm solutions of the complexes, a large electrocatalytic current associated with an irreversible cathodic wave appeared at an onset potential of −0.96 V, which reached maximum at −1.94 V, with a half wave potential of −1.42 V (Figures 7a and S20). Notably, appearance of catalytic reduction waves near to the **1** ^2−^/**1** ^−^ (second reduction) redox process indicated possible involvement of doubly reduced species in HER (Figure S21). This is corroborated by the lack of significant changes in the reversible nature of the first reduction wave before and after addition of TFA (Figure S21). From the thermodynamic potential required for the reduction of TFA in DMF (≈‐0.94 V), the overpotential for HER for **1**, was calculated to be around 480 mV (Figure 7b).

**Figure 8:**
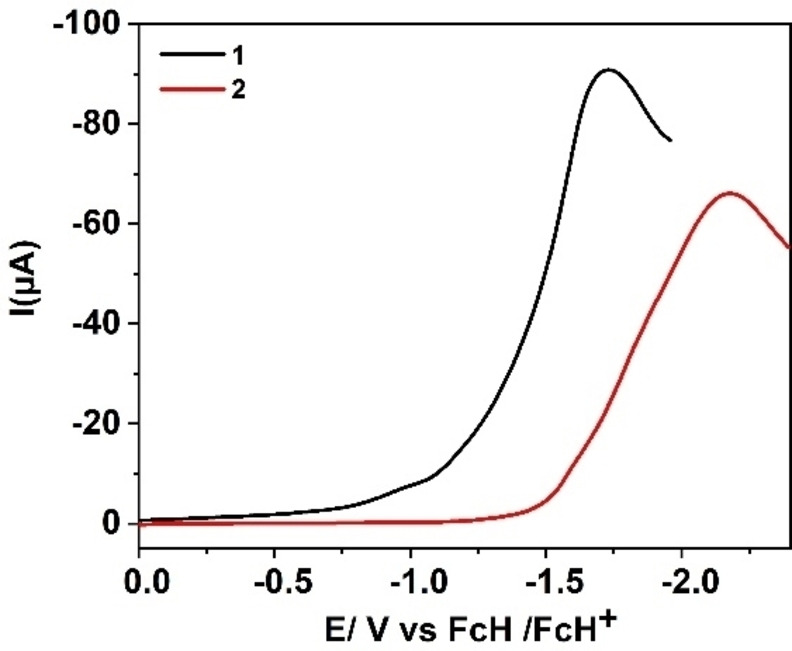
LSV of 0.01 mm DMF solutions of **1** (black) and **2** (red) with 0.1 m *n*Bu_4_PF_6_ in the presence of 15 mm of TFA at 100 mV s^−1^.

Consistent with the analogy proposed by Saveant and co‐workers,^[32]^ an ideal *S*‐shaped curve was obtained at higher scan rate of 300 mV s^−1^ and 15 mm of acid concentration (Figure 7b). The ideal nature of the voltammogram at relatively higher scan rate and acid concentrations refer to the typical condition where concentration of the substrate at the electrode surface is equal to the concentrations of the bulk solutions (i.e., pure kinetic conditions with no substrate depletion). Under this condition, the fraction of the activated catalyst at the electrode surface is unity; therefore, the observed rate constant (*k*_obs_), which is equivalent to the maximum turnover frequency (TOF_max_), can be calculated from the plateau current of the S‐shaped curve.^[32]^ The linear dependence of peak current (*i*_p_) with the square root of the scan rate (*ν* ^1/2^) confirmed catalysis in the diffusion‐controlled electrochemical regime under these experimental conditions (Figure S22). The catalytic current (*i*_cat_) associated with the S‐shaped catalytic wave increased linearly with scan rate following a first‐order rate dependence. Importantly, the linear dependence of *i*_cat_ over scan rate no longer holds when scan rate exceeds 0.5 V s^−1^ indicating proximity to the saturation point (Figure S23). Further, increasing TFA concentrations beyond 40 mm led to the saturation point, beyond which addition of further acid had almost zero effect on the catalytic current (Figure 7c) indicating zero‐order rate dependence of *k*_obs_ on acid concentration, resulting in the determination of rate constant. Notably, under identical experimental conditions (0.01 mm of catalyst and 15 mm of acid), **2** displayed an irreversible catalytic wave with a significant cathodic shift (≈600 mV) in the onset potential, compared to **1**, underlining higher activity of **1** over **2** (Figure 8). Moreover, comparison of the catalytic efficiency under identical experimental conditions (0.01 and 15 mm of catalyst and acid, respectively) revealed considerably higher overpotential (≈800 mV) in the case of **2** as compared to **1** for HER. Thus, catalyst **1** lowers the overpotential by around 320 mV compared to **2** for catalytic proton reduction, indicating an enhanced activity of the bimetallic framework.

Since **1** possesses two individual porphyrin units linked together, evaluation of the electrochemical HER activity was also performed using twice as much concentration (0.02 mm) of **2** against 0.01 mm of **1** with a fixed acid concentration (15 mm) (Figure S19).

Linear sweep voltammograms (LSV) of 0.01 and 0.02 mm solutions of **1** and **2**, respectively, under otherwise identical experimental conditions, revealed that even at higher concentration complex **2** exhibit catalytic activity at potentials of around 300 mV negative (higher overpotentials) to that of the lower concentration solution of complex **1** (Figure S24). It clearly suggests better catalytic efficiency of **1** over **2** towards HER. Importantly, addition of 15 mm TFA to a DMF solution containing 0.1 m *n*Bu_4_PF_6_ in the absence of catalyst showed no catalytic currents under otherwise similar experimental conditions (scan rate: 100 mV s^−1^) (Figure S25). On the other hand, electrochemical measurements containing 0.01 mm solution of the corresponding Zn‐complex **3** in presence of TFA (15 mm) under identical experimental conditions (0.1 m *n*Bu_4_PF_6_, scan rate: 100 mV s^−1^) displayed around 400 mV cathodic shift in onset potential compared to **1**, highlighting the influence of the metal on the catalytic activity (Figure S25).

Different control experiments were performed to rule out possible detrimental side‐effects of various components in the catalytic processes. Initially, to eliminate the effect of catalyst deposition on the electrode surface during electrocatalytic process, the glassy carbon electrode used in HER was taken out of the solution after catalysis and rinsed carefully to remove weakly adsorbed species and dipped into a solution of DMF containing only 15 mm of TFA and 0.1 m of *n*Bu_4_NPF_6_ in the absence of a catalyst. Absence of any catalytic current above the background obtained from the rinse test rules out any role of electrode‐adsorbed active catalysts in HER (Figure S26). A control experiment using an Hg‐pool working electrode was conducted to detect possible involvement of metal nanoparticles in proton reduction, since some metal particles are inactivated in the presence of Hg^0^ due to adsorption or amalgamation, resulting in loss of catalytic activity. However, no such differences in catalytic current in the presence or absence of the Hg‐pool electrode were found, eliminating any influence of metal nanoparticles in catalytic activity, underlining the role of molecular catalysts in HER (Figure S27).^[33]^ The influence of leached platinum particles that could detach from the platinum counter to HER could also be rejected based on no difference in catalytic activity when using a glassy carbon counter electrode instead of platinum (Figure S28).^[34]^ Acid‐stability of the compound **1** was verified from the UV/Vis/NIR spectroscopic measurements of **1** in a 1 : 1 mixture of TFA/DMF solution. However, the absorption spectra of the acidic solution of **1** did not show any noticeable change even after prolonged periods of 24 h in the dark (Figure S29). ESI‐MS analysis of the solution confirmed that the tetrapyrrolic core of **1** remains intact and demetallation does not occur.

After establishing the catalytic activity of **1** in electrochemical HER, we calculated the catalytic rate constant (*k*_obs_), also referred to as TOF, to compare the efficiency of the catalysts with literature‐reported molecular catalysts. *k*_obs_ was calculated from the ratio of catalytic current (*i*_cat_) and peak current (*i*_p_) following Equation S3 (Figures 7c, S30, and Table S4) at different acid concentrations and a constant scan rate of 300 mV s^−1^.[^32^, ^35^] A plot of *k*_obs_ against TFA concentrations disclosed first‐order dependence of rate constant on the concentration of TFA (Eq. S3 and Figure S30), when *i*_cat_ is independent of scan rate (Eq. S1 and S3) and directly proportional to square root of TFA. At acid concentrations beyond 40 mm, when increasing the concentration of acid has no effect on the plateau current (*i*_cat_), *k*_obs_ becomes zero‐order with respect to TFA concentration (Eq. S4). The rate constant calculated under this condition has a value of 0.5×10^7^ s^−1^, which is almost twice the previously reported doubly fused copper porphyrin complex.^[24]^ Although direct comparison among HER catalysts is difficult, given the complexity and diversity associated with various experimental conditions, the reported rate constant for **1** is remarkably high, and to the best of our knowledge is among the highest reported for first‐row transition metal complexes in the literature (Table 1). Notably, this high rate constant is achieved at a fairly low overpotential (Table 1).

**Table 1:** Summary of selected homogeneous 3d metal‐containing molecular electrocatalysts for proton reduction.

Complex	Conditions (solvent and proton source)	*k*_obs_ [s^−1^]	*η* [mV]	Faradaic efficiency [%]	Current range	Ref.
[Ni(P^Ph^_2_N^Ph^)_2_](BF_4_)_2_	[(DMF)H]^+^OTf^−^/1.2 m H_2_O	10^6^	625	99	μA	[4]
[Ni(P^Ph^_2_N^C^_6_^H^_4_^X^_2_)_2_](BF_4_)_2_	[(DMF)H]^+^OTf^−^/1.2 m H_2_O	1850	370	–	μA	[36]
[Co(DO)(DOH)pnBr_2_]	CH_3_CN/*p*‐cyanoanilinium tetrafluoroborate	–	230	99	μA	[37]
FeTPP‐Cl	DMF/Et_3_NHCl	4×10^5^	–	95	μA	[18]
Co‐hangman porphyrin	CH_3_CN/benzoic acid	–	920	80	μA	[38]
Cu_2_FP	CH_2_Cl_2_/TFA	2×10^6^	–	–	μA	[24]
Ni‐C_6_F_5_Porphyrin	CH_2_Cl_2_/AcOH	–	240	96	μA	[39]
[Ni‐porphyrin‐L]_2_	CH_2_Cl_2_/AcOH	–	–	95	μA	[15]
[Cp*Co‐MIC(Cl)]PF_6_	CH_3_CN/AcOH	4×10^2^	130	80(±20)	μA	[40]
**1**	DMF/TFA	0.5×10^7^	480	97	mA	this work

**Figure 9:**
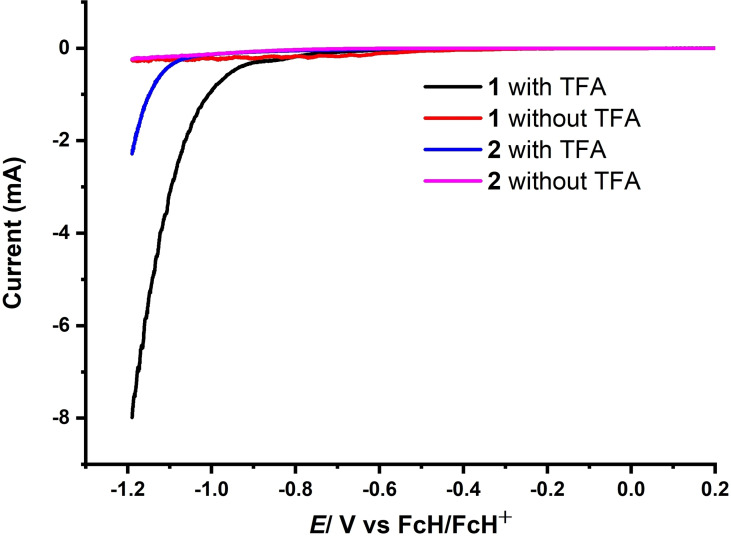
LSV of 0.06 and 0.12 mm DMF solutions of **1** and **2** before bulk electrolysis.

To quantify the amount of hydrogen evolved, controlled potential electrolysis of **1** and **2** was carried out in a gas‐tight H‐type cell separated by a microporous membrane. LSV experiments, carried out with 0.06 and 0.12 mm DMF solutions of **1** and **2**, revealed better efficiency of the catalyst **1** in comparison to **2**, as shown by the lower onset potential and nearly 4‐fold increase in the current of the former compared to the latter (Figure 9).

**Figure 10:**
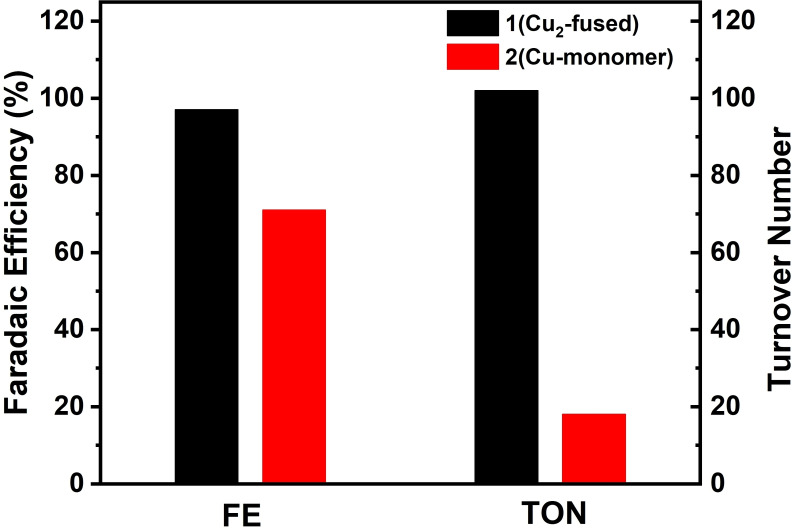
Comparison of faradaic efficiency and turnover number for **1** and **2**.

Bulk electrolysis measurements were performed with 0.06 and 0.12 mm solutions of **1** and **2**, respectively, in 30 mL DMF solution containing 0.1 m TFA, at −1.05 V, under Ar atmosphere. During the electrolysis process, constant cathode potential was maintained and the amount of H_2_ formed was detected via gas chromatographic (GC) analysis (Figures S31 and S32). Faradaic efficiency of **1** and **2** upon controlled potential electrolysis conducted for 30 min was calculated to be 96.6 and 71.2 %, respectively, underlining the enhanced catalytic performance of **1** compared to **2** towards electrochemical HER (Figure 10). Turnover numbers for **1** and **2** were calculated to be 102 and 18.4 (Figure 10), which indeed supported increased catalytic activity/stability of **1** against **2**. After each electrolysis measurement, the carbon paper working electrode was removed from the electrolyte, rinsed three times with DMF, and reinstalled to perform blind experiments. LSV were recorded with fresh solutions of DMF containing 0.1 m *n*Bu_4_PF_6_ and 0.1 m TFA, without any catalysts. Lack of any substantial background current ruled out any involvement of adsorbed catalytic species such as Cu nanoparticles deposited onto the electrode surface during the electrocatalytic process (Figures S33 and S34). This observation was further supported by the scanning electron microscopy (SEM)/energy‐dispersive X‐ray spectroscopy (EDX) experiment of the carbon paper after the bulk electrolysis for the complexes **1** and **2**, where copper oxide was not detected on the surface of the carbon paper, indicating the electrochemical stability of the catalysts (Figure S35). UV/Vis/NIR spectra of the catalysts before and after electrolysis exhibited no changes in the positions of the Soret and Q‐bands of complex **1** (Figure S36), confirming the stability of the catalysts during electrocatalytic H_2_ evolution.

**Figure 11:**
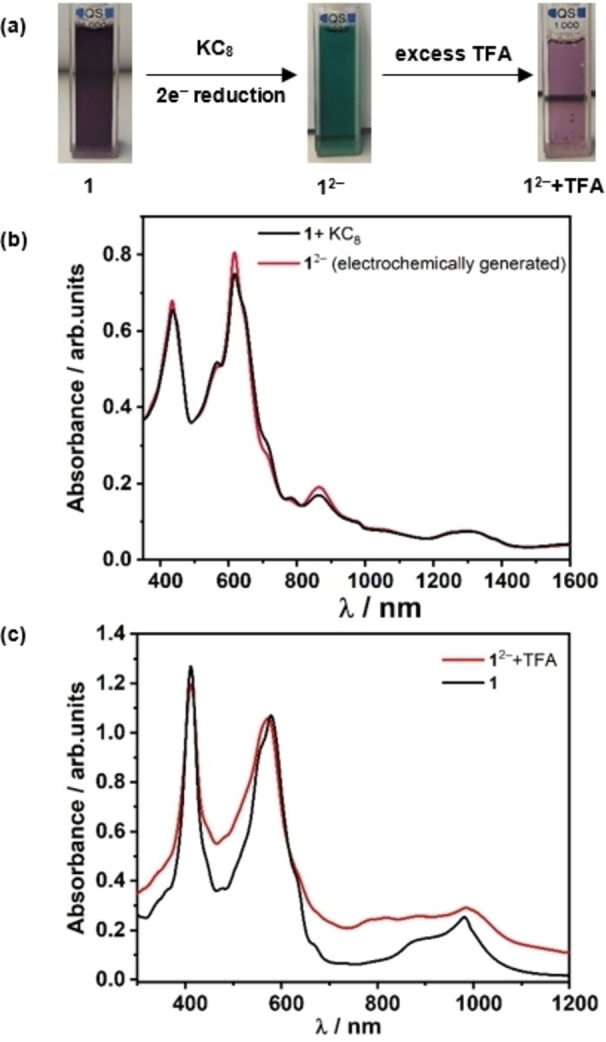
(a) Change in color of the DMF solution of **1** upon two‐electron reduction (deep green) and reaction of the reduced species with TFA to regenerate the native species. (b) UV/Vis/NIR absorption spectra of the electrochemically (red) and chemically (black) generated doubly reduced species. (c) Absorption spectra of the doubly reduced species with excess TFA (red) and native species **1** (black).

**Figure 12:**
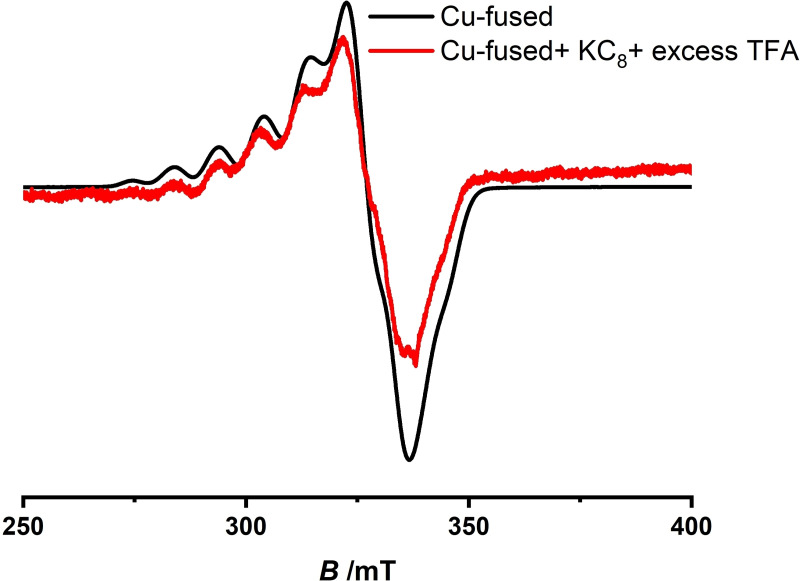
EPR spectra of the reaction of doubly reduced species (**1** ^2−^) with excess TFA (red) and native species **1** (black) performed at −175 °C.

To gain insight into the mechanistic pathway of electrochemical HER with **1**, one‐ and two‐electron reduced species of **1** were generated in situ (electrochemically) and through chemical reduction. Treatment of **1** with a stoichiometric amount of cobaltocene (CoCp_2_) in CH_2_Cl_2_, resulted in the formation of a light green colored one‐electron reduced species **1** ^−^, confirmed from the comparison of UV/Vis/NIR spectra of chemically (Figure S37) and in‐situ generated species (Figure 6). Addition of TFA to **1** ^−^ leads to a new spectrum with simultaneous change in color of the solution from light green to yellow (Figure S38). Addition of large excess of TFA to this solution did not induce any significant spectral changes, implying lack of basicity of the one‐electron reduced species to drive the protonolysis further (Figure S37c). Thus, further reduction of **1** ^−^ is necessary to facilitate the catalytic process. Moreover, EPR spectroscopic analysis of the 1e^−^ reduced species (Figure S39) shows a signal corresponding to an isolated Cu^II^ center, and thus this species could be assigned as a localized Cu^II^/Cu^I^ species. The EPR spectrum of the resulting species after an addition of TFA suggests an organic radical, although the signal is weak and therefore could not be properly analyzed by simulation. At this point including a full spectroscopic characterization of all possible reduced intermediates is beyond the scope of this work. However, further studies related to the in‐detail analysis of these intermediates are underway in our laboratory. To generate the doubly reduced species **1** ^2−^, KC_8_ (2 equiv.) was added into the DMF solution of **1**, resulting in immediate change in color of the solution from violet to deep green, indicating formation of the desired doubly reduced species, as confirmed from the changes in the absorption spectra (Figure 11a). Similarity in the absorption spectrum of **1** ^2−^ generated either electrochemically or chemically, along with well‐defined isosbestic points confirms complete conversion of the native species to the doubly reduced form (Figure 11b). Importantly, treatment of the doubly reduced species with excess of TFA gave altered absorption spectra, which after some time slowly decayed back to regenerate the initial species **1**, as confirmed from the identical UV/Vis/NIR spectrum of the reduced species containing TFA with that of the initial form (Figure 11c). Conversion of the two electron reduced species to the initial species could also be followed from the noticeable change in color of the solution from deep green to violet (Figure 11a). EPR spectroscopic analyses of the one‐ and two‐electron reduced species and their reaction with TFA led to the same conclusions, wherein doubly reduced species **1** ^2−^, generated via chemical reduction, on treatment with excess of TFA, reverts to the initial species **1**, as observed from the identical EPR spectra (Figure 12). Similarity in the absorption spectra obtained from electrochemical and chemical reduction emphasizes identical redox behaviors of the transient species under chemical and electrochemical treatment. Thus, the aforementioned experimental observations strongly suggest possible involvement of the doubly reduced form, **1** ^2−^ as a catalytically active species towards electrochemical reduction of protons to hydrogen.

Based on the information obtained from the experimental analysis, we propose that the native species **1** undergoes stepwise two one‐electron reductions to form doubly reduced species **1** ^2−^, which subsequently reacts with protons generating a probable hydride intermediate. The intermediate species so formed, in presence of excess amount of TFA, reacts with a second proton to generate initial species along with the release of hydrogen. However, presence of two metals in the conjugated porphyrin system allows for a competitive scenario involving formation of a bimetallic hydride species. The corresponding hydride species at this point could not be isolated or detected spectroscopically. Investigation of the mechanistic pathway is currently under investigation in our laboratory to improve the efficiency and compatibility of the fused metalloporphyrins towards various electrochemical small molecule activation reactions.

## Conclusion

Bimetallic copper complex (**1**) featuring a triply fused porphyrin framework was synthesized to generate a highly efficient molecular catalyst for electrocatalytic proton reduction. As indicated by cyclic voltammetric analysis, reduction processes in the case of fused metalloporphyrin occurred at a lower applied bias potential, compared to the nonfused complex, with a difference of around 800 mV between the first reduction processes of each complex. Such a large margin of difference in reduction potentials is attributed to the extended delocalization of electrons within the fused porphyrin framework, allowing easy tunability of the redox properties as compared to the well‐known methods of using electron deficient functional groups to fine‐tune the electrochemical potential in energy driven processes. Notably, **1** exhibited increased activity and lower onset potential towards proton reduction, compared to **2**. Consistent with the enhanced electrocatalytic activity, **1** significantly reduced the overpotential (by ≈320 mV) compared to **2**. These results taken together suggest a positive impact of the fused scaffold over monomeric porphyrins on proton reduction, which is also reflected in significantly higher faradaic efficiency of the dinuclear complex (97 % for **1** and 71 % for **2**). Further, the observed rate constant (*k*_obs_) of 0.5×10^7^ s^−1^ is the highest reported rate constant so far among molecular electrocatalysts of the first row of transition metals for hydrogen evolution reaction (HER), and this high rate constant is already achieved at a fairly low overpotential. Although further analysis regarding identification of the mechanistic pathway as well as probable hydride intermediates is necessary, we were able to prove the involvement of two‐electron reduced species in electrochemical HER. To the best of our knowledge, this is the first time a metal complex (any metal) of a triply fused biporphyrin ligand has been used as a molecular electrocatalyst. Considering the excellent catalytic parameters obtained from the investigation (low overpotential, stability of the molecular catalyst, high turnover frequency and number, unprecedented current densities, and high rate constants), we expect metal complexes of triply fused biporphyrin ligands to play a very important role in energy‐related electrocatalytic work in the future.

## Experimental Section

### Materials

Unless otherwise stated, all experiments were carried out under an argon atmosphere using standard Schlenk techniques or in a MBraun Unilab SP GloveBox. Pyrrole was obtained from Sigma Aldrich and distilled prior to use. Commercially available chemicals like 3,5‐di‐*tert*‐butyl‐2‐methoxybenzaldehyde (abcr GmbH), Sc(OTf)_3_ (Sigma Aldrich), 2,3‐dichloro‐5,6‐dicyano‐1,4‐benzoquinone (abcr GmbH), and Cu(OAc)_2_ ⋅ 4H_2_O (Sigma Aldrich) were used without further purification. KC_8_ was synthesized according to a literature procedure.^[41]^ Dry DMF was purchased from Sigma Aldrich (99.8 % extra dry) and was used as received. Other solvents were purified using the MBRAUN MB‐SPS‐800 solvent system. All of the solvents were degassed by standard techniques prior to use. Column chromatography was conducted using silica from MachereyNagel (Silica 60, 0.04–0.063 mm). ^1^H NMR spectra were recorded on Bruker Avance 250 MHz and Bruker Avance 400 MHz spectrometers at 20 °C. Chemical shifts are reported in ppm (relative to the tetramethylsilane signal) with reference to the residual solvent peaks.^[42]^ MS was performed on Bruker Daltonics Microtof‐Q (ESI) at 0.4 bar, 200 °C, dry gas flow 4.0 L min^−1^ with a set capillary of 4500 V, end plate offset −450 V in positive mode and at 1.0 bar, set capillary 2200 V in negative mode) or Varian MAT 711 (EI at 70 eV) mass spectrometers. UV/Vis/NIR spectra were recorded with an on a J&M TIDAS spectrometer instrument.

### Electrochemistry

Cyclic voltammograms were recorded with a PalmSens4 potentiostat or with a Metrohm Autolab PGSTAT101 by working in anhydrous and degassed DMF (99.8 % extra dry, Sigma Aldrich) with 0.1 m *n*Bu_4_PF_6_ (dried, >99.0 %, electrochemical grade, Fluka) as the supporting electrolyte. Concentrations of the complexes were about 1×10^−4^ m. A three‐electrode setup was used with a glassy carbon working electrode, a coiled platinum wire as counter electrode, and a coiled silver wire as a pseudo‐reference electrode. The ferrocene/ferrocenium couple was used as an internal reference. Electrochemical proton reduction experiments were carried out in distilled and degassed DMF solution of 1×10^−4^ m catalysts with 0.1 m *n*Bu_4_PF_6_ as supporting electrolyte and varying amounts of TFA. The experiments were carried out with 3 mm diameter glassy carbon working electrode which was polished with 0.3 μm alumina slurry prior to each scan. Coiled platinum wire and coiled silver wire were used as counter and pseudo‐reference electrodes, respectively. All the measurements in electrochemical HER were referenced against ferrocene/ferrocenium redox couple.

### Bulk electrolysis measurements

Bulk electrolysis measurements were performed in a two‐compartment cell divided by microporous membrane (Celgard® 2325). A 0.06 mm DMF solution of 1 or 0.12 mm of 2 containing 0.1 m *n*Bu_4_PF_6_ and 0.1 m of TFA was sparged with N_2_ before measurements. The measurements were performed with Origalys (OGF500) using a carbon paper (1×1 cm^2^ as catalytic area) as a working electrode, platinum foil as a counter electrode and a coiled silver wire as a pseudo‐reference electrode. Samples of the headspace (4 mL) were taken using a gastight syringe. The headspace composition was analyzed using a GC equipped with molecular‐sieve columns and dual thermal conductivity detector (TCD) and flame ionization detector (FID) using helium as a carrier gas.

### Spectroelectrochemistry

Spectroelectrochemical measurements were carried out in an optically transparent thin‐layer electrochemical (OTTLE)^[43]^ cell (CaF_2_ windows) with a platinum mesh working electrode, a platinum‐mesh counter electrode, and a silver‐foil pseudo‐reference electrode. Anhydrous and degassed DMF (99.8 % extra dry, Sigma Aldrich) with 0.1 m *n*Bu_4_PF_6_ as the electrolyte was used as the solvent. The catalyst under investigation was dissolved into the 0.1 m *n*Bu_4_PF_6_ electrolyte solution and the OTTLE cell was then filled with 0.2 mL of the resulting solution and packed under Ar atmosphere. UV/Vis/NIR spectra of the in‐situ generated species via application of desired potential corresponding to the specified redox processes were recorded simultaneously under specified time interval.

### Electron paramagnetic resonance

EPR spectra at X‐band frequency (≈9.5 GHz) were obtained with a Magnettech MS‐5000 benchtop EPR spectrometer equipped with a rectangular TE 102 cavity and TC HO4 temperature controller. The measurements were performed in synthetic quartz glass tubes. Spectral simulations were performed using the EasySpin package^[44]^ running in Matlab R2018b.

### Synthesis

Methyl 4‐formylbenzoate was synthesized following a reported procedure,^[45]^ by reacting 4‐formylbenzoic acid (1.0 equiv.) with methyl iodide (1.2 equiv.) and K_2_CO_3_ (1.0 equiv.) in DMF at 60 °C for 2–4 h. The reaction was quenched with H_2_O. The crude products were extracted with DCM, the organic phase was washed with H_2_O and dried over Na_2_SO_4_. Chromatographic purification in a small silica column (DCM) was performed to remove yellow impurities. The identity and purity of the product was confirmed by NMR spectroscopy. 3,5‐Di‐*tert*‐butyl‐2‐methoxybenzaldehyde,^[46]^ dipyrromethane,^[47]^ and 5‐(4‐methylcarboxyphenyl)‐dipyrromethane^[48]^ were synthesized following the literature reported method.^1^H NMR spectra were in complete agreement with the published data.

**Synthesis of 5**: 3,5‐Di‐*tert*‐butyl‐2‐methoxy benzaldehyde (1186 mg, 4.78 mmol), 5‐(4‐methylcarboxyphenyl)‐dipyrromethane (669 mg, 2.39 mmol), and dipyromethane (350 mg 2.39 mmol) were dissolved in DCM and degassed for 15 min. TFA (0.38 equiv.) was added dropwise, and the solution was stirred for overnight in the dark. 2,3‐Dichloro‐5,6‐dicyano‐1,4‐benzoquinone (DDQ) (1.6 g, 3 equiv.) was added and further stirred for 3 h. The reaction was quenched with triethylamine and solvent was removed under reduced pressure. The crude product was purified by silica gel column chromatography and the desired product was eluted by using DCM/hexane (1 : 1) solvent mixture. Second fraction was collected, and removal of solvent yielded reddish‐pink colored pure compound **5**. Yield: 189 mg (9 %). ESI‐MS (M^+^H^+^): *m*/*z*=calc. for: C_58_H_65_N_4_O_4_; 881.5000, found: 881.4987. ^1^H NMR (250 MHz, chloroform‐d): *δ*=10.20 (s, 1H), 9.34 (d, *J*=4.6 Hz, 2H), 9.11 (d, *J*=4.6 Hz, 2H), 9.00 (d, *J*=4.7 Hz, 2H), 8.80 (d, *J*=4.8 Hz, 2H), 8.43 (d, *J*=8.1 Hz, 2H), 8.32 (d, *J*=8.0 Hz, 2H), 7.86 (d, *J*=2.4 Hz, 2H), 7.77 (d, *J*=2.5 Hz, 2H), 4.11 (s, 3H), 2.58 (s, 6H), 1.68 (d, *J*=6.5 Hz, 18H), 1.47 ppm (p, *J*=2.6 Hz, 18H).

**Synthesis of 7**: For Zn^II^ metallation, a solution of Zn(OAc)_2_ ⋅ 2H_2_O (49 mg, 0.226 mmol) in MeOH was added to a solution of **5** (100 mg, 0.113 mmol) in CHCl_3_, and the resulting mixture was stirred for 1 h. The progress of the reaction was monitored by thin‐layer chromatography (TLC). After complete conversion was achieved, the mixture was poured into water, and was extracted with CHCl_3_. The organic layer was separated, and the combined extracts were repeatedly washed with water and brine and dried over anhydrous Na_2_SO_4_. The solvent was removed, and the residue was washed with MeOH to provide pure material **7** that can be used without further purification. Yield: 106 mg (99 %). ESI‐MS (M^+^H^+^): *m*/*z*=calc. for: C_58_H_63_N_4_O_4_Zn; 943.4135, found: 943.4131. ^1^H NMR (250 MHz, chloroform‐d): *δ*=10.25 (s, 1H), 9.42 (d, *J*=4.4 Hz, 2H), 9.20 (dd, *J*=4.5, 2.0 Hz, 2H), 9.09 (dd, *J*=4.7, 1.5 Hz, 2H), 8.90 (d, *J*=4.7 Hz, 2H), 8.46–8.16 (m, 4H), 7.93 (dd, *J*=15.3, 2.4 Hz, 2H), 7.81–7.73 (m, 2H), 4.10 (s, 3H), 2.50 (d, *J*=3.0 Hz, 6H), 1.66 (s, 18H), 1.48 ppm (dd, *J*=3.5, 1.1 Hz, 18H).

**Synthesis of 6**: 3,5‐Di‐*tert*‐butyl‐2‐methoxy benzaldehyde (1186 mg, 4.78 mmol),5‐(4‐methylcarboxyphenyl)‐dipyrromethane (669 mg, 2.39 mmol), and dipyromethane (350 mg 2.39 mmol) were dissolved in DCM and degassed for 15 min. TFA (0.38 equiv.) was added dropwise, and the solution was stirred for overnight in the dark. DDQ (1.6 g, 3 equiv.) was added, and the reaction mixture was allowed to stir for 3 h. The reaction was quenched with triethylamine and the solvent was removed under reduced pressure. The crude product was purified via silica gel column chromatography. The desired product was eluted with DCM/hexane solvent mixture (4 : 1 to pure DCM). Third fraction was collected, and solvent was removed under reduced pressure. Crystallization of the compound from chloroform/methanol mixture resulted in red colored pure compound **6**. Yield: 218 mg (19 %). ^1^H NMR (250 MHz, chloroform‐d): *δ*=8.89 (d, *J*=4.6 Hz, 3H), 8.72 (d, *J*=5.5 Hz, 4H), 8.43–8.13 (m, 8H), 7.85 (d, *J*=2.4 Hz, 1H), 7.80 (d, *J*=2.3 Hz, 1H), 7.74 (d, *J*=2.7 Hz, 1H), 7.68 (d, *J*=2.5 Hz, 2H), 4.04 (s, 6H), 2.65–2.36 (m, 6H), 1.66–1.36 (m, 38H),−2.72 ppm (s, 2H).

**Synthesis of 3**: A 100 mL Schlenk tube was charged with metalloporphyrin 7 (50 mg, 0.052 mmol) followed by an addition of dry toluene. The solution was degassed by three freeze–pump–thaw cycles. DDQ (0.26 mmol) and Sc(OTf)_3_ (0.26 mmol) were added, and the reaction was heated to 50 °C under argon for 4–6 h. THF was then added, and the reaction was stirred at room temperature for further 1 h. The reaction mixture was then passed through a short plug of alumina with DCM and then DCM/THF (1 : 1) as eluents. The solvent was removed in vacuo, and the residue was purified via silica gel column chromatography by using DCM/THF (90 : 1) solvent mixture as eluant. Removal of solvent under reduced pressure followed by a recrystallization from chloroform/methanol yielded the desired triply fused dimer Yield: 38 mg (76 %). ESI‐MS (M^+^): *m*/*z*=calc. for: C_116_H_118_Zn_2_N_8_O_8_; 1882.7649, found: 1882.7640. ^1^H NMR (250 MHz, chloroform‐d): *δ*=8.19–8.13 (m, 4H), 7.90–7.73 (m, 4H), 7.63 (qd, *J*=4.6, 1.3 Hz, 4H), 7.57–7.52 (m, 4H), 7.49 (d, *J*=2.6 Hz, 4H), 7.44 (t, *J*=2.6 Hz, 2H), 7.39 (t, *J*=2.2 Hz, 2H), 7.36–7.32 (m, 2H), 7.30 (d, *J*=5.0 Hz, 2H), 3.95 (s, 6H), 3.18–2.90 (m, 12H), 1.46–1.28 ppm (m, 72H).

**Synthesis of 2**: For Cu^II^ metallation, a solution of Cu(OAc)_2_ (14 mg, 0.073 mmol) in MeOH was added to a solution of **6** (50 mg, 0.049 mmol) in CHCl_3_, and the resulting mixture was stirred for 1 h. After the complete metalation was confirmed by TLC, the mixture was poured into water, and the porphyrin products were extracted with CHCl_3_. The organic layer was separated, and the combined extracts were washed with water and brine and dried over anhydrous Na_2_SO_4_. The solvent was removed, and the residue was purified by silica gel column chromatography. The desired red colored pure product was eluted by DCM/hexane (1 : 1) solvent mixture followed by a recrystallization from chloroform/methanol yielded pure **2**. Yield: 45 mg (84 %). ESI‐MS: *m*/*z*=calc. for: C_66_H_68_CuN_4_O_6_; 1076.4508; found: 1076.4503. UV/Vis: *λ*_max_=417, 536 nm.

**Synthesis of 1**: Fused zinc porphyrin dimer **3** (50 mg,0.026 mmol) was dissolved in DCM (10 mL), conc. hydrochloric acid (0.2 mL) was added, and the reaction mixture was vigorously stirred for 15 min. Reaction mixture was then quenched with water and extracted with mixture of DCM and saturated NaHCO_3_ three times. The organic layer was then collected, and solvent was removed in vacuum. The product obtained was used without further purification for metalation. For Cu^II^ metallation, a saturated solution of Cu(OAc)_2_ in MeOH was added to a solution of free‐base fused porphyrin in CHCl_3_, and the resulting mixture was refluxed for overnight. After the complete metallation was confirmed by TLC, the mixture was poured into water, and the porphyrin products were extracted with CHCl_3_. The organic layer was separated, and the combined extracts were washed with water, brine solution, and dried over anhydrous Na_2_SO_4_. The solvent was removed, and the residue was purified via silica gel column chromatography. Purification on a silica gel (60 M) column by using DCM/hexane (7 : 3) solvent mixture as the eluent resulted in the isolation of violet colored pure compound. Removal of solvent followed by a crystallization from the mixture of DCM and methanol yielded pure complex **1**. Yield: 39 mg (79 %). ESI‐MS: *m*/*z*=calc. for: C_116_H_118_Cu_2_N_8_O_8_; 1876.7659 found: 1876.7662. UV/Vis (DCM): *λ*_max_=414, 576, 887, 986 nm.

## Conflict of interest

The authors declare no conflicts of interests

1

## Supporting information

As a service to our authors and readers, this journal provides supporting information supplied by the authors. Such materials are peer reviewed and may be re‐organized for online delivery, but are not copy‐edited or typeset. Technical support issues arising from supporting information (other than missing files) should be addressed to the authors.

Supporting Information

## Data Availability

The data that support the findings of this study are available in the supplementary material of this article.
