# Supplementary material for: Remarkable Enhancement of Catalytic Activity of Cu‐Complexes in the Electrochemical Hydrogen Evolution Reaction by Using Triply Fused Porphyrin
Source: ChemSusChem. 2022 Dec 14;16(1):e202201146. doi: 10.1002/cssc.202201146 (PMC10107348; doi:10.1002/cssc.202201146)
Supplement: Supplementary file 1 — Supporting Information [file CSSC-16-0-s001.pdf]

ChemSusChem

Supporting Information

Remarkable Enhancement of Catalytic Activity of Cu-Complexes in the Electrochemical Hydrogen Evolution Reaction by Using Triply Fused Porphyrin**

Shubhadeep Chandra[+], Arijit Singha Hazari[+], Qian Song, David Hunger, Nicolás. I. Neuman, Joris van Slageren,* Elias Klemm,* and Biprajit Sarkar*© 2022 The Authors. ChemSusChem published by Wiley-VCH GmbH. This is an open access article under the terms of the Creative Commons Attribution License, which permits use, distribution and reproduction in any medium, provided the original work is properly cited.

Remarkable Enhancement of Catalytic Activity of Cu-Complexes in the Electrochemical Hydrogen Evolution Reaction (HER) by Using Triply-Fused Porphyrin

Shubhadeep Chandra, † Arijit Singha Hazari, † Qian Song, David Hunger, Nicolás. I. Neuman, Joris van Slageren, * Elias Klemm, * and Biprajit Sarkar*

[a] S. Chandra, Dr. A. S. Hazari, Dr. N. I. Neuman, Prof. Dr. B. Sarkar
Lehrstuhl für Anorganische Koordinationschemie, Institut für Anorganische Chemie
Universität Stuttgart
Pfaffenwaldring 55, D-70569, Stuttgart, Germany
E-mail: biprajit.sarkar@iac.uni-stuttgart.de
[b] Dr. Q. Song, E. Klemm
Institute of Chemical Technology,University of Stuttgart
Pfaffenwaldring 55, 70569, Stuttgart, Germany
E-mail: elias.klemm@itc.uni-stuttgart.de
[c] D. Hunger, J. van Slageren
Institut für Physikalische Chemie,Universität Stuttgart
Pfaffenwaldring 55, 70569 Stuttgart, Germany
E-mail: slageren@ipc.uni-stuttgart.de
[d] Dr. N. I. Neuman
Instituto de Desarrollo Tecnológico para la Industria Quĺmica CCT
INTEC, UNL-CONICET, Predio CONICET Santa Fe
Dr. Alberto Cassano, Ruta Nacional N° 168, Km 0, Paraje El Pozo, (S3000ZAA) Santa Fe, Argentina
† S.C and A.S.H contributed equally to this work

General experimental considerations and instrumentation:

Unless otherwise stated, all experiments were carried out under an argon atmosphere using standard Schlenk techniques or in a MBraun Unilab SP GloveBox. Pyrrole was obtained from Sigma Aldrich and distilled prior to use. Commercially available chemicals like 3,5-Di-tert-butyl-2-methoxybenzaldehyde (abcr GmbH), $Sc(OTf)_3$(Sigma Aldrich), 2,3-Dichloro-5,6-dicyano-1,4-benzoquinone (abcr GmbH), $Cu(OAc)_2.4H_2O$ (Sigma Aldrich) were used without further purification. KC_8 were synthesized according to literature procedure.[1] Dry DMF was purchased from Sigma Aldrich (99.8% extra dry) and was used as received. Other solvents were purified from the MBRAUN MB-SPS-800 solvent system. All of the solvents were degassed by standard techniques prior to use. Column chromatography was conducted using silica from MachereyNagel (Silica 60, 0.04−0.063 mm). ^{1}H NMR spectra were recorded on NMR spectra were acquired on Bruker Avance 250 MHz and Bruker Avance 400 MHz spectrometers at 20 ºC. Chemical shifts are reported in parts per million (relative to the tetramethylsilane signal) with reference to the residual solvent peaks.[2] Mass spectrometry was performed on Bruker Daltonics Microtof-Q (Electron spray ionization (ESI) at 0.4 bar, 200° C, dry gas flow 4.0 l/min with a set capillary of 4500 V, end plate offset -450 V in positive mode and at 1.0 bar, set capillary 2200 V in negative mode) or Varian MAT 711 (EI at 70 eV) mass spectrometers. UV−Vis−NIR spectra were recorded with an on a J&M TIDAS spectrometer instrument.

Electrochemistry:

Cyclic voltammograms were recorded with a PalmSens4 potentiostat or with a Metrohm Autolab PGSTAT101 by working in anhydrous and degassed DMF (99.8% extra dry, Sigma Aldrich) with 0.1 M $^nBu_4PF_6$ (dried, >99.0%, electrochemical grade, Fluka) as the supporting electrolyte. Concentrations of the complexes were about 1×10^{-4} M. A three-electrode setup was used with a glassy carbon working electrode, a coiled platinum wire as counter electrode, and a coiled silver wire as a pseudoreference electrode. The ferrocene/ ferrocenium couple was used as an internal reference. Electrochemical proton reduction experiments were carried out in distilled and degassed DMF solution of 1×10^{-4} M catalysts with 0.1 M $^nBu_4PF_6$ as supporting electrolyte and varying amounts of TFA. The experiments were carried out with 3-mm diameter galssy carbon working electrode which was polished with 0.3 μm alumina slurry prior to each scan. While, coiled platinum wire and coiled silver wire were used as counter and pseudoreference electrodes respectively. All the measurements in electrochemical HER were referenced against ferrocene/ ferrocenium redox couple.

Bulk electrolysis measurements:

Bulk electrolysis measurements were performed in a two-compartment cell divided by microporous membrane (Celgard® 2325). A 0.06 mM DMF solution of **1** or 0.12 mM of **2** containing 0.1 M $^{n}Bu_4PF_6$ and 0.1 M of TFA was sparged with N_2 before measurements. The measurements were performed with Origalys (OGF500) using a carbon paper (1*1cm^2 as catalytic area) as a working electrode, platinum foil as a counter electrode and a coiled silver wire as a pseudoreference electrode. Samples of the headspace (4 mL) were taken using a gastight syringe. The headspace composition was analyzed using a gas chromatograph equipped with molecular-sieve columns and dual TCD and FID detectors using helium as a carrier gas.

Control experiments:

Reduction by $CoCp_2$: The reduction of **1** by $CoCp_2$ was monitored via UV-Vis-NIR spectroscopy at room temperature. One electron reduced species, $\mathbf{1}^{-}$ was generated by a treatment of 10 mL solution of **1** (20 mg (0.011 mmol) in dry CH_2Cl_2 with one equivalent of $CoCp_2$ (2.07 mg, 0.011 mmol) under inert atmosphere. The reaction was allowed to stir for 30 min followed by removal of a solvent under reduced pressure. The residue was dissolved again in dry CH_2Cl_2 and UV-Vis-NIR spectra of the species was recorded immediately. The resulting solution was further treated with excess TFA and UV-Vis-NIR spectra of the resulting species was also recorded.

Reduction by KC_8: Two electron reduced species, $\mathbf{1}^{2-}$ was generated by treating 10 mL solution of **1** (20 mg (0.011 mmol) in dry CH_2Cl_2 with 2 equivalents of KC_8 under inert reaction conditions. The color of the solution changed immediately from violet to deep green and the reaction was allowed to stir for another 10 minutes at room temperature. The solvent was then removed under reduced pressure followed by a filtration to remove unreacted KC_8. UV-Vis-NIR spectra of the resulting solution was then recorded immediately. Further reaction of the doubly reduced species with excess TFA was also monitored by UV-Vis-NIR spectroscopy.

Spectroelectrochemistry:

Spectro-electrochemical measurements were carried out in an optically transparent thin-layer electrochemical (OTTLE)[3] cell (CaF2 windows) with a platinum mesh working electrode, a platinum-mesh counter electrode, and a silver-foil pseudoreference electrode. Anhydrous and degassed DMF (99.8% extra dry, Sigma Aldrich) with 0.1 M NBu_4PF_6 as the electrolyte was used as the solvent. The catalyst under investigation was dissolved into the 0.1 M NBu_4PF_6 electrolyte solution and the OTTLE cell was then filled with 0.2 mL of the resulting solution and packed under Ar atmosphere. UV-Vis-NIR spectra of the *in-situ* generated species via

application of desired potential corresponding to the specified redox processes were recorded simultaneously under specified time interval.

Electron Paramagnetic Resonance:

Electron Paramagnetic Resonance. EPR spectra at X-band frequency (ca. 9.5 GHz) were obtained with a Magnettech MS-5000 benchtop EPR spectrometer equipped with a rectangular TE 102 cavity and TC HO4 temperature controller. The measurements were performed in synthetic quartz glass tubes. Spectral simulations were performed using the EasySpin package[4] running in Matlab R2018b.

Density Functional Theory:

All calculations were performed with the ORCA program package, versions 4.0.1.2 and 4.2.8 [5] The geometries of all species were optimized using the PBE0 functional, [6] the def2-SVP basis sets on all atoms except for Cu, for which the def2-TZVP basis set was used.[7] Solvation was taken into account using the using the SMD method together with the CPCM model[8] using DMF as solvent, and dispersion corrections were included using the D3 dispersion correction model.[9] The resolution-of-the identity (RI) approximation,[10] with matching basis sets,[11] as well as the RIJCOSX approximation (combination of RI and chain-of-spheres algorithm for exchange integrals) were used to reduce the time of calculations. Numerical frequencies calculations were used in order to check that the optimized structures were local minima and to obtain Gibbs free enthalpies. To obtain more reliable energetics single-point calculations were performed using the optimized geometries, the PBE0 functional and def2-TZVP basis sets on all atoms. Low-lying excitation energies were calculated with time-dependent DFT (TD-DFT). For all calculations spin densities were calculated according to the Löwdin population analysis.[12] Broken-symmetry calculations[13] were carried out using optimized geometry to evaluate the exchange coupling constants. Plots of spin-densities and optimized geometries were performed using Chemcraft.[14]

Overview of synthesis

Step -I:

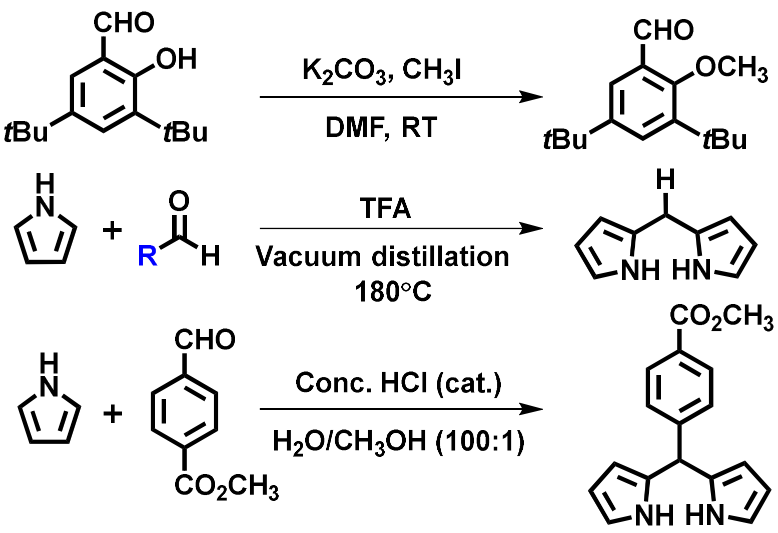

Step -II:

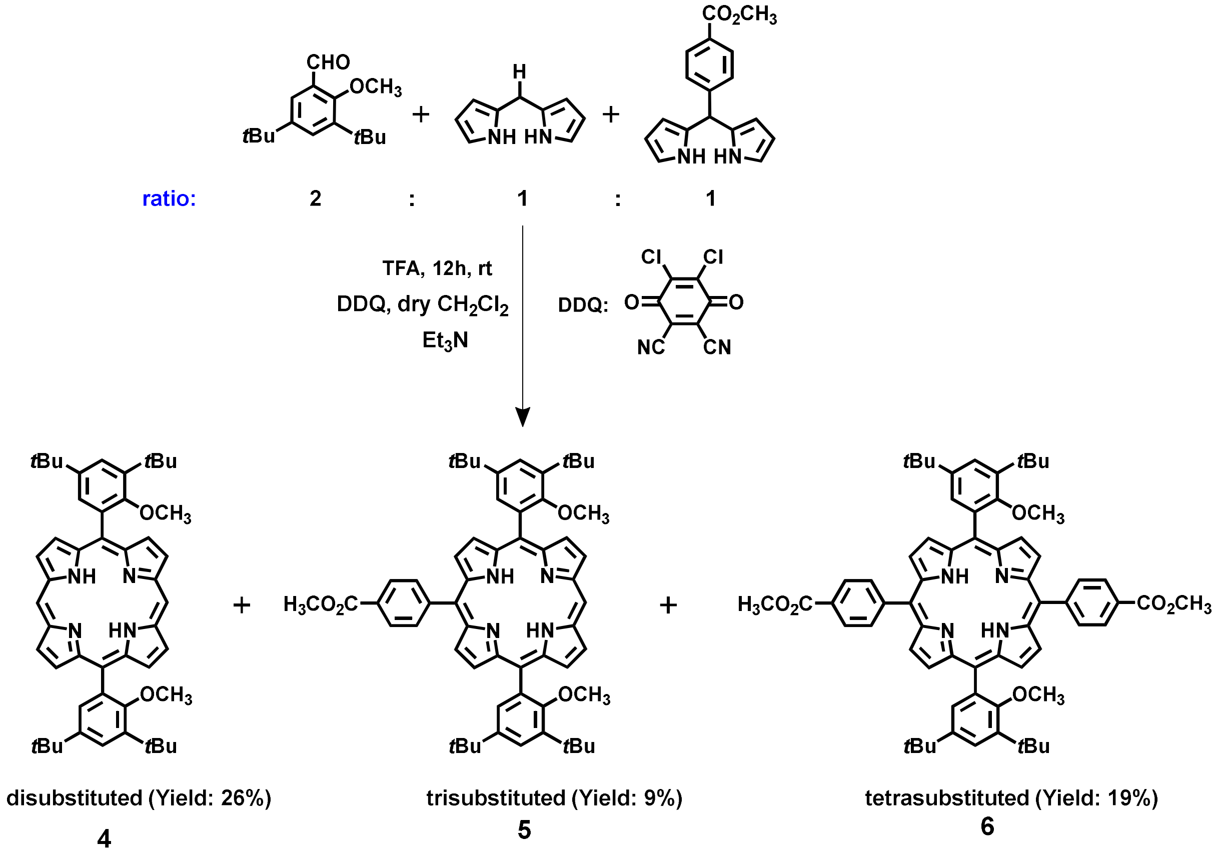

Step-III

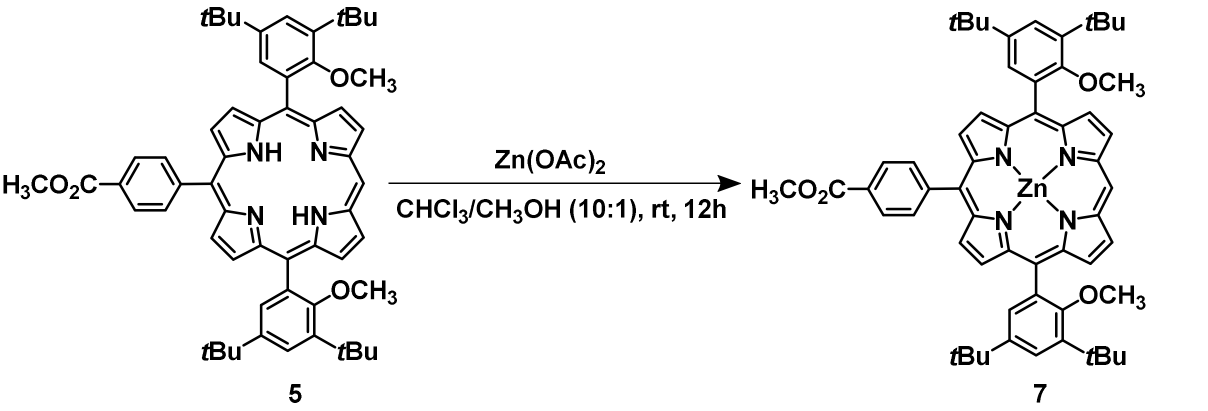

Yield: 95%

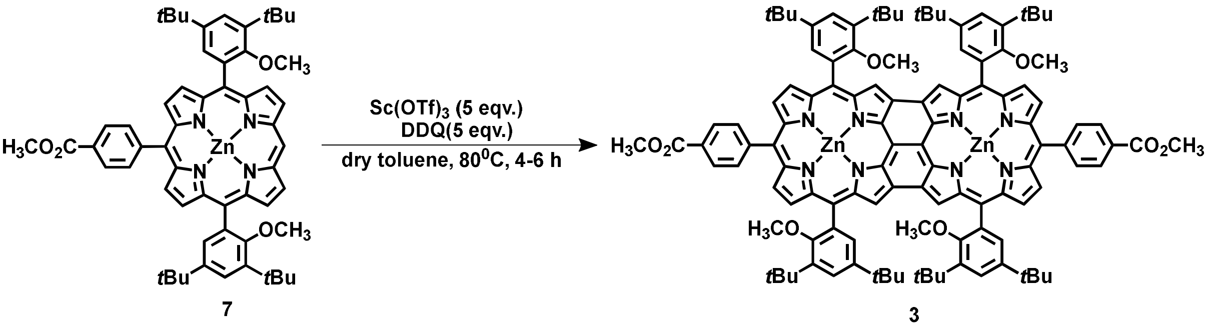

Yield: 76%

Step -IV

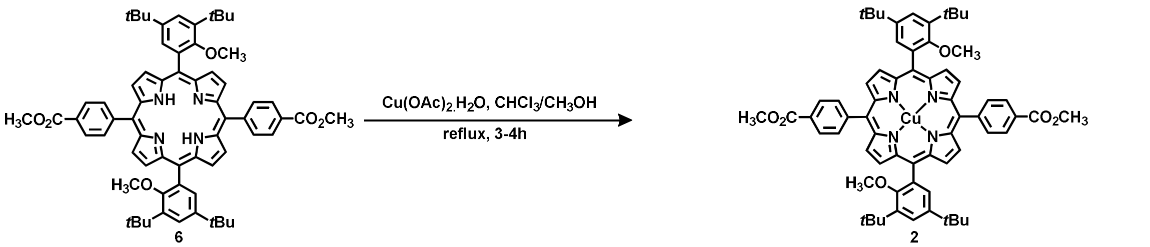

Yield: 84%

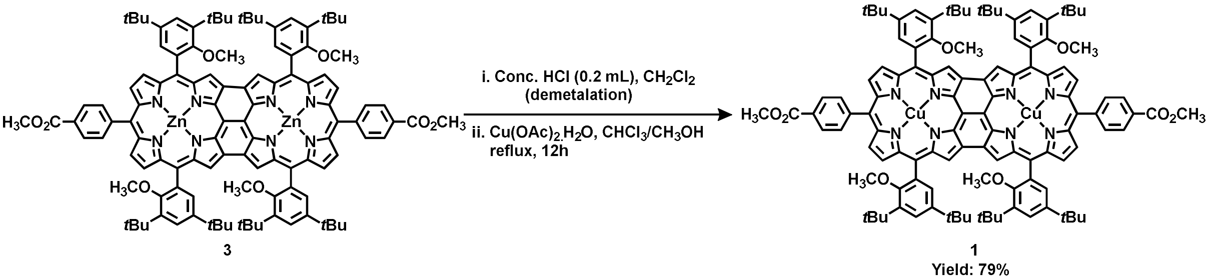

Yield: 79%

Synthetic Procedure:

Methyl 4-formylbenzoate were synthesized following a reported procedure,[15] by reacting 4-formylbenzoic acid (1.0 eq.) with methyl iodide (1.2 eq.) and K_2CO_3 (1.0 eq.) in DMF at 60 ºC for 2-4 hours. The reaction was quenched with H_2O. The crude products were extracted with DCM, the organic phase was washed with H_2O and dried over Na_2SO_4. Chromatographic purification in a small silica column (DCM) was performed to remove yellow impurities. The identity and purity of the product was confirmed by NMR.

3,5-di-tert-butyl-2-methoxybenzaldehyde,[16] Dipyrromethane,[17] and 5-(4-methylcarboxyphenyl)-dipyrromethane[18] was synthesized following the literature reported method.^{1}H-NMR was in complete agreement with the published data.

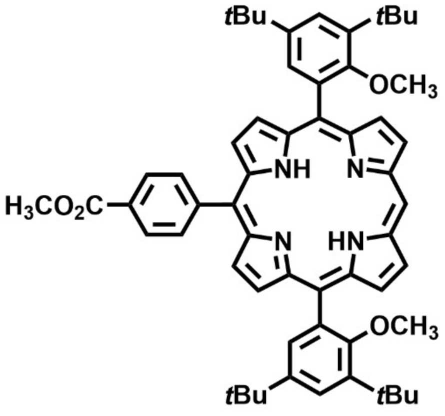

5

3,5-di-tert-butyl-2-methoxy benzaldehyde (1186 mg, 4.78 mmol), 5-(4-methylcarboxyphenyl)-dipyrromethane (669 mg, 2.39 mmol) and dipyromethane (350 mg 2.39 mmol) were dissolved in DCM and degassed for 15 min . TFA (0.38 equiv) was added dropwise and the solution was stirred for overnight in the dark. DDQ (1.6 g, 3 equiv) was added and further stirred for three hours. The reaction was quenched with triethylamine and solvent was removed under reduced pressure. The crude product was purified by silica gel column chromatography and the desired product was eluted by using DCM/Hexane (1:1) solvent mixture. Second fraction was collected and removal of solvent yielded reddish-pink colored pure compund **5**.

[**5**]: Yield: 189 mg (9%), ESI-MS ($M+H^+$): m/z = calc. for: $C_{58}H_{65}N_4O_4$; 881.5000, found: 881.4987, ^{1}H NMR (250 MHz, Chloroform-d) δ 10.20 (s, 1H), 9.34 (d, J = 4.6 Hz, 2H), 9.11 (d, J = 4.6 Hz, 2H), 9.00 (d, J = 4.7 Hz, 2H), 8.80 (d, J = 4.8 Hz, 2H), 8.43 (d, J = 8.1 Hz, 2H), 8.32 (d, J = 8.0 Hz, 2H), 7.86 (d, J = 2.4 Hz, 2H), 7.77 (d, J = 2.5 Hz, 2H), 4.11 (s, 3H), 2.58 (s, 6H), 1.68 (d, J = 6.5 Hz, 18H), 1.47 (p, J = 2.6 Hz, 18H).

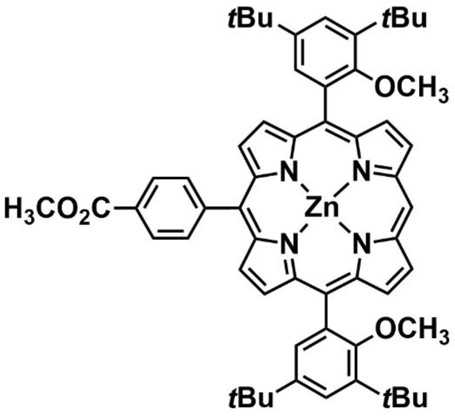

7

For Zn(II) metallation, a solution of $Zn(OAc)_2.2H_2O$ (49 mg, 0.226 mmol) in MeOH was added to a solution of **5** (100 mg, 0.113 mmol) in $CHCl_3$, and the resulting mixture was stirred for 1 h. The progress of the reaction was monitored by TLC. After complete conversion was achieved, the mixture was poured into water, and was extracted with $CHCl_3$. The organic layer was separated, and the combined extracts were repeatedly washed with water and brine and dried over anhydrous Na_2SO_4. The solvent was removed and the residue was washed with MeOH to provide pure material **7** that can be used without further purification.

[**7**]: Yield: 106 mg (99%), ESI-MS ($M+H^+$): m/z = calc. for: $C_{58}H_{63}N_4O_4Zn$; 943.4135, found: 943.4131, ^1H NMR (250 MHz, Chloroform-d) δ 10.25 (s, 1H), 9.42 (d, J = 4.4 Hz, 2H), 9.20 (dd, J = 4.5, 2.0 Hz, 2H), 9.09 (dd, J = 4.7, 1.5 Hz, 2H), 8.90 (d, J = 4.7 Hz, 2H), 8.46 – 8.16 (m, 4H), 7.93 (dd, J = 15.3, 2.4 Hz, 2H), 7.81 – 7.73 (m, 2H), 4.10 (s, 3H), 2.50 (d, J = 3.0 Hz, 6H), 1.66 (s, 18H), 1.48 (dd, J = 3.5, 1.1 Hz, 18H).

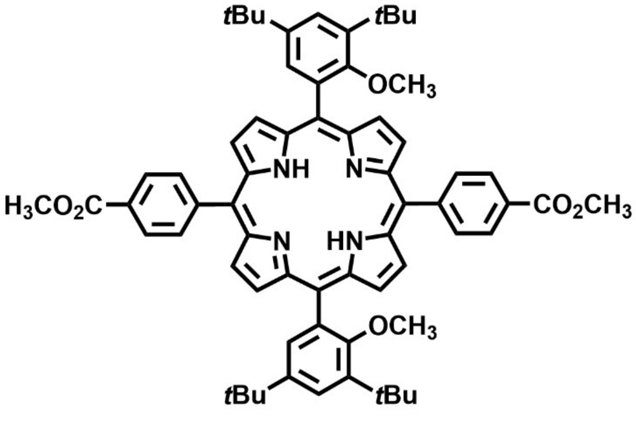

6

3,5-di-tert-butyl-2-methoxy benzaldehyde (1186 mg, 4.78 mmol),5-(4-methylcarboxyphenyl)-dipyrromethane (669 mg , 2.39 mmol) and dipyromethane (350 mg 2.39 mmol) were dissolved in DCM and degassed for 15 min . TFA (0.38 equiv) was added dropwise and the solution was stirred for overnight in the dark. DDQ (1.6 g, 3 equiv) was added and the reaction mixture was allowed to stir for three hours. The reaction was quenched with triethylamine and the solvent was removed under reduced pressure. The crude product was purified via silica gel column chromatography. The desired product was eluted with DCM/Hexane solvent mixture (4:1 to pure DCM). Third fraction was collected and solvent was removed under reduced pressure. Crystallization of the compound from chloroform/methanol mixture resulted in red colored pure compound **6.**

[**6**]: Yield: 218 mg (19%), ESI-MS ($M+H^+$): m/z = calc. for: $C_{66}H_{71}N_4O_6$; 1015.5368, found: 1015.5351, ^{1}H NMR (250 MHz, Chloroform-d) δ 8.89 (d, J = 4.6 Hz, 3H), 8.72 (d, J = 5.5 Hz, 4H), 8.43 – 8.13 (m, 8H), 7.85 (d, J = 2.4 Hz, 1H), 7.80 (d, J = 2.3 Hz, 1H), 7.74 (d, J = 2.7 Hz, 1H), 7.68 (d, J = 2.5 Hz, 2H), 4.04 (s, 6H), 2.65 – 2.36 (m, 6H), 1.66 – 1.36 (m, 38H). -2.72 (s, 2H).

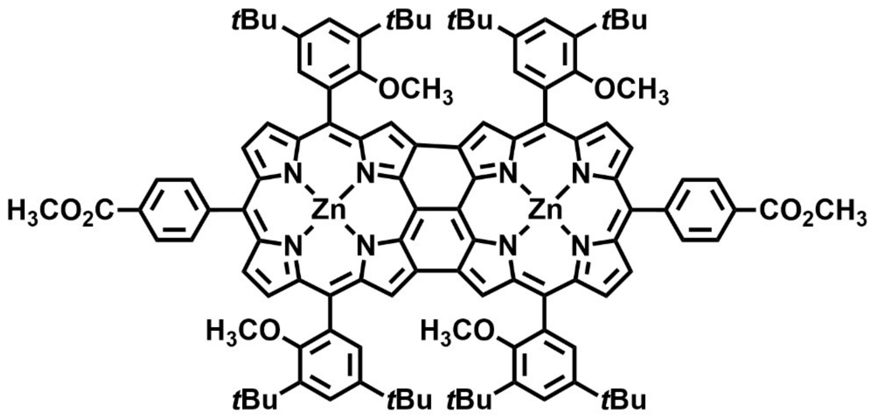

3

A 100 mL Schlenk tube was charged with metalloporphyrin **7** (50 mg, 0.052 mmol) followed by an addition of dry toluene. The solution was degassed by three freeze–pump–thaw cycles. DDQ (0.26 mmol) and $Sc(OTf)_3$ (0.26 mmol) were added, and the reaction was heated to 50 °C under argon for 4-6 h. THF was then added and the reaction was stirred at room temperature for further 1 h. The reaction mixture was then passed through a short plug of alumina with CH_2Cl_2 and then CH_2Cl_2: THF (1:1) as eluents. The solvent was removed in vacuo, and the residue was purified via silica gel column chromatography by using DCM/THF (90:1) solvent mixture as a eluant. Removal of solvent under reduced pressure followed by a recrystallization from chloroform/methanol yielded the desired triply fused dimer **3**.

[**3**]: Yield: 38 mg (76%), ESI-MS (M^+): m/z = calc. for: $C_{116}H_{118}Zn_2N_8O_8$; 1882.7649, found: 1882.7640, ^1H NMR (250 MHz, Chloroform-d) δ 8.19 – 8.13 (m, 4H), 7.90 – 7.73 (m, 4H), 7.63 (qd, J = 4.6, 1.3 Hz, 4H), 7.57 – 7.52 (m, 4H), 7.49 (d, J = 2.6 Hz, 4H), 7.44 (t, J = 2.6 Hz, 2H), 7.39 (t, J = 2.2 Hz, 2H), 7.36 – 7.32 (m, 2H), 7.30 (d, J = 5.0 Hz, 2H), 3.95 (s, 6H), 3.18 – 2.90 (m, 12H), 1.46 -1.28 (m, 72H).

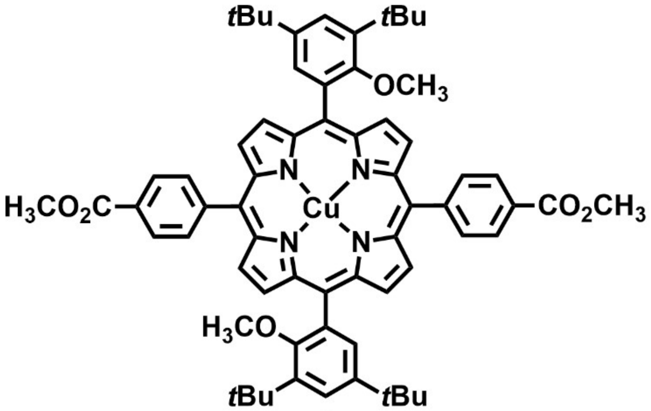

2

For Cu(II) metallation, a solution of $Cu(OAc)_2$ (14 mg, 0.073 mmol) in MeOH was added to a solution of **6** (50 mg, 0.049 mmol) in $CHCl_3$, and the resulting mixture was stirred for 1 h. After the complete metalation was confirmed by TLC, the mixture was poured into water, and the porphyrin products were extracted with $CHCl_3$. The organic layer was separated, and the combined extracts were washed with water and brine and dried over anhydrous Na_2SO_4. The solvent was removed and the residue was purified by silica gel column chromatography. The desired red colored pure product was eluted by DCM/hexane (1:1) solvent mixture followed by a recrystallization from chloroform/methanol yielded pure **2**.

[**2**]: Yield: 45 mg (84%), ESI-MS: m/z = calc. for: $C_{66}H_{68}CuN_4O_6$ 1076.4508; found: 1076.4503, UV-Vis λmax: 417, 536 nm.

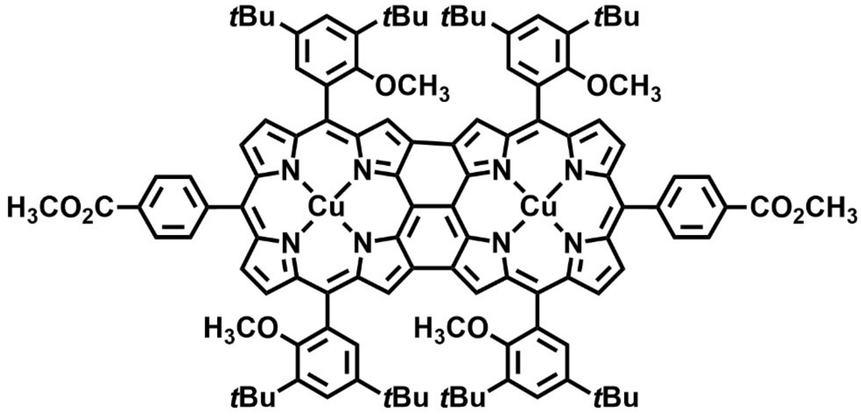

1

Fused zinc porphyrin dimer **3** (50 mg,0.026 mmol) was dissolved in dichloromethane (10 ml), conc. hydrochloric acid (0.2 ml) was added and the reaction mixture was vigorously stirred for 15 min. Reaction mixture was then quenched with water and extracted with mixture of DCM and saturated $NaHCO_3$ three times. The organic layer was then collected and solvent was removed in vacuum. The product obtained was used without further purification for metalation. For Cu(II) metallation, a saturated solution of $Cu(OAc)_2$ in MeOH was added to a solution of free base fused porphyrin in $CHCl_3$, and the resulting mixture was refluxed for overnight. After the complete metallation was confirmed by TLC, the mixture was poured into water, and the porphyrin products were extracted with $CHCl_3$. The organic layer was separated, and the combined extracts were washed with water, brine soluton and dried over anhydrous Na_2SO_4. The solvent was removed and the residue was purified via silica gel column chromatography. Purfification on a silica gel (60M) column by using DCM/hexane (7:3) solvent mixture as the eluent resulted in the isolation of violet colored pure compound. Removal of solvent followed by a crystallization from the mixture of DCM and methanol yielded pure complex **1**.

[**1**]: Yield: 39 mg (79%), ESI-MS: m/z = calc. for: $C_{116}H_{118}Cu_2N_8O_8$; 1876.7659 found: 1876.7662, UV-Vis (DCM) λmax: 414, 576, 887, 986nm.

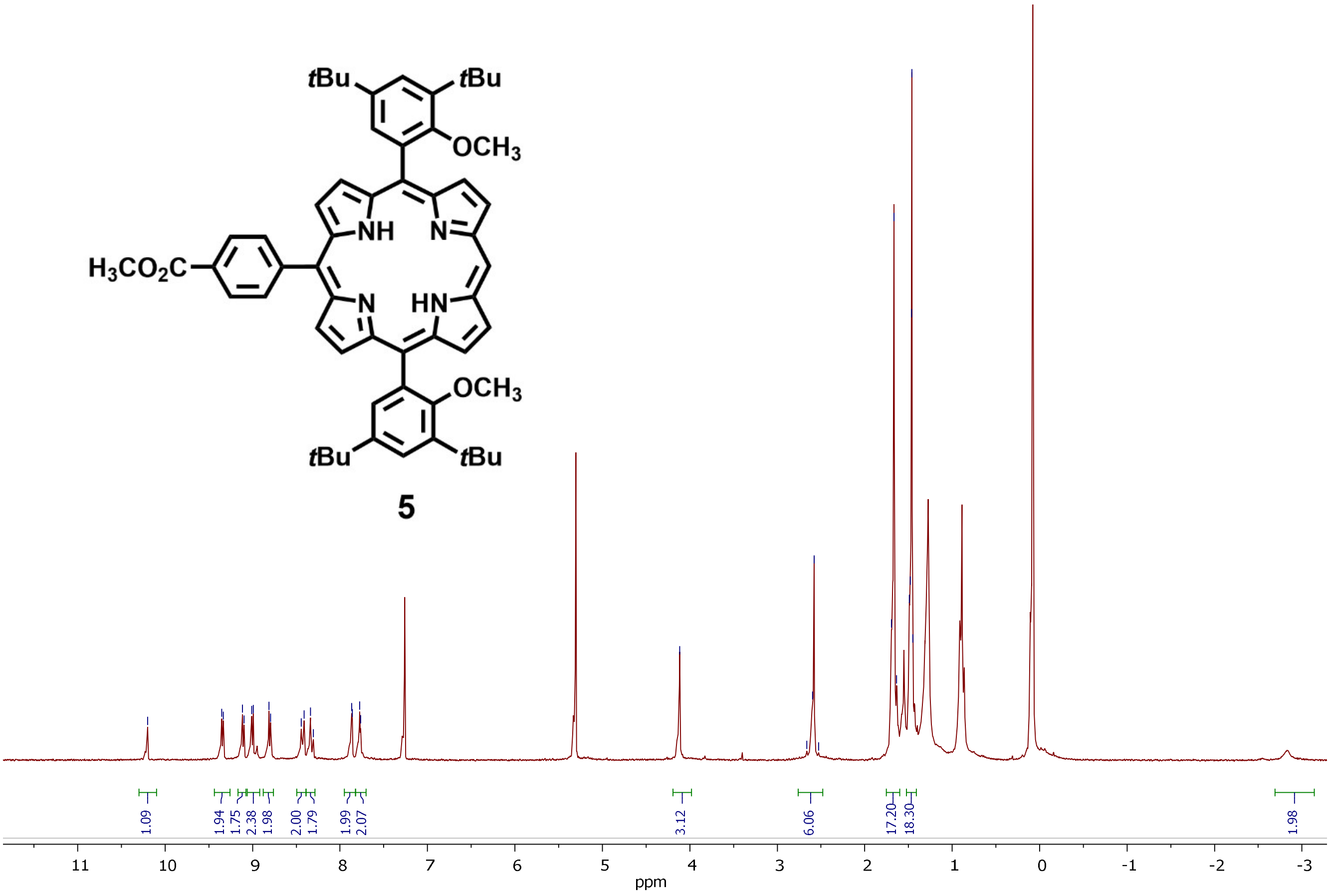

Figure S1. ^{1}H NMR (250 MHz, $CDCl_3$) of porphyrin **5.**

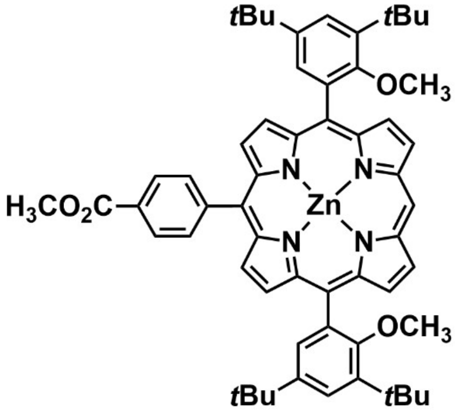

7

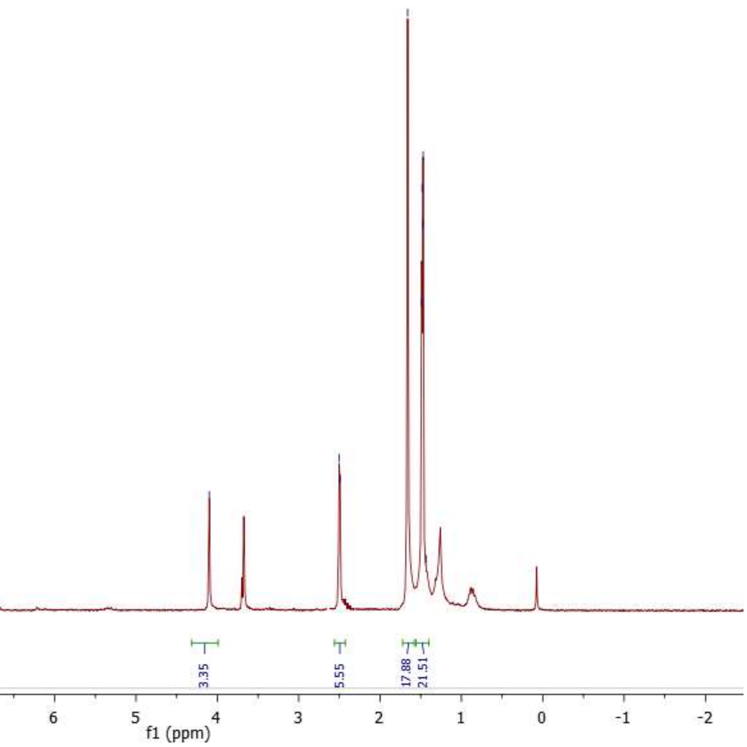

Figure S2. ^{1}H NMR (250 MHz, $CDCl_3$) of porphyrin **7**.

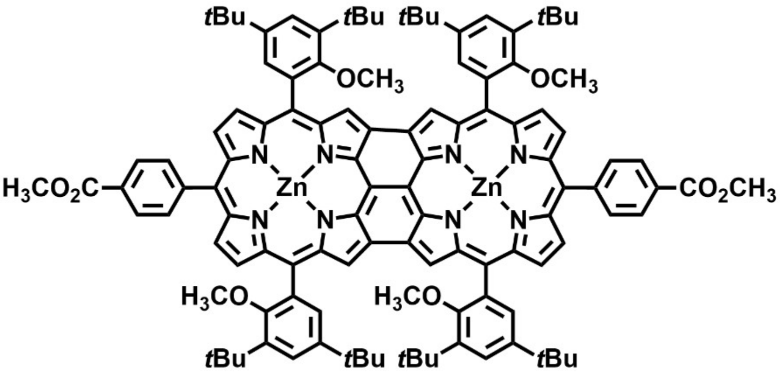

3

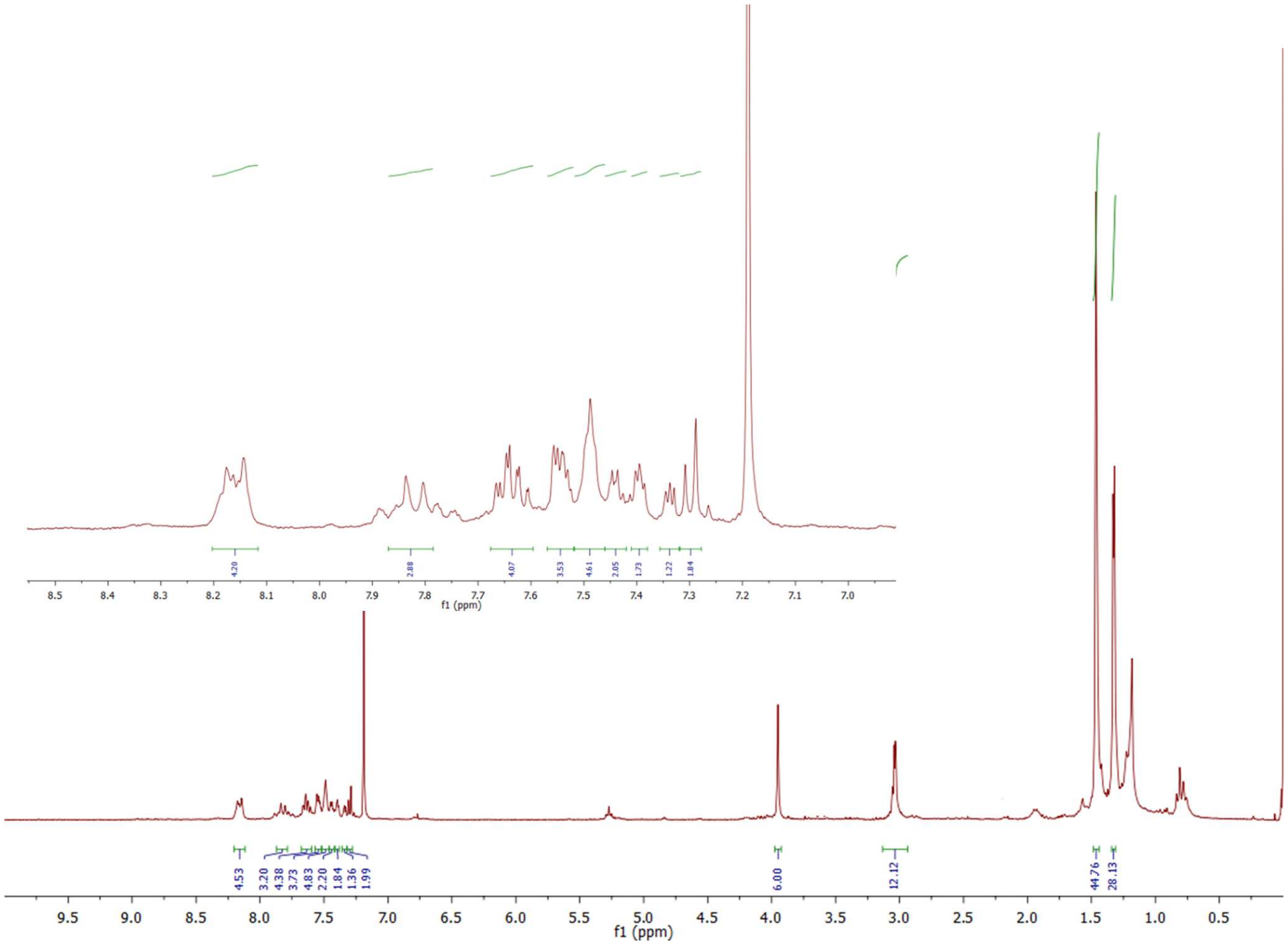

Figure S3. ^{1}H NMR (250 MHz, $CDCl_3$) of porphyrin **3**.

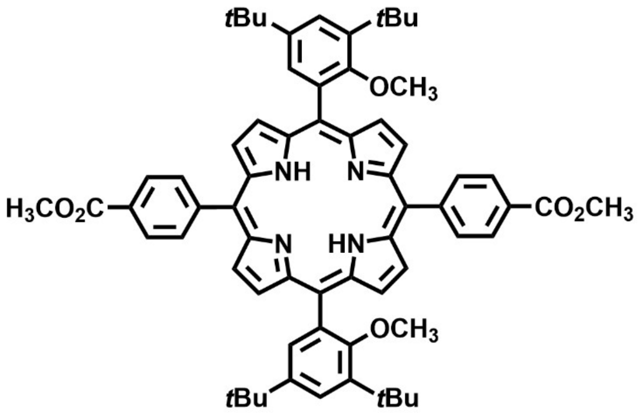

6

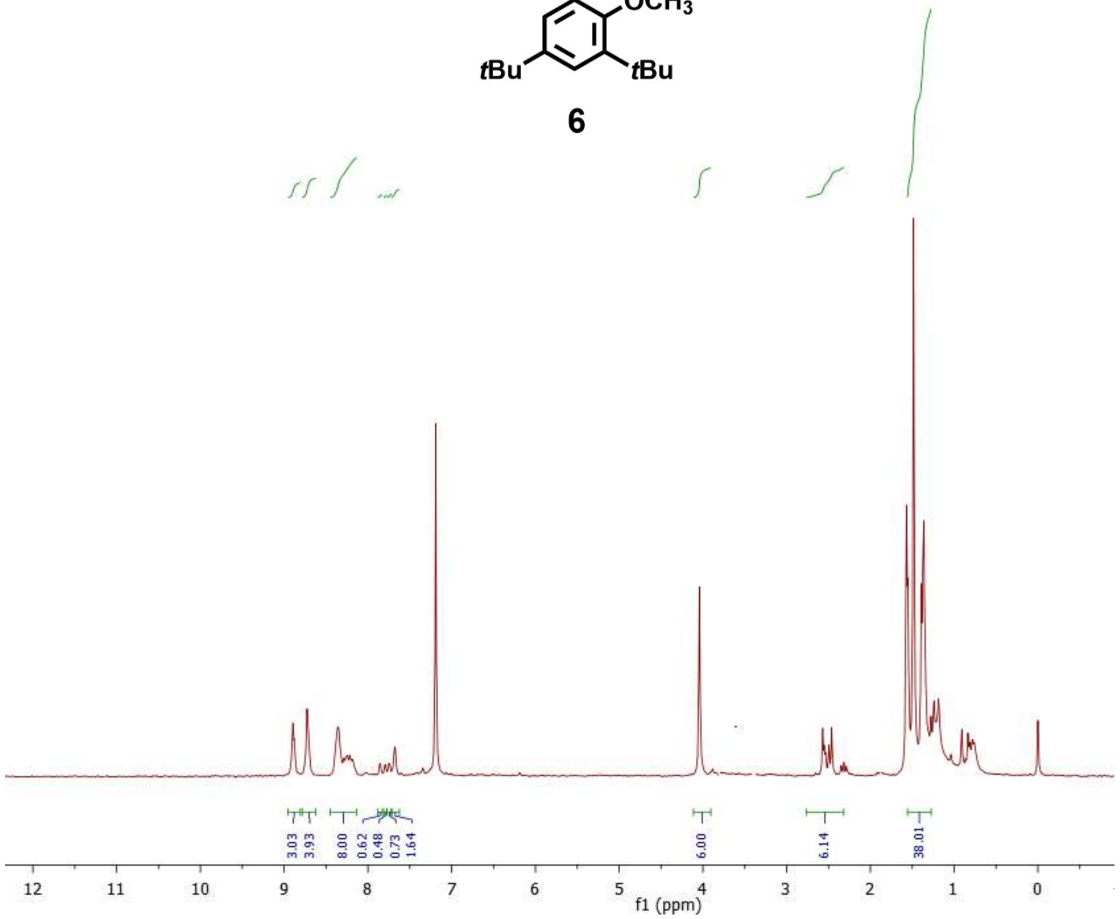

Figure S4. ^{1}H NMR (250 MHz, $CDCl_3$) of porphyrin **6**.

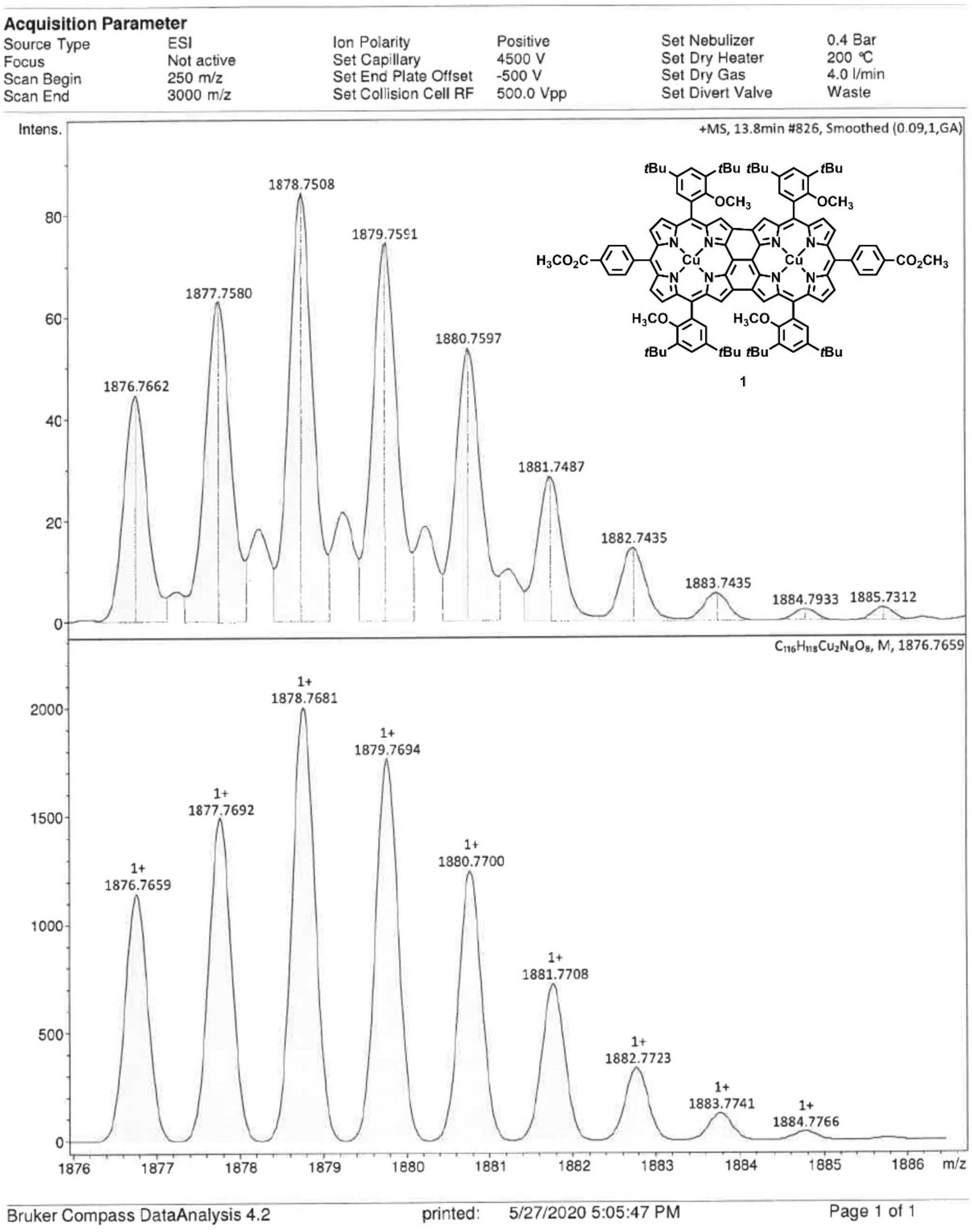

Figure S5. High resolution (ESI-MS) mass spectrum of **1** in dichloromethane.

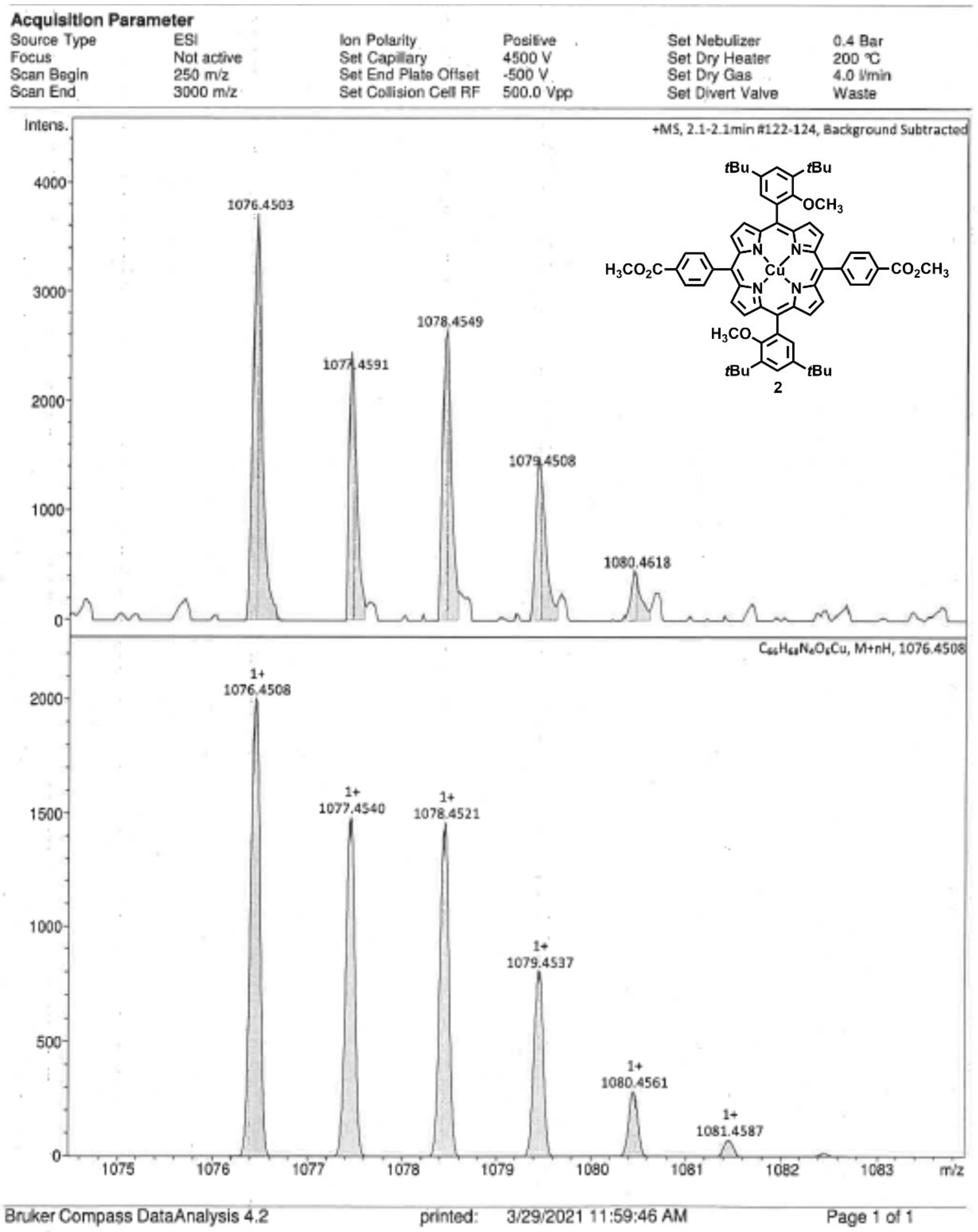

Figure S6. High resolution (ESI-MS) mass spectrum of **2** in dichloromethane.

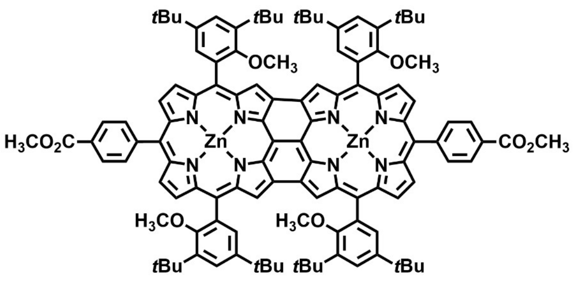

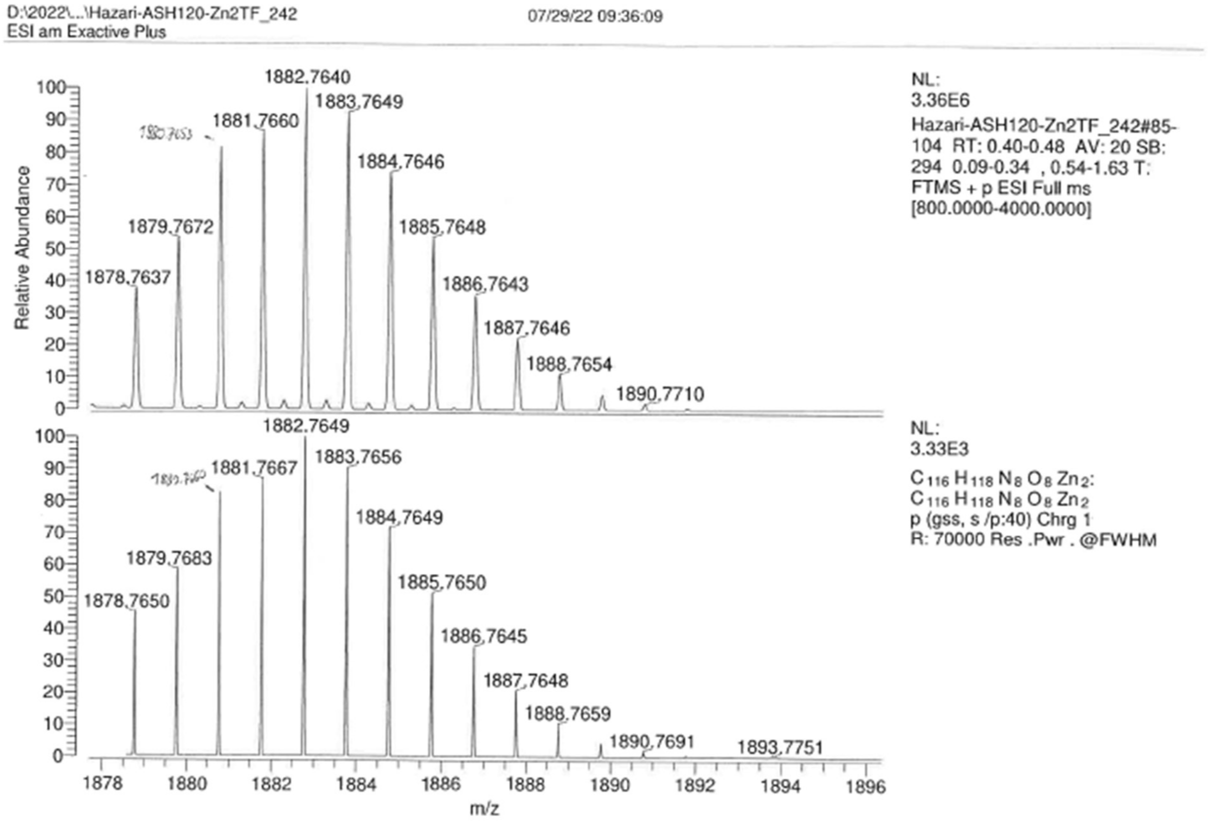

Figure S7. High resolution (ESI-MS) mass spectrum of **3** in dichloromethane.

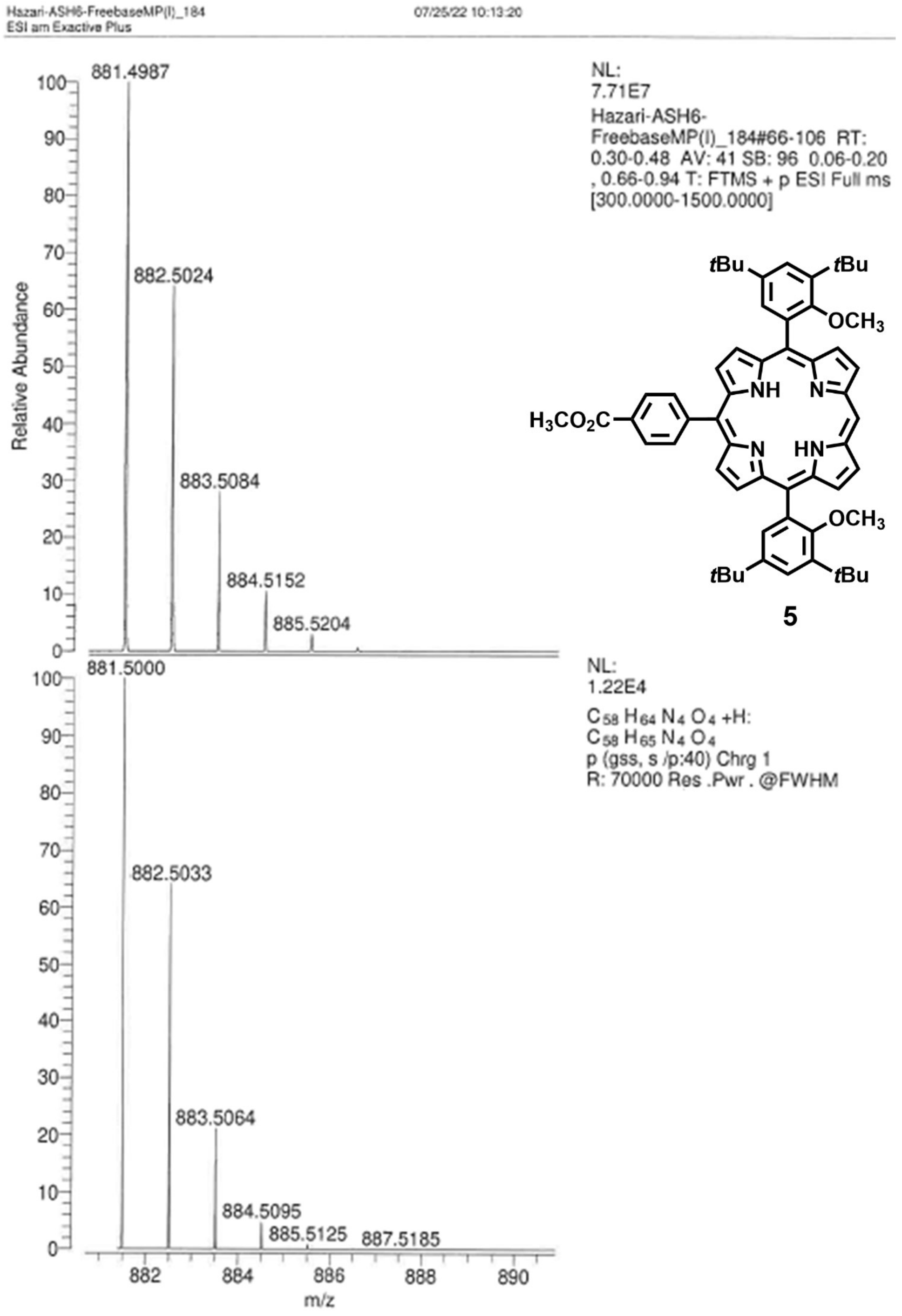

Figure S8. High resolution (ESI-MS) mass spectrum of **5** in dichloromethane.

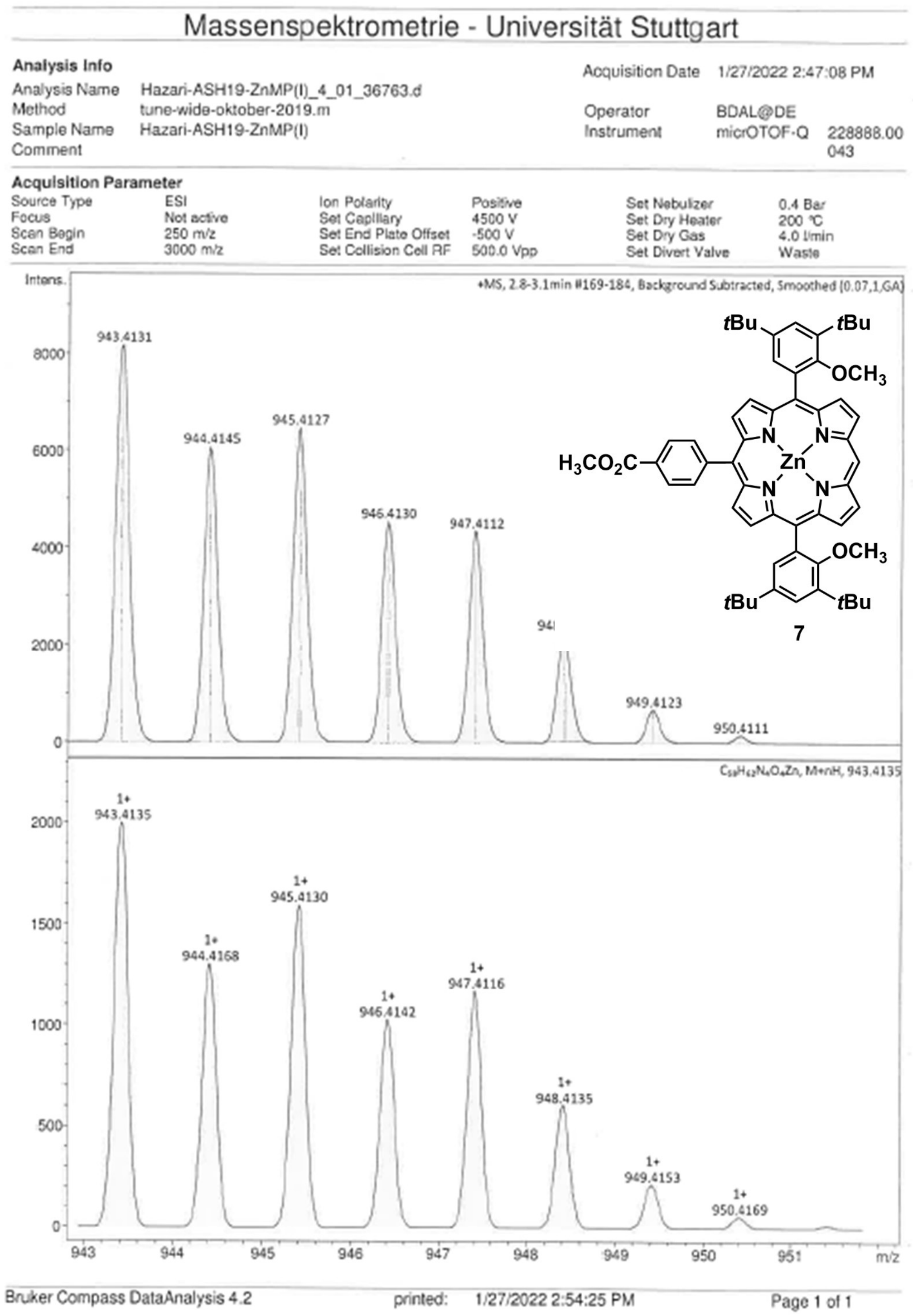

Figure S9. High resolution (ESI-MS) mass spectrum of **7** in dichloromethane.

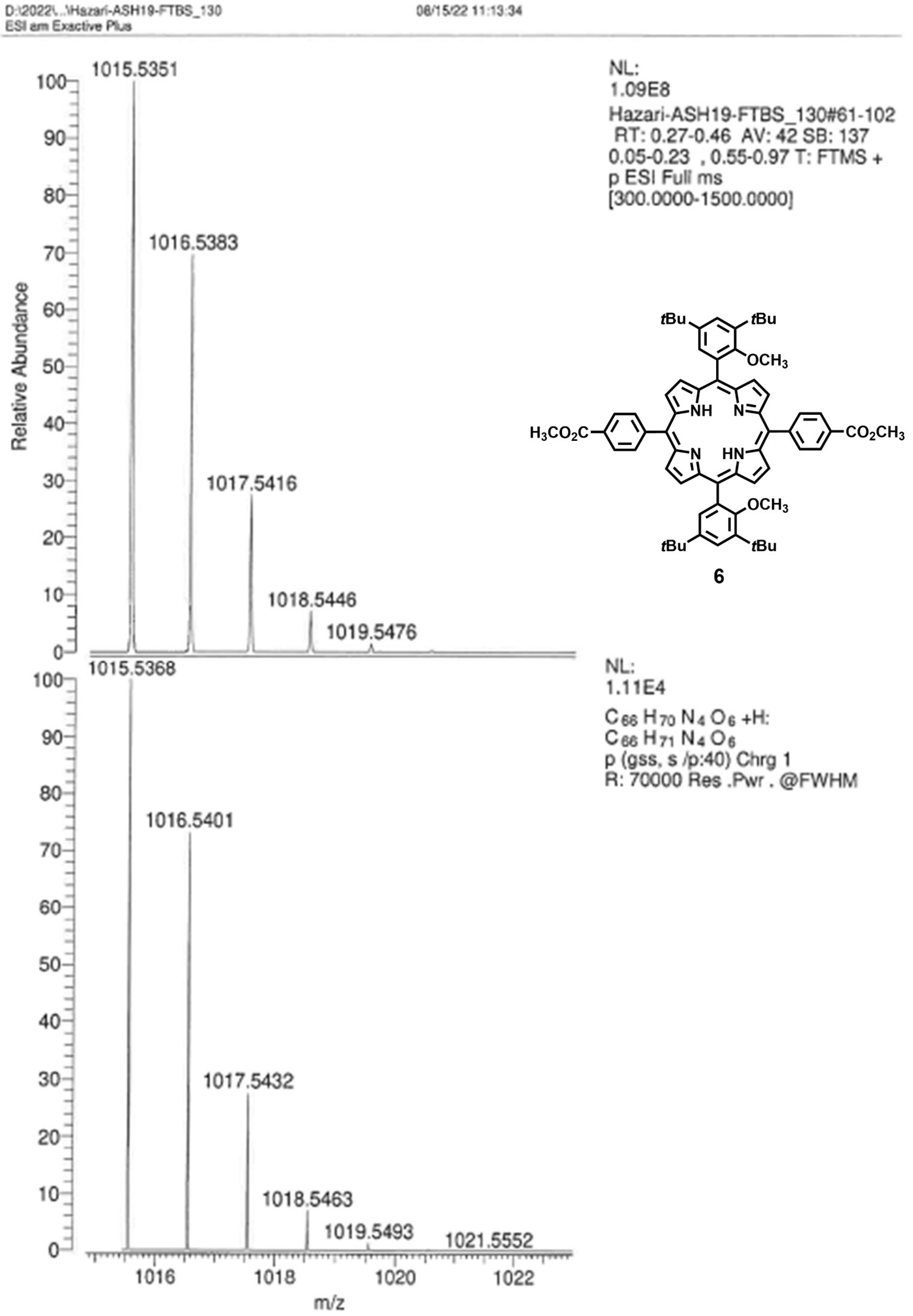

Figure S10. High resolution (ESI-MS) mass spectrum of **6** in dichloromethane.

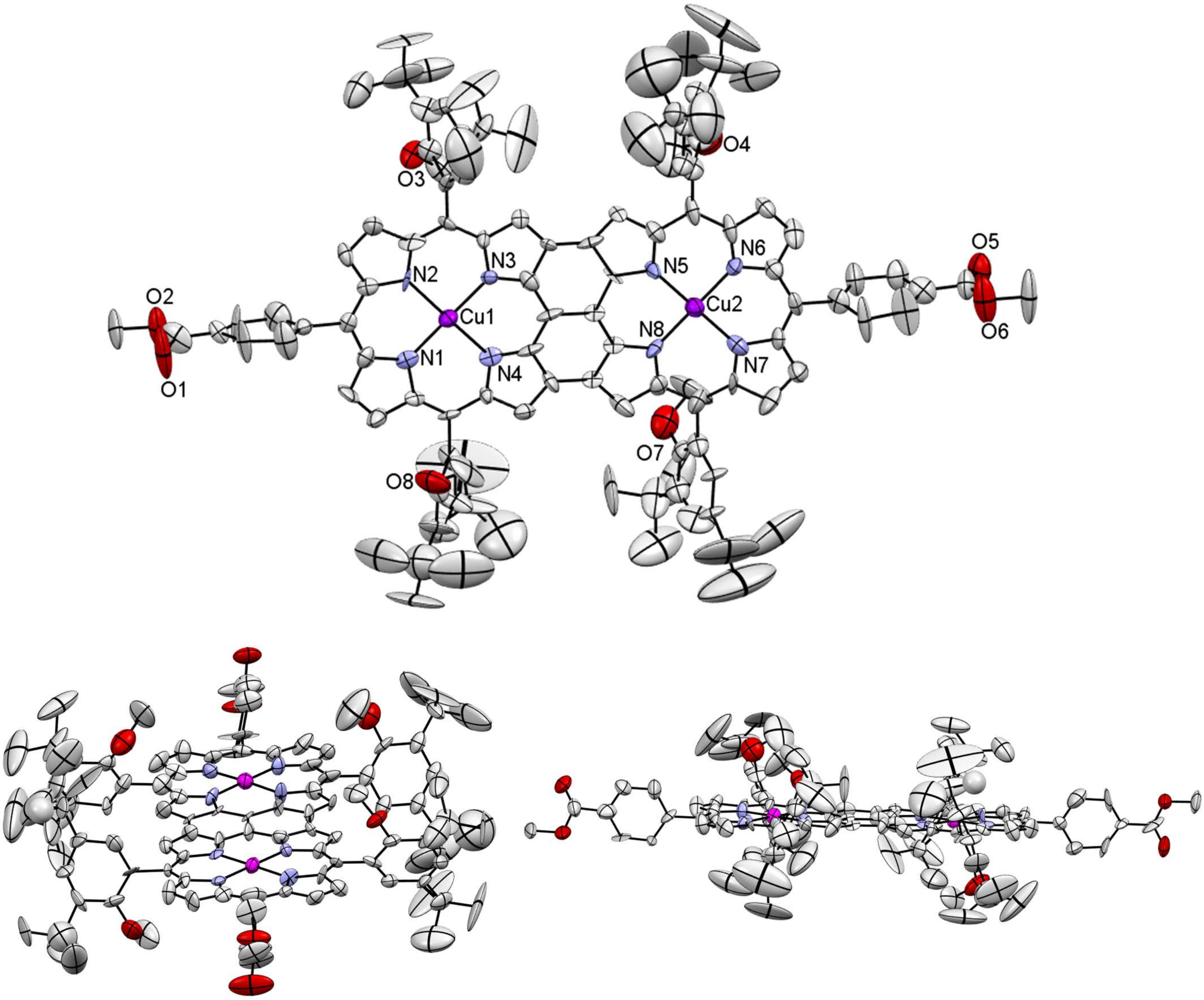

Figure S11. Ortep representation of **1**. Ellipsoids are drawn at 50% probability level. Hydrogen atoms have been omitted for clarity. Diagram below depicts side views of the crystal structure of **1**.

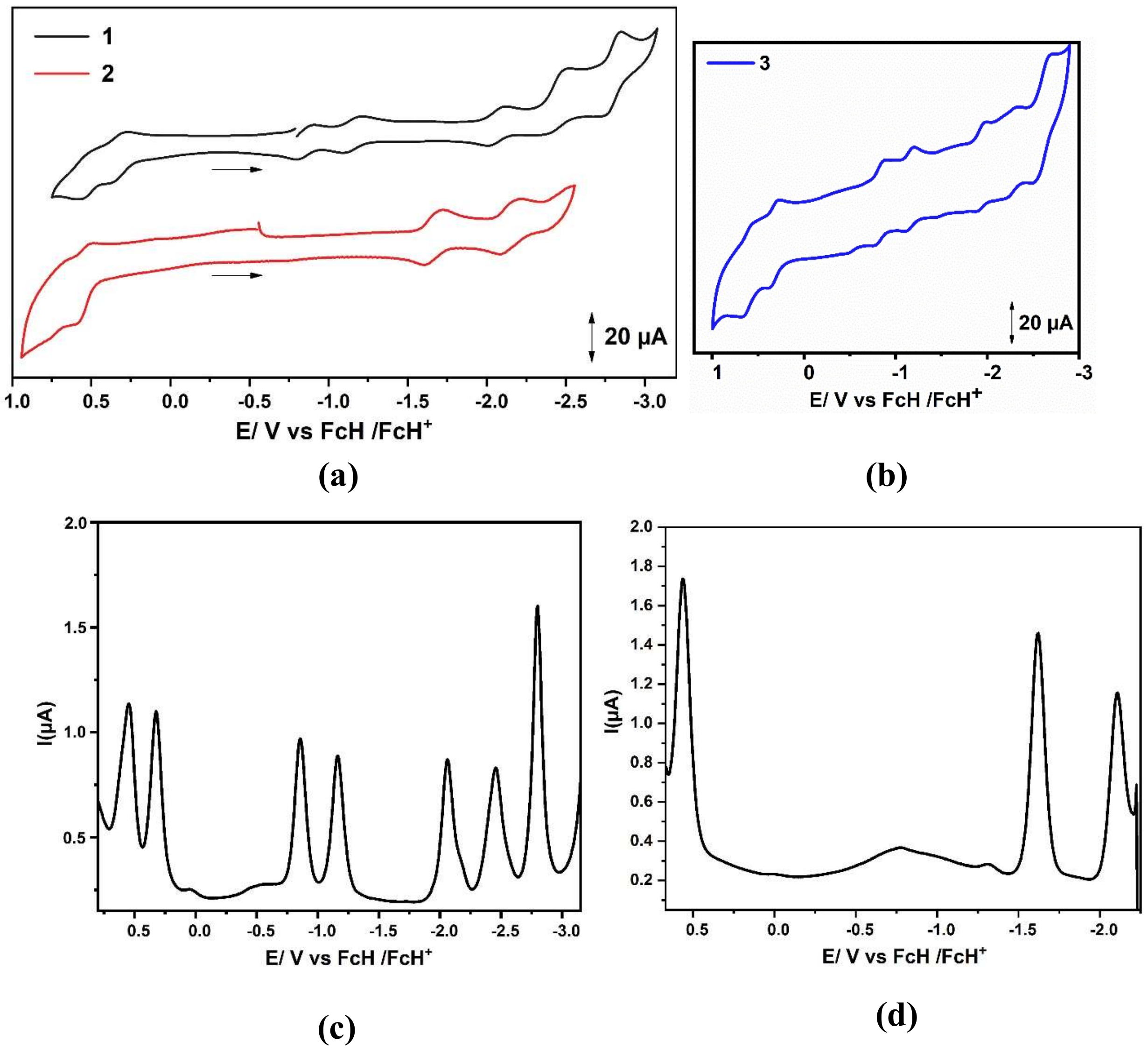

Figure S12. Cyclic voltammograms of 0.25 mM of (a) bimetallic copper fused porphyrin (**1**), copper monomer (**2**), and (b) Zn-fused porphyrin in DMF with 0.1 M $^{n}Bu_4PF_6$ at 100 mV/S. Differential pulse voltammograms of 0.25 mM of (c) bimetallic copper fused porphyrin (**1**), and (d) copper monomer (**2**).

Table S1. Electrochemical Data obtained from Cyclic Voltammetry[a].

Complex	E/[V](ΔE_p/[mV])[b]						
	E_{ox2}	E_{ox1}	E_{red1}	E_{red2}	E_{red3}	E_{red4}	E_{red5}
**1**	0.56 (30)	0.30 (65)	-0.85 (90)	-1.15 (95)	-2.05 (88)	-2.45 (90)	-2.80 (88)
**2**	-	0.56 (60)	-1.65 (91)	-2.12 (86)	-	-	-
**3**	0.62 (80)	0.31 (70)	-0.81 (72)	-1.16 (79)	-1.92 (59)	-2.27 (97)	-2.59 (97)

[a]measured in DMF with 0.1 M $^{n}Bu_4PF_6$ at 100 mV/S. [b]Potentials in V *versus* Fc/FcH^+; peak potential differences ΔE_p/mV in parentheses.

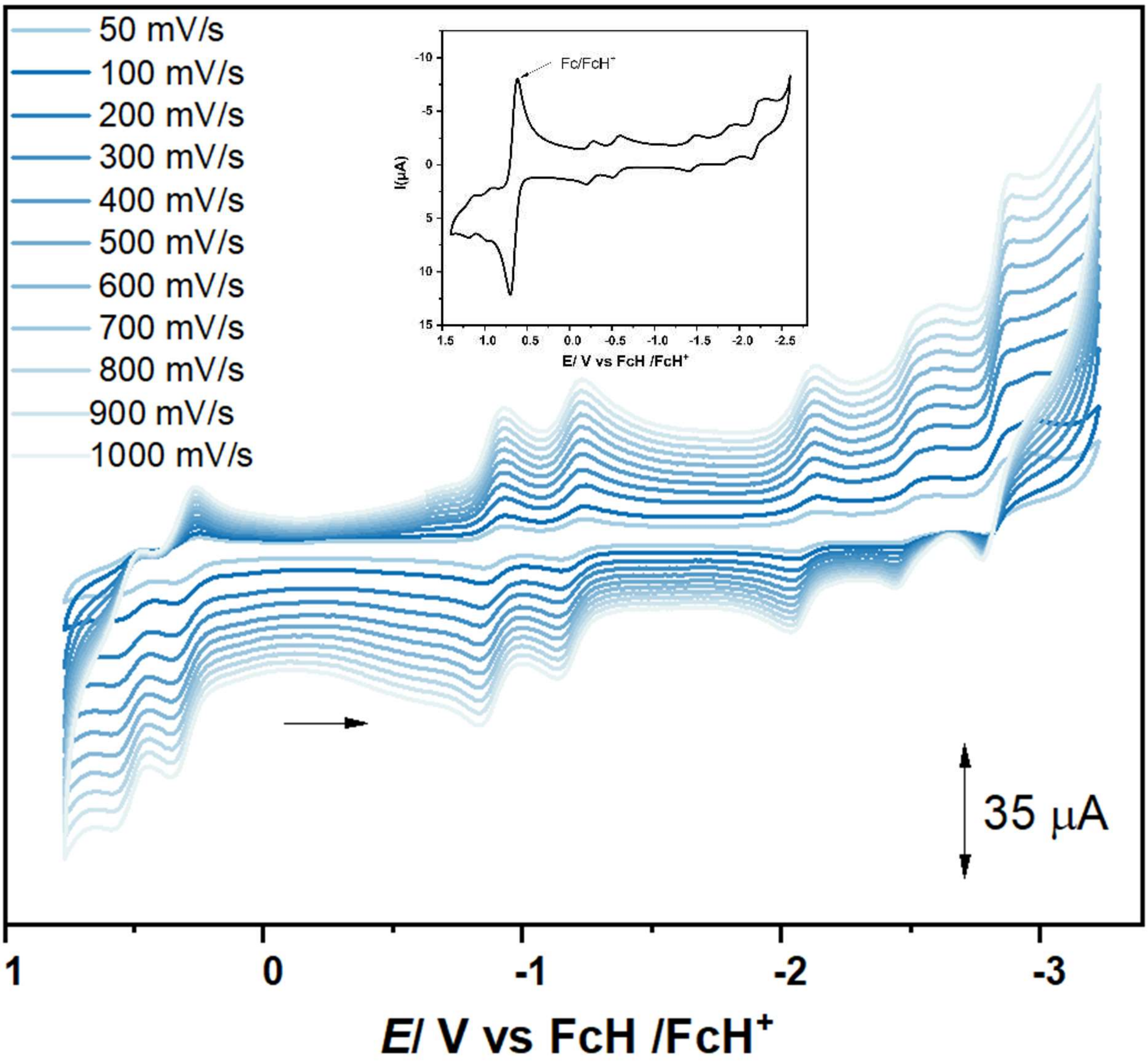

***Figure S13*.** Cyclic voltammograms of **1** in DMF containing 0.1M $^{n}Bu_4PF_6$ at different scan rates. (Conditions: GC working electrode, platinum wire as a counter electrode, and Fc/FcH^+ couple ($E_{1/2}$ = 0.63 v, inset, recorded at a scan rate of 100mV/s) as an internal reference.

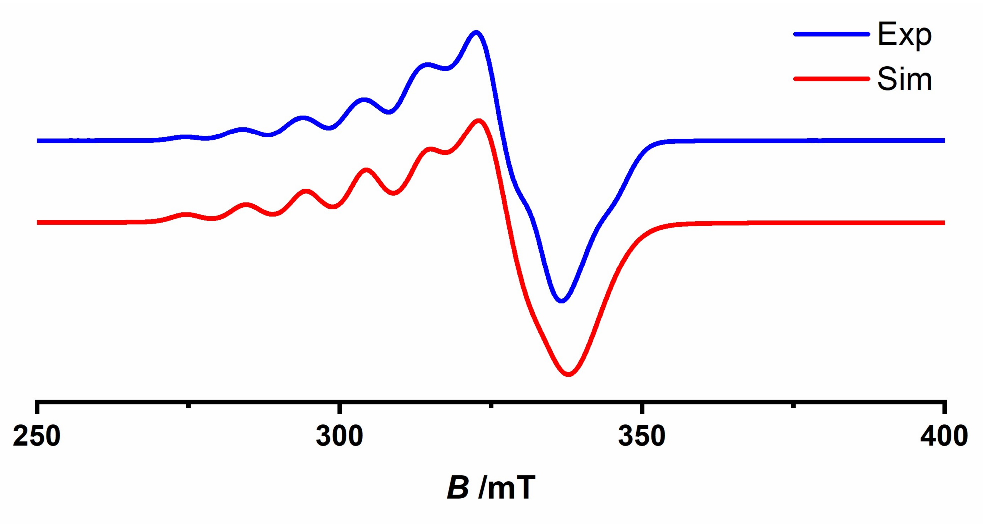

Figure S14. Experimental (blue) and simulated (red) X-band (9.47 GHz) EPR spectra of **1** measured at 98K.

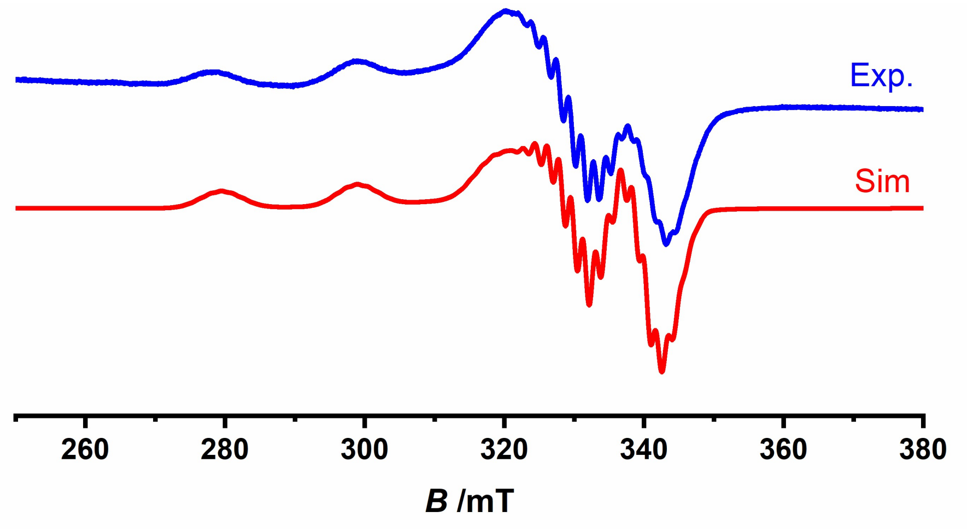

Figure S15. Experimental (blue) and simulated (red) X-band (9.47 GHz) EPR spectra of complex **2** measured at 98 K.

Table S2. EPR parameters obtained from the least-squares fitting of the simulated EPR spectra with experimental spectra.

Parameters	**1 (*S* = 1)**	**2(*S* = 1/2)**
g_x	2.0729	2.043
g_y	2.0098	2.045
g_z	2.2210	2.19
A_x (MHz)	20	64
A_y (MHz)	20	61
A_z (MHz)	308	596
lwpp (mT)[a]	0 0.7527	1.3 0.3
$H\text{Strain}_x$	270.8825	20
$H\text{Strain}_y$	314.3718	2
$H\text{Strain}_z$	153.1062	20
Temperature	98 K	98 K
State	Powder	Powder
[a]First and second values correspond to Gaussian and Lorentzian line widths, respectively.		

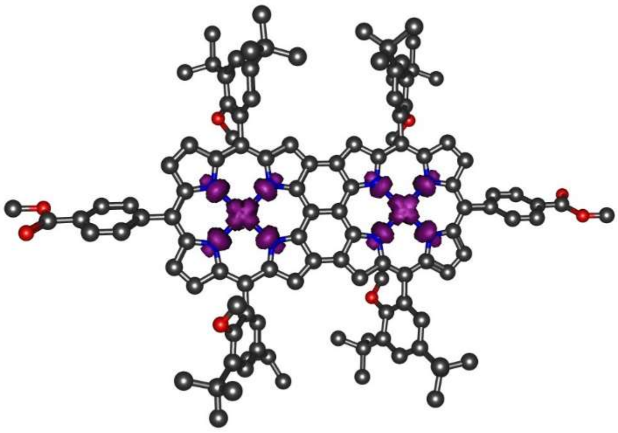

S = 1

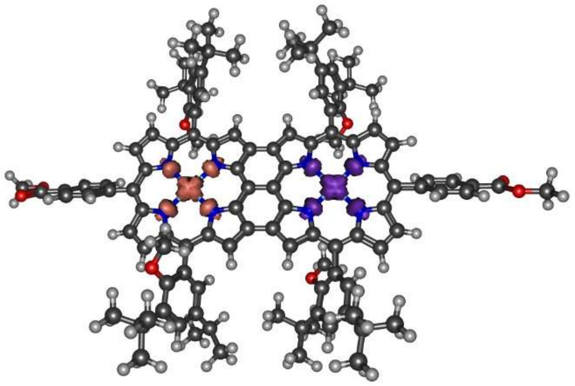

S =0

Figure S16. Spin density diagram of triplet and singlet state of complex **1**.

Complex	Final Gibbs Free energy		$\Delta E_{S=1-S=0}$
**1**	S = 1	S = 0	-0.0111 Hartree<br>-2436.16 cm^{-1}
	-8760.6330	-8760.6219	
Method: PBE0/def2-TZVP/SVP			

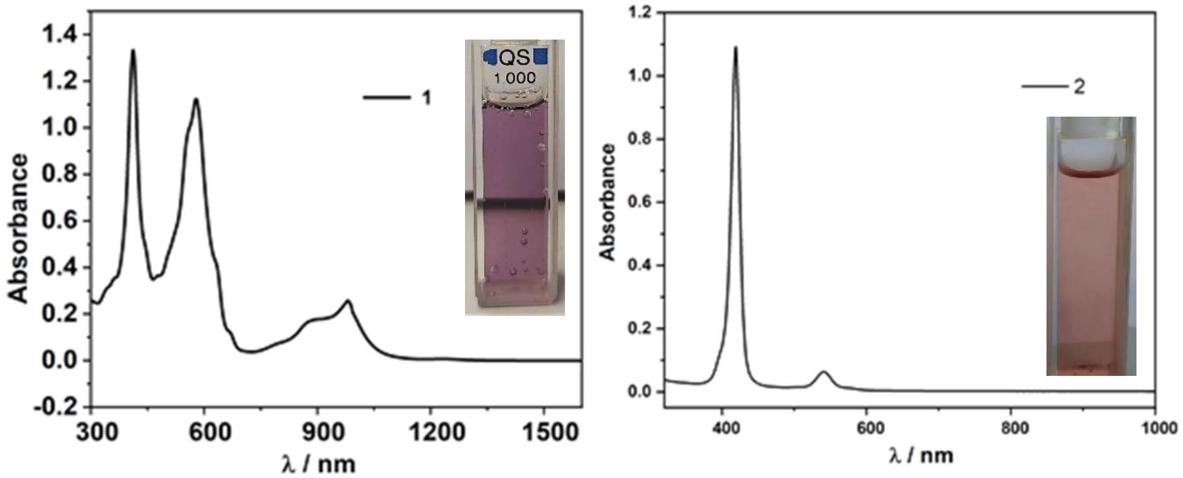

Figure S17. UV-Vis-NIR absorption spectrum of **1** and **2** (inset represents colour of the respective solution).

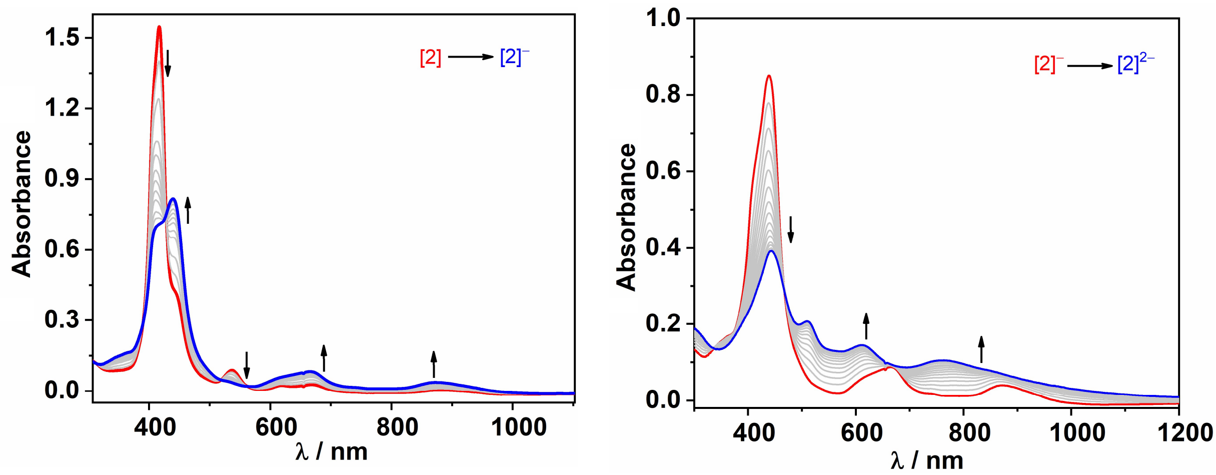

Figure S18. UV-Vis-NIR spectroelectrochemical responses of $\mathbf{2}^n$ in DMF/0.1 M ^{n}Bu$_4$PF$_6$ solution.

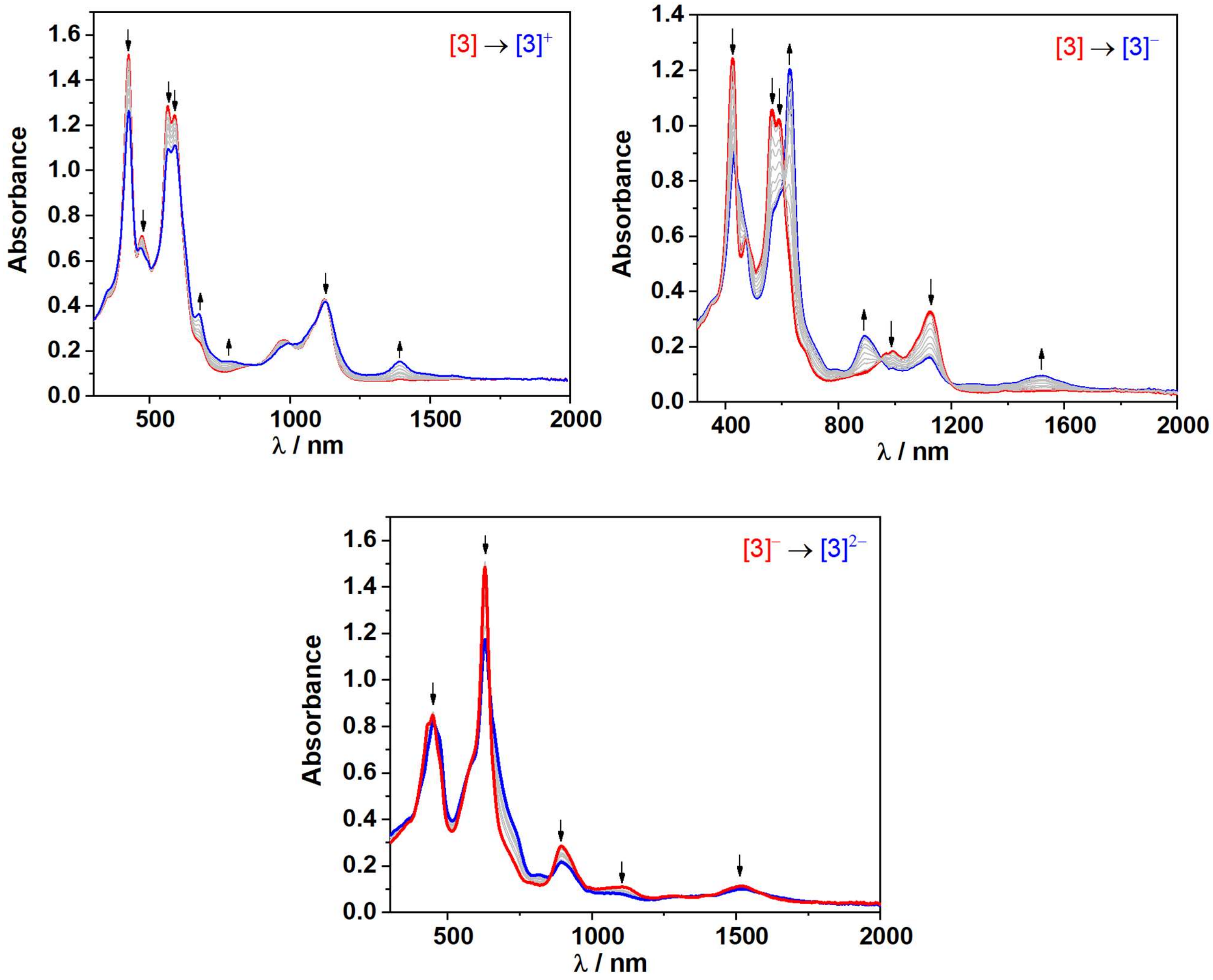

Figure S19. UV-Vis-NIR spectroelectrochemical responses of $\mathbf{3}^n$ in DMF/0.1 M ^{n}Bu$_4$PF$_6$ solution.

Table S3. Changes in the UV-Vis-NIR absorption bands on Spectroelectrochemical reduction of **1**, **2,** and **3**.

Complex	λ_{max} (in nm)
**1**	414, 576, 887, 986
$\mathbf{1}^{+}$	412, 588, 686, 728, 1029, 1313, 1569
$\mathbf{1}^{-}$	415, 611, 865, 1293
$\mathbf{1}^{2-}$	415, 611, 865, 1293, 1600
**2**	417, 536
$\mathbf{2}^{-}$	418, 666, 877
$\mathbf{2}^{2-}$	442, 511, 612, 764
**3**	425, 566, 591, 970, 995, 1125
$\mathbf{3}^{+}$	673, 779(sh), 1392
$\mathbf{3}^{-}$	629, 891, 1516
$\mathbf{3}^{2-}$	474, 727, 1521

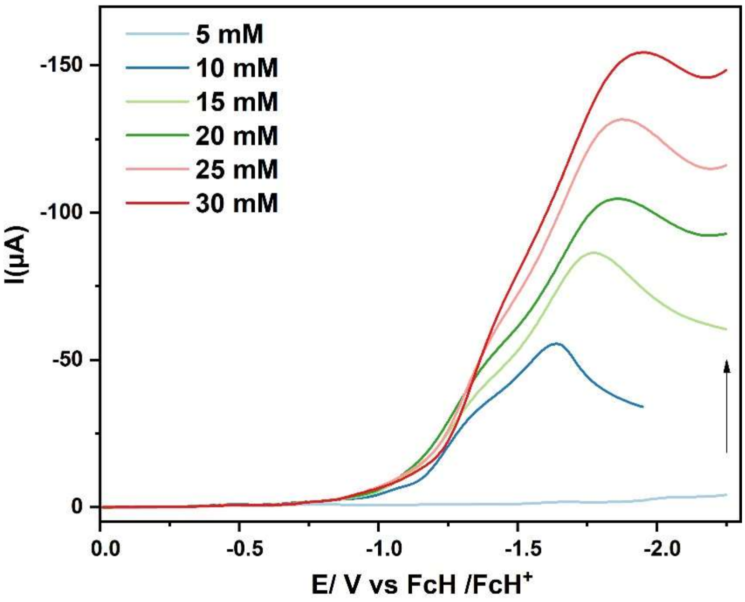

Figure S20. Linear sweep voltammograms of complex **1** with increasing concentration of TFA in DMF with 0.1 M $^{n}Bu_4PF_6$ at 100 mV/s (Conditions: GC working electrode, platinum wire as a counter electrode, and Fc/FcH$^+$ couple ($E_{1/2}$ = 0.35 V) as an internal reference.

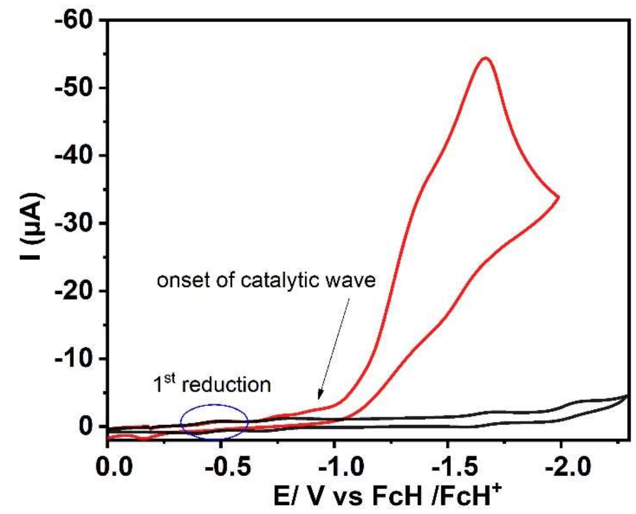

Figure S21. Cyclic voltammograms of the catalyst **1** before (black) and after (red) the addition of 5 mM of TFA. The marked area (in blue) indicate appearance of irreversible catalytic wave appears near to the $\mathbf{1}^{2-}/\mathbf{1}^{-}$ (second reduction) redox process without affecting the reversibility of the first reduction process. (Conditions: GC working electrode, platinum wire as a counter electrode, and Fc/FcH^{+} couple ($E_{1/2}$ = 0.35 V) as an internal reference.

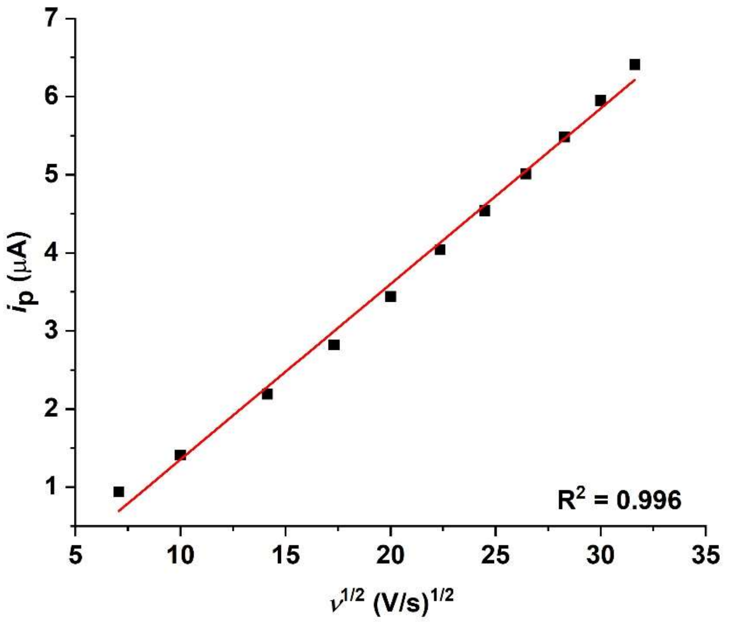

Figure S22. Plot of peak current (i_p) versus square root of the scan rate of the first reduction wave of complex **1** in the absence of acid. Linear dependence of peak current on the scan rate indicate diffusion controlled electrochemical process. Voltammograms were recorded with 0.01 mM solution of **1** with 0.1 M ^{n}Bu$_4$PF$_6$ at a scan rates of 50 – 1000 mV/s (Conditions: GC working electrode, platinum wire as a counter electrode, and Fc/FcH$^+$ couple ($E_{1/2}$ = 0.41 V) as an internal reference).

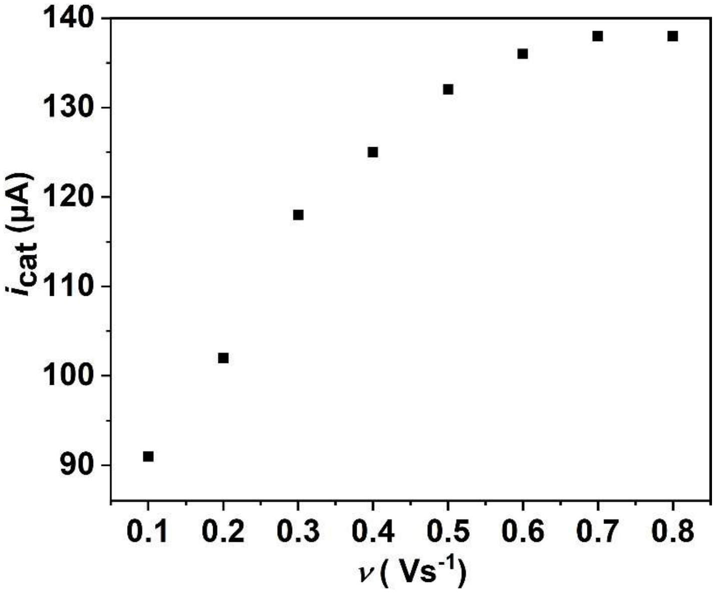

Figure S23**.** Plot of i_{cat} versus scan rate of complex **1**. i_{cat} increases linearly with scan rate until it reaches a limiting value of 500 mV/s, beyond which it becomes independent of scan rate. Voltammograms were recorded with 0.01 mM solution of **1** with 0.1 M $^{n}Bu_4PF_6$ in the presence of 15 mM TFA (Conditions: GC working electrode, platinum wire as a counter electrode, and Fc/FcH$^+$ couple as an internal reference).

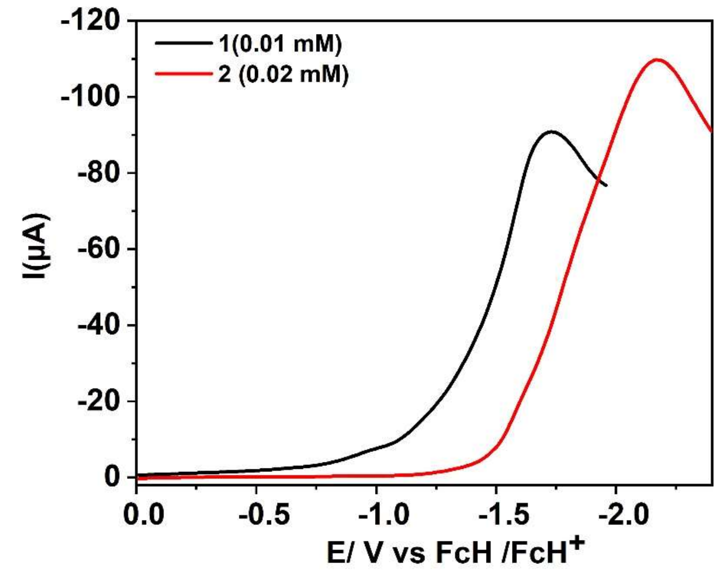

***Figure S24*.** Linear sweep voltammograms 0.01 mM and 0.02 mM DMF solutions of **1** (black) and **2** (red) with 0.1 M $^{n}Bu_4PF_6$ in the presence of 15 mM TFA at 100 mV/s (Conditions: GC working electrode, platinum wire as a counter electrode, and Fc/FcH^{+} couple ($E_{1/2}$ = 0.34 V) as an internal reference).

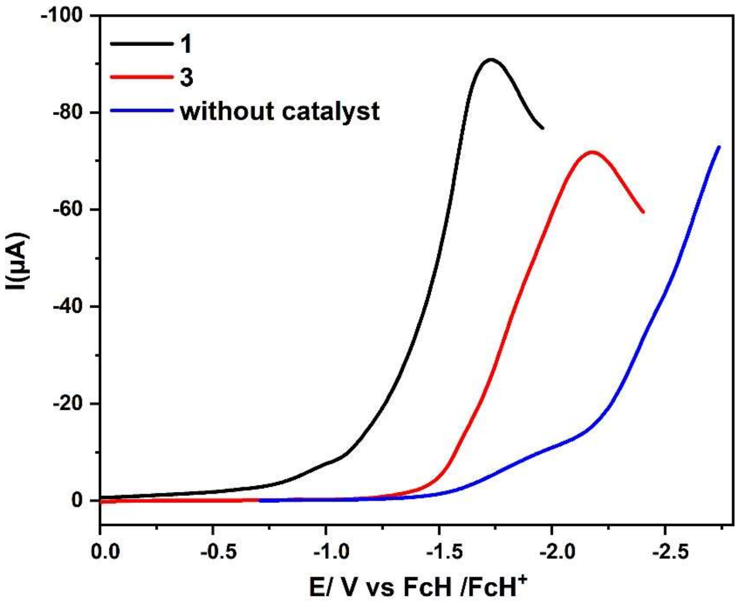

Figure S25. Linear sweep voltammograms of 0.01 mM DMF solutions of **1** (black) and **3** (red) with 0.1 M $^{n}Bu_4PF_6$ in the presence of 15 mM TFA at 100 mV/s. Blue line represents voltammograms of a solution containing 0.1 M $^{n}Bu_4PF_6$ in the presence of 15 mM TFA recorded at 100 mV/s in absence of a catalyst (Conditions: GC working electrode, platinum wire as a counter electrode, and Fc/FcH$^+$ couple ($E_{1/2}$ = 0.35 V) as an internal reference).

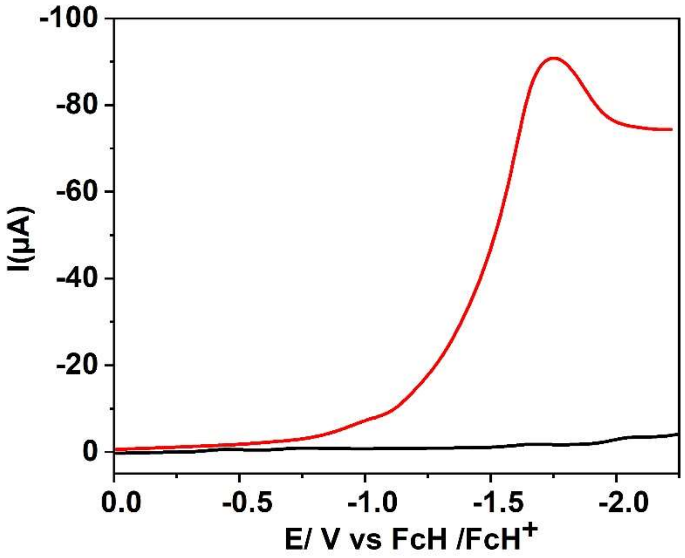

Figure S26. Linear sweep voltammogram of 0.01 mM DMF solutions of **1** (red) with 0.1 M ^{n}Bu$_4$PF$_6$ in the presence of 15 mM TFA at 100 mV/s. Glassy carbon electrode is then taken out from the solution and washed thoroughly with acetone before reusing it for recording liner sweep voltammogram (black line) with a fresh DMF solution containing 0.1 M ^{n}Bu$_4$PF$_6$ and 15 mM of TFA at 100 mV/s. (Conditions: GC working electrode, platinum wire as a counter electrode, and Fc/FcH$^+$ couple ($E_{1/2}$ = 0.35 V) as an internal reference).

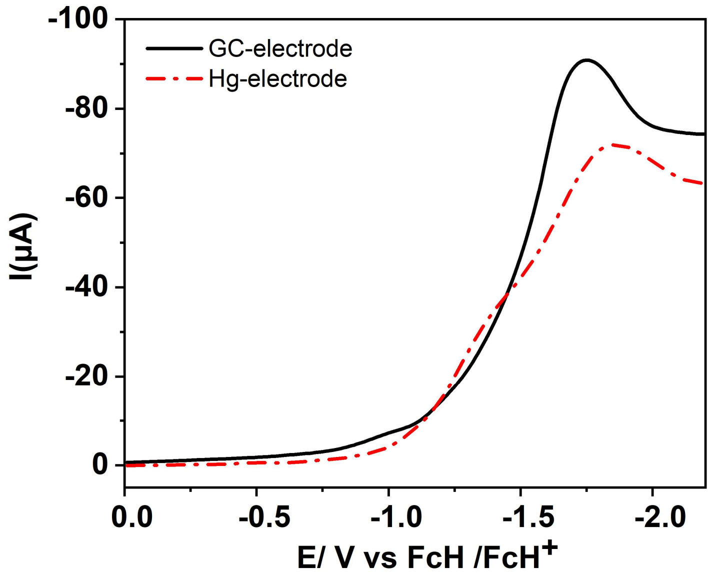

Figure S27. Linear sweep voltammogram of 0.01 mM DMF solutions of **1** containing 0.1 M $^{n}Bu_4PF_6$ in the presence of 15 mM TFA at 100 mV/s with GC electrode (black solid line) and Hg-electrode (red dot).

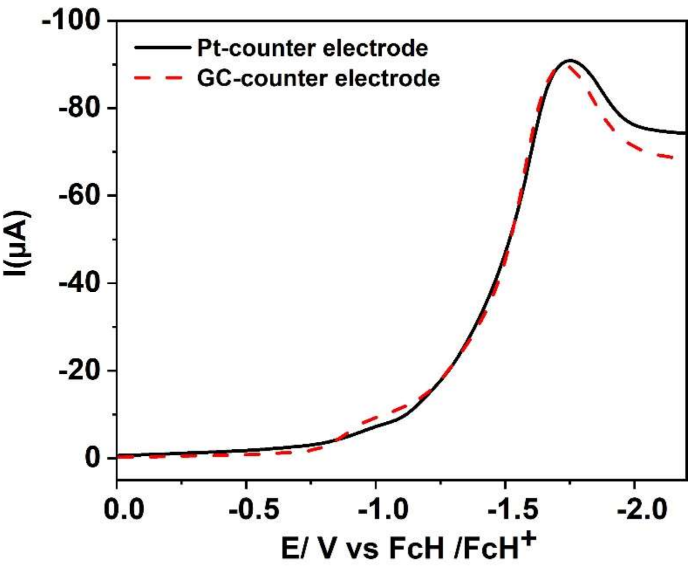

Figure S28. Linear sweep voltammogram of 0.01 mM DMF solutions of **1** containing 0.1 M $^{n}Bu_4PF_6$ in the presence of 15 mM TFA at 100 mV/s with glassy carbon disk (red dot) or platinum wire (black solid line) as a counter electrode. (Conditions: GC working electrode, Ag-wire as a pseudoreference and Fc/FcH$^+$ couple ($E_{1/2}$ = 0.38 V) as an internal reference).

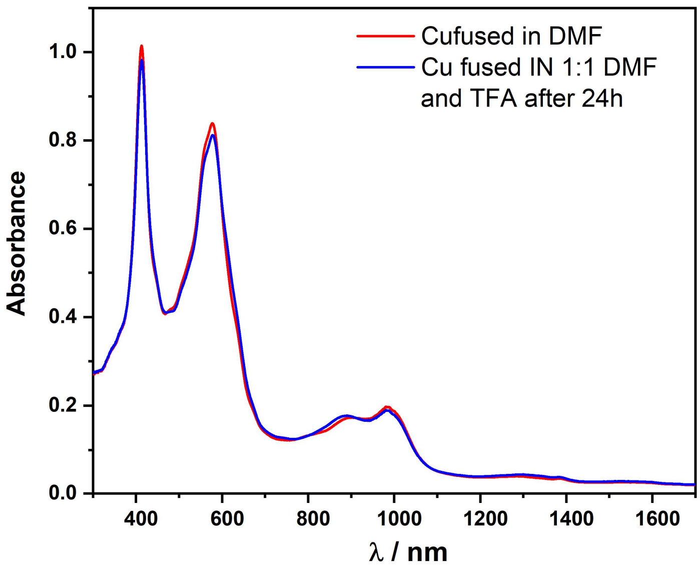

Figure S29. UV-Vis-NIR absorption spectrum of **1** in DMF and 1:1 mixture of DMF/TFA solutions in the beginning and after 24 hours.

Calculation of observed rate–constant (*k*obs).

The kinetic observed rate constant k_{obs} (also referred to as TOF) was calculated using the following relation

$$i_{cat} = n_c F A C_{cat}^0 \sqrt{Dk(\mathrm{H}^+)^x} \qquad \text{(S1)}$$

$$i_p = 0.4463 n_p F A C_{cat}^0 \sqrt{\frac{n_p F \vartheta D}{RT}} \qquad \text{(S2)}$$

where i_{cat} and i_p represents catalytic current and peak currents for non-catalytic reversible processes respectively, $n_c = 2$ is the number of electrons involved in the catalytic process, $n_p = 1$ denotes number of electrons involved in the reversible redox process of the catalyst, ν is the scan rate (V/s), D is the diffusion coefficients of the catalyst, F is the Faraday's constant, C_{cat}^0 is the active catalysts concentration in the bulk solution and A is the electrochemically active area; x is the order of the reaction with respect to acid concentration. Dividing equation 1 by equation 2 gives;

$$\frac{i_{cat}}{i_p} = \frac{n_c}{0.4463} \sqrt{\left(\frac{RT}{F\vartheta}\right) k(\mathrm{H}^+)^x} \qquad \text{(S3)}$$

$$k_{obs} = k(\mathrm{H}^+)^x = 1.94\, V^{-1}.\vartheta \left(\frac{i_{cat}}{i_p}\right)^2 \qquad \text{(S4)}$$

Table S4. Calculation of rate constant from the data obtained from the acid titration of 0.01 mM solution of **1** at 300 mV/s.

**Conc. of TFA (M)**	i_p **(μA)**	i_{cat}**(μA)**	i_{cat}/i_p	k_{obs}**(s^{-1})**
5 x 10^{-3}	4 x 10^{-2}	0.52 x 10^{2}	1.3 x 10^{3}	0.9 x 10^{6}
10 x 10^{-3}	4 x 10^{-2}	1.0765 x 10^{2}	2.69 x 10^{3}	0.41 x 10^{7}
15 x 10^{-3}	4 x 10^{-2}	1.18 x 10^{2}	2.95 x 10^{3}	0.5 x 10^{7}
20 x 10^{-3}	4 x 10^{-2}	1.47 x 10^{2}	3.67 x 10^{3}	0.7 x 10^{7}
25 x 10^{-3}	4 x 10^{-2}	1.64 x 10^{2}	4.1 x 10^{3}	0.9 x 10^{7}
30 x 10^{-3}	4 x 10^{-2}	1.79 x 10^{2}	4.4 x 10^{3}	1.1 x 10^{7}
40 x 10^{-3}	4 x 10^{-2}	2.33 x 10^{2}	5.8 x 10^{3}	1.9 x 10^{7}
50 x 10^{-3}	4 x 10^{-2}	2.46 x 10^{2}	6.1 x 10^{3}	2.2 x 10^{7}

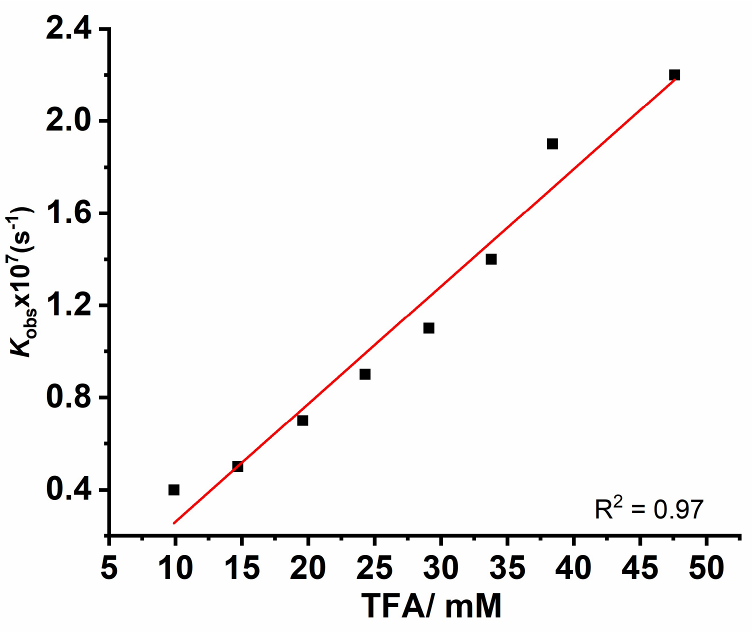

Figure S30. Plot of k_{obs} versus concentration of acid.

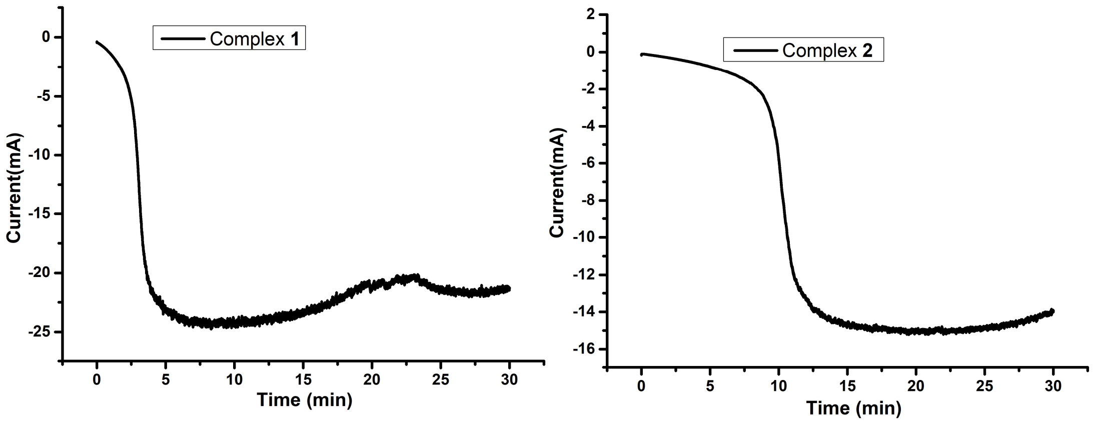

(a) **(b)**

Figure S31. Current versus time curve during electrolysis of (a) **1** and (b) **2** at -1.05 V. Conditions: Electrolysis was carried out with 0.06 mM and 0.12 mM DMF solution (30 mL) of **1** and **2** respectively with 0.1 M $^{n}Bu_4PF_6$ in the presence of 0.1 M of TFA.

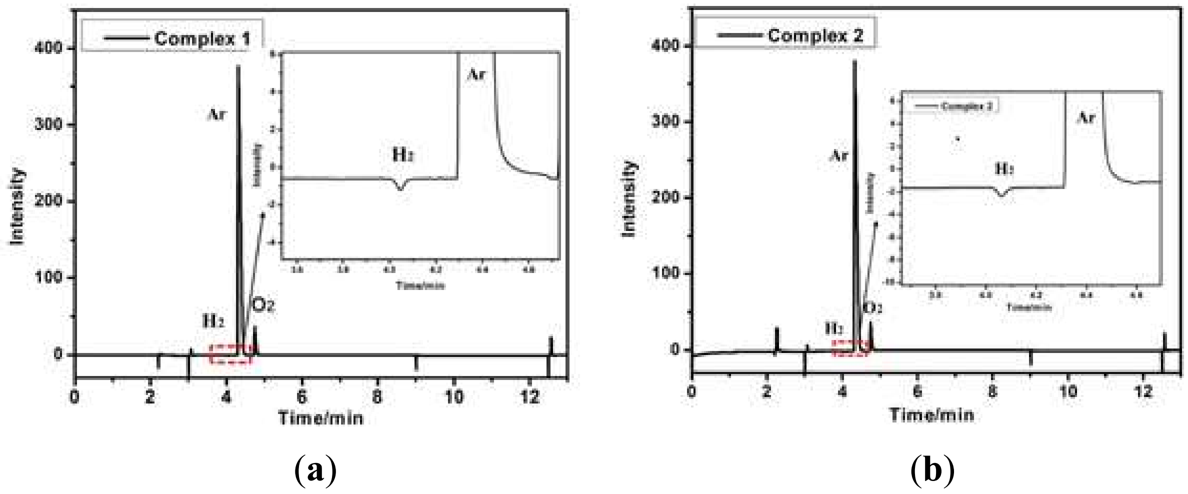

Figure S32. Products detection. GC plots of the gas products over Complex **1**(a) and Complex **2** (b). The tiny amount of O_2 may come from the syringe during the injection to GC.

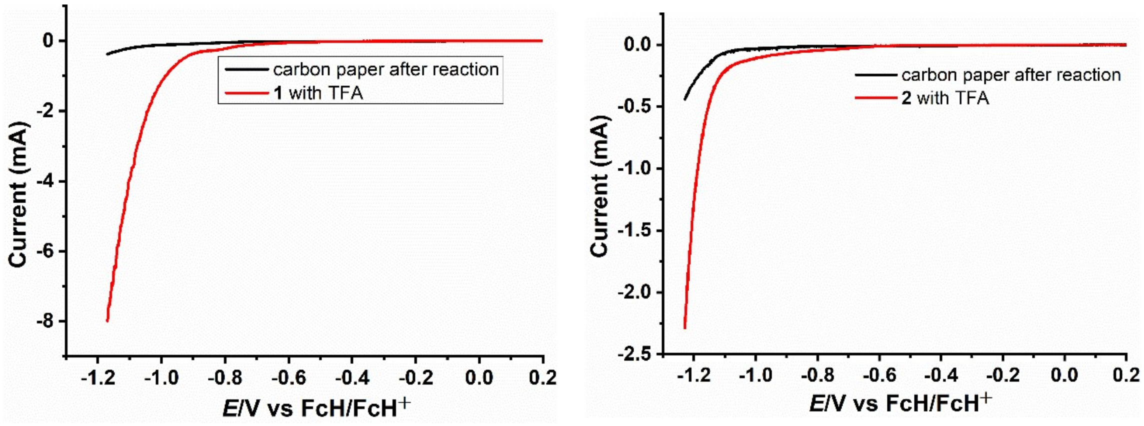

(a) (b)

Figure S33**.** Linear sweep voltammograms of 0.06 and 0.12 mM DMF solutions of (a) **1** and (b) **2** with 0.1 M $^{n}Bu_4PF_6$ in the presence of 0.1 M TFA during the controlled potential electrolysis process. Carbon paper is then taken out from the solution and washed thoroughly with DMF three times before reusing it for recording liner sweep voltammogram (black line) with a fresh DMF solution containing 0.1 M $^{n}Bu_4PF_6$ and 0.1 M of TFA solution.

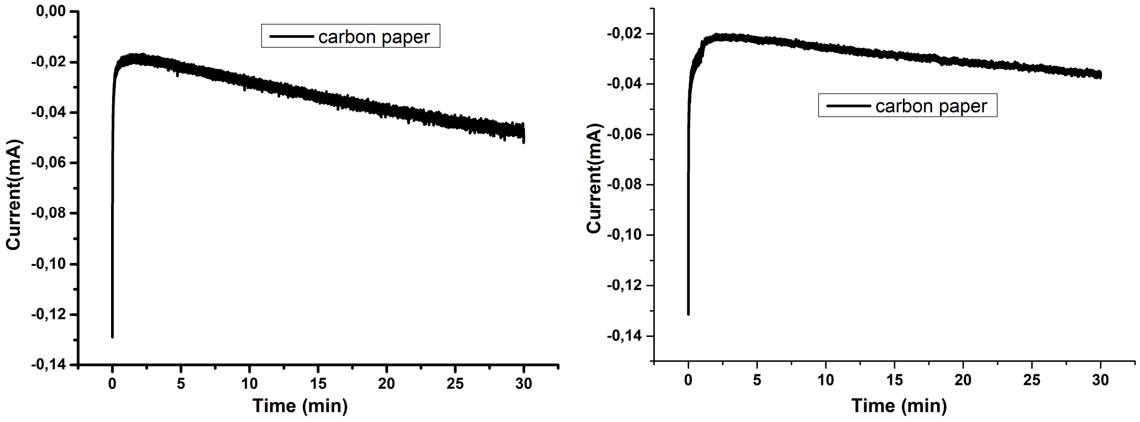

(a) **(b)**

Figure S34. Current *versus* time plot with the rinsed carbon paper working electrode at a constant potential of -1.05 V. Plot (a) and (b) indicate electrolysis process carried out with the electrode taken out of the solution of complexes **1** and **2** respectively.

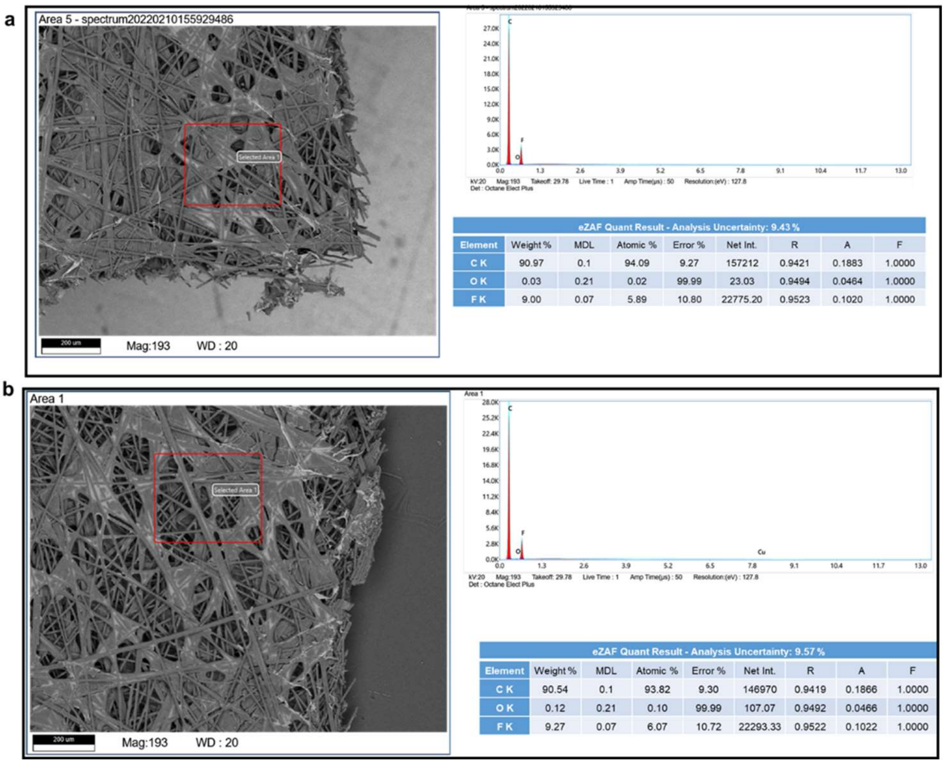

Figure S35. (a) and (b) indicate SEM/EDX results carried out with the electrodes taken out of the solution of complexes **1** and **2** after bulk electrolysis. Reaction condition: constant potential of -1.05 V *vs.* Fc/FcH$^+$ for 30min.

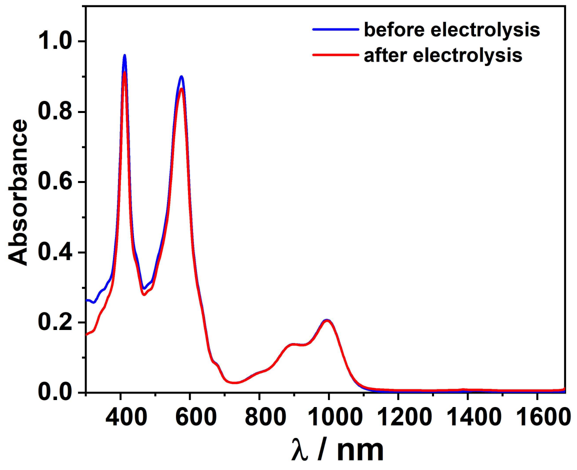

Figure S36. UV-Vis-NIR absorption spectrum of **1** in DMF before (blue) and after (red) bulk-electrolysis.

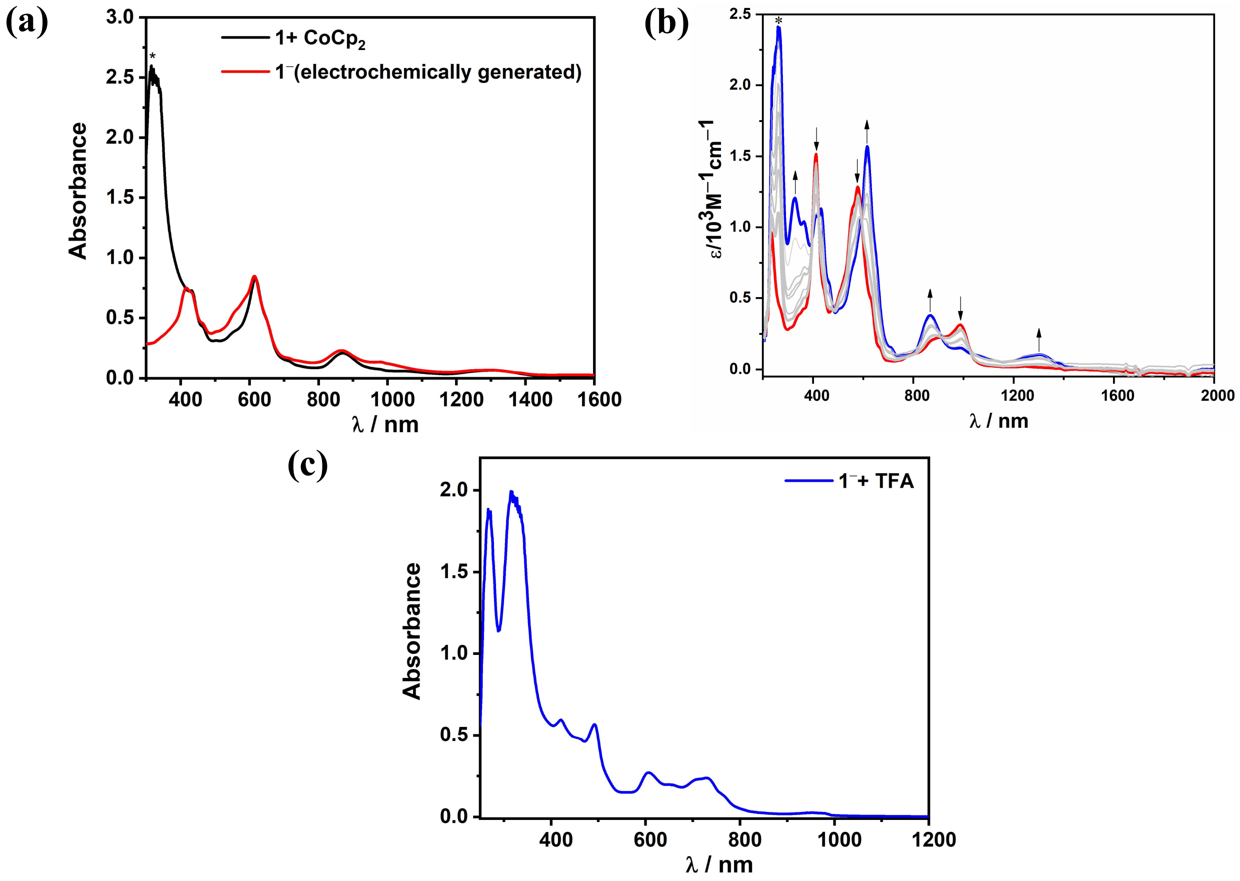

Figure S37. (a) UV-Vis-NIR absorption spectra of the electrochemically (red) and chemically (black) generated one-electron reduced species of complex **1**. *indicates absorption peaks corresponding to the cobaltocene. (b) Chemical reduction of **1** with increasing concentration of Cobaltocene (* indicates absorption peaks corresponding to the cobaltocene.), (c) Absorption spectra of the reaction of one electron reduced species (**1**⁻) with TFA.

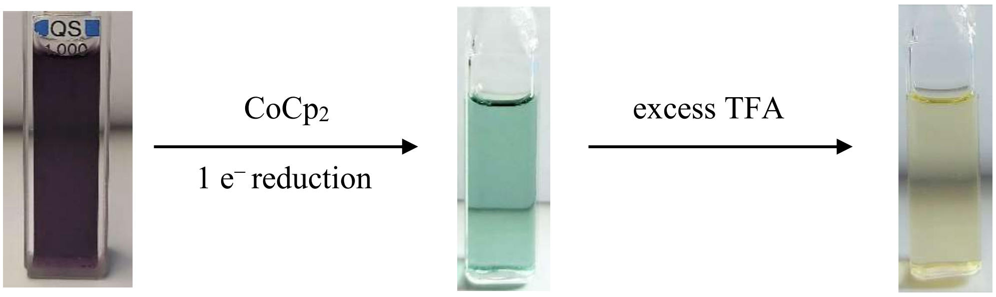

Figure S38. Change in colour of the DMF solution of **1** upon one electron reduction (light green) and reaction of the reduced species with TFA (yellow).

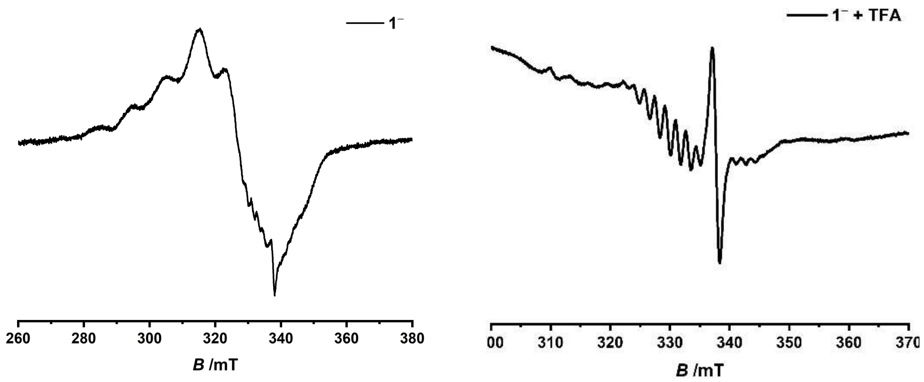

Figure S39. EPR spectra of the one electron reduced species (**1**⁻) of the catalyst **1** (left) and solution of one electron reduced species with TFA (**1**⁻ + TFA) measured at -175°C.

[1] I. S. Weitz, M. Rabinovitz, *J. Chem. Soc., Perkin Trans. 1* **1993**, 117.
[2] G. R. Fulmer, A. J. M. Miller, N. H. Sherden, H. E. Gottlieb, A. Nudelman, B. M. Stoltz, J. E. Bercaw, K. I. Goldberg, *Organometallics* **2010**, *29*, 2176.
[3] M. Krejčik, M. Daněk, F. Hartl, *J. Electroanal. Chem. Interf. Electrochem.* **1991**, *317*, 179.
[4] S. Stoll, A. Schweiger, *J. Magn. Reson.* **2006**, *178*, 42.
[5] F. Neese, *WIREs Comput Mol Sci.* **2018**, *8*.
[6] C. Adamo, V. Barone, *J. Chem. Phys.* **1999**, *110*, 6158.
[7] F. Weigend, R. Ahlrichs, *Phys. Chem. Chem. Phys.* **2005**, *7*, 3297.
[8] V. Barone, M. Cossi, *J. Phys. Chem. A* **1998**, *102*, 1995.
[9] a) S. Grimme, *J. Comput. Chem.* **2006**, *27*, 1787; b) S. Grimme, *J. Comput. Chem.* **2004**, *25*, 1463; c) S. Grimme, J. Antony, S. Ehrlich, H. Krieg, *J. Chem. Phys.* **2010**, *132*, 154104; d) S. Grimme, S. Ehrlich, L. Goerigk, *J. Comput. Chem.* **2011**, *32*, 1456.
[10] a) P. Seth, P. L. Ríos, R. J. Needs, *J. Chem. Phys.* **2011**, *134*, 84105; b) F. Neese, G. Olbrich, *Chem. Phys. Lett.* **2002**, *362*, 170; c) R. Izsák, F. Neese, *J. Chem. Phys.* **2011**, *135*, 144105; d) J. L. Whitten, *J. Chem. Phys.* **1973**, *58*, 4496; e) O. Vahtras, J. Almlöf, M. W. Feyereisen, *Chem. Phys. Lett.* **1993**, *213*, 514; f) F. Neese, F. Wennmohs, A. Hansen, *J. Chem. Phys.* **2009**, *130*, 114108; g) F. Neese, *J. Comput. Chem.* **2003**, *24*, 1740.
[11] a) K. Eichkorn, O. Treutler, H. Öhm, M. Häser, R. Ahlrichs, *Chem. Phys. Lett.* **1995**, *240*, 283; b) K. Eichkorn, F. Weigend, O. Treutler, R. Ahlrichs, *Theor. Chem. Acc.* **1997**, *97*, 119; c) F. Weigend, *Phys. Chem. Chem. Phys.* **2006**, *8*, 1057.
[12] P.-O. Löwdin, *J. Chem. Phys.* **1950**, *18*, 365.
[13] a) D. Doehnert, J. Koutecky, *J. Am. Chem. Soc.* **1980**, *102*, 1789; b) J. Gräfenstein, E. Kraka, M. Filatov, D. Cremer, *Int. J. Mol. Sci.* **2002**, *3*, 360; c) F. Neese, *J. Phys. Chem. Solids* **2004**, *65*, 781; d) K. Yamaguchi, *Chem. Phys. Lett.* **1975**, *33*, 330.
[14] *Chemcraft - graphical software for visualization of quantum chemistry computations*, Chemcraft.
[15] F. C. Gaenzler, C. Guo, Y.-W. Zhang, M. E. Azab, M. A.I. Salem, D. P. Fan, M. B. Smith, *Tetrahedron* **2009**, *65*, 8781.
[16] S. M. Elbert, P. Wagner, T. Kanagasundaram, F. Rominger, M. Mastalerz, *Chem. Eur.J* **2017**, *23*, 935.
[17] B. J. Littler, M. A. Miller, C.-H. Hung, R. W. Wagner, D. F. O'Shea, P. D. Boyle, J. S. Lindsey, *J. Org. Chem.* **1999**, *64*, 1391.
[18] T. Rohand, E. Dolusic, T. H. Ngo, W. Maes, W. Dehaen, *Arkivoc* **2007**, *2007*, 307.

Cartesian coordinates of the computed structures:

1 (triplet)

N	14.122104000	10.366010000	5.473574000
N	14.250013000	12.330338000	3.402117000
N	15.001261000	4.598624000	7.742567000
N	15.169869000	8.397907000	3.675505000
N	14.970496000	2.646901000	9.827460000
N	13.958396000	6.564497000	9.540452000
N	15.326339000	10.359007000	1.605237000
N	13.875828000	4.609911000	11.624187000
O	14.704685000	15.208960000	6.548824000
C	15.011648000	7.601378000	4.781668000
C	13.763817000	9.711635000	7.675692000
C	14.187900000	9.310800000	6.347980000
C	14.099576000	7.361083000	8.432206000
C	14.578502000	7.984867000	6.068343000
C	14.346308000	13.125517000	2.274814000
C	14.528100000	6.977066000	7.144403000
C	13.728891000	8.730783000	8.729090000
C	14.918521000	5.651395000	6.866039000
C	15.369720000	6.228634000	4.483855000
O	14.862131000	-1.533306000	17.549303000
C	15.630340000	7.560847000	2.667931000
O	16.029627000	17.820252000	-2.841232000
C	15.019660000	13.782772000	-0.028957000
O	14.488172000	7.800218000	14.335923000
C	13.433986000	11.053836000	7.587459000
H	13.076963000	11.714173000	8.384352000
C	12.766973000	13.732276000	6.620671000
C	15.332374000	5.249026000	5.536364000
C	15.404965000	11.453331000	0.762467000
C	16.518892000	6.941664000	0.421724000

C	15.982286000	7.965370000	1.366286000
C	13.644216000	11.440016000	6.211083000
C	13.433031000	14.934896000	6.981156000
C	14.922607000	12.749657000	1.042226000
C	15.483702000	3.527796000	7.002720000
C	13.369232000	8.753167000	10.066445000
H	13.043059000	9.612016000	10.661657000
O	14.636564000	7.182136000	-1.105005000
C	10.789335000	14.415245000	7.912225000
C	14.335426000	1.222896000	13.297685000
C	15.736059000	6.203084000	3.149170000
H	16.058512000	5.341921000	2.555361000
C	16.475099000	1.301565000	6.582077000
C	15.599295000	1.883678000	8.860045000
C	12.609421000	8.004077000	12.798680000
C	11.465997000	13.489969000	7.100615000
H	10.976564000	12.551791000	6.799085000
C	13.380340000	12.726599000	5.704038000
C	13.842742000	3.521888000	12.478364000
C	15.829049000	2.264403000	7.521236000
C	13.633244000	13.105272000	4.367914000
C	14.368100000	2.240240000	12.208056000
C	15.885092000	9.299824000	0.913438000
C	13.495007000	7.397089000	10.549689000
C	15.203061000	15.749729000	-2.057723000
C	16.350059000	9.741685000	-0.382016000
H	16.852532000	9.108441000	-1.120537000
C	14.934538000	1.868026000	10.969297000
C	13.749181000	14.420623000	2.529237000
H	13.661348000	15.233623000	1.801186000
C	15.671653000	3.911042000	5.623918000
H	16.028083000	3.254082000	4.824488000

O	13.555995000	-2.803166000	16.198829000
O	18.440819000	2.717088000	6.298093000
C	11.460118000	15.615907000	8.211579000
H	10.946700000	16.358483000	8.831484000
C	16.059413000	11.079776000	-0.474685000
H	16.281653000	11.757112000	-1.305641000
C	12.767169000	15.912740000	7.772020000
C	13.306569000	14.405925000	3.828432000
H	12.790935000	15.203024000	4.373949000
C	17.710720000	1.611766000	5.948845000
C	13.161551000	6.988214000	11.855627000
C	13.293543000	5.658420000	12.313422000
C	13.271455000	8.338874000	14.007616000
C	16.000466000	0.609643000	9.414586000
H	16.540877000	-0.173069000	8.871686000
C	14.274652000	-0.712413000	15.364221000
C	11.369804000	8.607334000	12.491949000
H	10.886370000	8.321908000	11.548327000
C	13.188780000	3.886100000	13.718627000
H	12.996001000	3.209726000	14.557698000
C	15.262818000	16.754461000	-3.162014000
C	9.375777000	14.091216000	8.427705000
C	15.584259000	0.597252000	10.723448000
H	15.724319000	-0.198443000	11.462413000
C	12.852816000	5.212738000	13.616144000
H	12.336374000	5.835835000	14.353609000
C	15.789820000	0.126302000	6.222630000
H	14.835480000	-0.085633000	6.727903000
O	14.688301000	16.637096000	-4.235225000
C	15.845815000	6.619669000	-0.787380000
C	10.756597000	9.514296000	13.365588000
C	11.415146000	9.778568000	14.585998000

H	10.937508000	10.472725000	15.289076000
C	12.656193000	9.222503000	14.943327000
C	14.277466000	-1.697381000	16.487762000
C	18.189979000	0.807310000	4.878823000
C	14.346596000	13.611915000	-1.260658000
H	13.735530000	12.712080000	-1.424606000
C	17.750561000	6.319110000	0.713877000
H	18.263660000	6.578052000	1.653604000
C	9.426088000	10.216508000	13.042145000
C	18.343223000	5.411827000	-0.174911000
C	14.435263000	14.584968000	-2.260424000
H	13.905268000	14.458456000	-3.215715000
C	14.990325000	0.490266000	15.533714000
H	15.528265000	0.659067000	16.477961000
C	15.880515000	15.925686000	-0.833257000
H	16.486537000	16.827337000	-0.669567000
C	15.018428000	1.447447000	14.515387000
H	15.589175000	2.377579000	14.653448000
C	13.446688000	17.246084000	8.152573000
C	17.677069000	5.154581000	-1.389331000
H	18.145104000	4.459028000	-2.097519000
C	15.785858000	14.954026000	0.169099000
H	16.325016000	15.094619000	1.117589000
C	16.437481000	5.728902000	-1.730532000
C	15.731475000	14.232261000	6.730803000
H	15.812663000	13.539343000	5.867424000
H	15.572397000	13.641524000	7.657008000
H	16.686537000	14.787015000	6.820576000
C	17.448478000	-0.347062000	4.552624000
H	17.819564000	-0.981628000	3.740701000
C	13.622493000	0.013407000	13.132681000
H	13.076303000	-0.169949000	12.195599000

C	16.268163000	-0.731568000	5.217482000
C	13.588450000	-0.942726000	14.153716000
H	13.022878000	-1.874471000	14.015886000
C	13.325362000	9.569386000	16.290717000
C	13.816933000	18.028260000	6.870894000
H	14.511220000	17.454034000	6.230258000
H	14.302721000	18.990678000	7.136411000
H	12.909280000	18.262563000	6.276493000
C	13.570627000	7.133389000	-0.154669000
H	12.626211000	7.134718000	-0.734554000
H	13.564515000	8.015779000	0.518889000
H	13.613293000	6.208928000	0.458298000
C	16.140767000	18.836459000	-3.848270000
H	16.587626000	18.427552000	-4.776901000
H	16.796687000	19.618198000	-3.425456000
H	15.147330000	19.265217000	-4.090476000
C	19.694847000	4.786556000	0.146452000
H	19.829716000	4.872443000	1.246903000
C	19.461423000	1.188875000	4.090730000
C	13.505886000	-3.803415000	17.226549000
H	12.882873000	-4.623593000	16.827518000
H	13.051399000	-3.397817000	18.152939000
H	14.521493000	-4.179627000	17.463945000
C	15.557065000	7.856621000	13.389294000
H	15.577169000	6.970732000	12.720692000
H	16.498647000	7.871492000	13.973413000
H	15.504527000	8.777132000	12.771123000
C	14.713154000	16.972178000	8.996984000
H	14.459013000	16.412949000	9.921129000
H	15.182129000	17.931033000	9.302362000
H	15.467589000	16.389143000	8.438128000
C	12.518478000	18.143985000	8.992078000

H	11.587149000	18.410137000	8.451930000
H	13.043303000	19.091784000	9.228458000
H	12.236626000	17.676073000	9.957324000
C	15.747875000	5.393532000	-3.070616000
C	20.693159000	1.161709000	5.024910000
H	20.593802000	1.879845000	5.859477000
H	21.609937000	1.423298000	4.455902000
H	20.841053000	0.149547000	5.455035000
C	18.693274000	2.986074000	7.677793000
H	19.660675000	3.524671000	7.724613000
H	18.776105000	2.048996000	8.266948000
H	17.913194000	3.633257000	8.130571000
C	12.453886000	10.517820000	17.135204000
H	12.284797000	11.493856000	16.636401000
H	12.965891000	10.725661000	18.096590000
H	11.465121000	10.077884000	17.377635000
C	19.288575000	2.598629000	3.475125000
H	18.415787000	2.626206000	2.790412000
H	20.189321000	2.867529000	2.885687000
H	19.139713000	3.370063000	4.252412000
C	20.830872000	5.582045000	-0.521113000
H	20.741378000	5.548134000	-1.627800000
H	21.822985000	5.165032000	-0.248752000
H	20.813341000	6.647975000	-0.215076000
C	8.370716000	9.831467000	14.102286000
H	8.184969000	8.737565000	14.103224000
H	7.406362000	10.340715000	13.894757000
H	8.687260000	10.121538000	15.124661000
C	8.813967000	15.209263000	9.321451000
H	8.718869000	16.171243000	8.777586000
H	9.443983000	15.381739000	10.217957000
H	7.801422000	14.931041000	9.678243000

C	9.648196000	11.745705000	13.071432000
H	9.962723000	12.098632000	14.074387000
H	8.712832000	12.281753000	12.807580000
H	10.431365000	12.050380000	12.347364000
C	19.732649000	0.211477000	2.931852000
H	19.918107000	-0.823700000	3.283966000
H	20.638975000	0.538655000	2.382856000
H	18.898615000	0.184373000	2.201108000
C	8.892783000	9.824065000	11.654208000
H	9.602381000	10.089978000	10.844604000
H	7.942093000	10.357271000	11.450912000
H	8.683375000	8.737368000	11.581896000
C	8.426074000	13.898240000	7.224642000
H	8.365138000	14.820824000	6.611164000
H	7.401160000	13.652440000	7.573290000
H	8.759937000	13.073614000	6.562937000
C	19.775660000	3.298418000	-0.220472000
H	18.948372000	2.715202000	0.231959000
H	20.731436000	2.860989000	0.134164000
H	19.738383000	3.142295000	-1.319049000
C	9.429507000	12.786327000	9.254224000
H	9.791248000	11.928436000	8.651999000
H	8.421039000	12.524718000	9.636259000
H	10.105346000	12.895480000	10.126575000
C	13.541254000	8.278057000	17.113835000
H	12.572377000	7.780714000	17.327963000
H	14.017502000	8.519809000	18.087180000
H	14.188749000	7.558329000	16.580032000
C	14.387589000	4.705768000	-2.809604000
H	14.521570000	3.773031000	-2.223592000
H	13.908598000	4.431830000	-3.772973000
H	13.688889000	5.361258000	-2.258822000

C	16.594923000	4.431154000	-3.924288000
H	17.587495000	4.855516000	-4.178329000
H	16.068657000	4.230398000	-4.879483000
H	16.754720000	3.453239000	-3.426115000
C	15.544152000	6.688336000	-3.891413000
H	14.914378000	7.418465000	-3.350597000
H	15.053515000	6.454472000	-4.859544000
H	16.518599000	7.169860000	-4.116212000
C	14.677051000	10.278770000	16.043106000
H	15.390968000	9.634590000	15.498355000
H	15.142494000	10.559618000	17.011116000
H	14.533618000	11.209698000	15.456436000
C	15.490816000	-2.013945000	4.869902000
C	15.400946000	-2.903012000	6.130552000
H	16.410579000	-3.193120000	6.488096000
H	14.836076000	-3.833432000	5.912274000
H	14.883475000	-2.386236000	6.964146000
C	16.171992000	-2.819607000	3.751191000
H	16.237592000	-2.245894000	2.804199000
H	15.586790000	-3.737619000	3.539885000
H	17.196685000	-3.138299000	4.031773000
C	14.065903000	-1.637965000	4.405574000
H	13.505237000	-1.091052000	5.190324000
H	13.484718000	-2.550123000	4.155460000
H	14.096524000	-0.995159000	3.501631000
Cu	14.715443000	10.365067000	3.535930000
Cu	14.451096000	4.602979000	9.686120000

1 (singlet)

N	14.122517000	10.366011000	5.473491000
N	14.251452000	12.330507000	3.402100000
N	15.002439000	4.599161000	7.743112000
N	15.170748000	8.397801000	3.675722000

N	14.971270000	2.647390000	9.828080000
N	13.958395000	6.564897000	9.540493000
N	15.326478000	10.358592000	1.604958000
N	13.877259000	4.610797000	11.624895000
O	14.705103000	15.210517000	6.545676000
C	15.012151000	7.601371000	4.781809000
C	13.764621000	9.712019000	7.675822000
C	14.188519000	9.310991000	6.347989000
C	14.099828000	7.361395000	8.432318000
C	14.578973000	7.984966000	6.068467000
C	14.347232000	13.125394000	2.274542000
C	14.528572000	6.977334000	7.144574000
C	13.729334000	8.731275000	8.729157000
C	14.918904000	5.651582000	6.866348000
C	15.369659000	6.228367000	4.483970000
O	14.862837000	-1.531775000	17.550668000
C	15.630668000	7.560588000	2.668058000
O	16.031296000	17.819761000	-2.841457000
C	15.020577000	13.782407000	-0.029281000
O	14.485716000	7.798758000	14.336960000
C	13.435252000	11.054303000	7.587485000
H	13.078696000	11.714936000	8.384342000
C	12.768349000	13.732701000	6.620436000
C	15.332345000	5.248946000	5.536508000
C	15.405463000	11.452913000	0.762199000
C	16.517622000	6.941077000	0.421151000
C	15.982418000	7.964940000	1.366297000
C	13.644948000	11.440183000	6.210891000
C	13.434075000	14.935933000	6.979506000
C	14.923303000	12.749331000	1.041902000
C	15.484214000	3.528060000	7.003289000
C	13.368888000	8.753536000	10.066294000

H	13.042476000	9.612322000	10.661488000
O	14.636912000	7.187366000	-1.106641000
C	10.791543000	14.415461000	7.913341000
C	14.337023000	1.223918000	13.298606000
C	15.735699000	6.202681000	3.149226000
H	16.057641000	5.341413000	2.555281000
C	16.476194000	1.302056000	6.582908000
C	15.600427000	1.884259000	8.860859000
C	12.608252000	8.004076000	12.798502000
C	11.467873000	13.490101000	7.101585000
H	10.978741000	12.551295000	6.801512000
C	13.381310000	12.726808000	5.703814000
C	13.843838000	3.522717000	12.479007000
C	15.829834000	2.264778000	7.521928000
C	13.634245000	13.105361000	4.367647000
C	14.369671000	2.241203000	12.208953000
C	15.885380000	9.299384000	0.913382000
C	13.494784000	7.397453000	10.549618000
C	15.204468000	15.749300000	-2.058057000
C	16.351071000	9.741207000	-0.381827000
H	16.853933000	9.107931000	-1.120082000
C	14.935942000	1.868843000	10.970156000
C	13.749919000	14.420454000	2.528771000
H	13.661828000	15.233284000	1.800560000
C	15.671849000	3.911054000	5.624323000
H	16.027870000	3.253803000	4.824940000
O	13.558062000	-2.802364000	16.199561000
O	18.440608000	2.719117000	6.297658000
C	11.461762000	15.616880000	8.210934000
H	10.948328000	16.359737000	8.830481000
C	16.059963000	11.079208000	-0.474888000
H	16.282649000	11.756540000	-1.305730000

C	12.768194000	15.914171000	7.769874000
C	13.307345000	14.405886000	3.827986000
H	12.791490000	15.202941000	4.373364000
C	17.711378000	1.612981000	5.949159000
C	13.161062000	6.988581000	11.855482000
C	13.293736000	5.658954000	12.313543000
C	13.269207000	8.337652000	14.008336000
C	16.002176000	0.610507000	9.415638000
H	16.542850000	-0.172110000	8.872860000
C	14.276518000	-0.711516000	15.365034000
C	11.368163000	8.606470000	12.491955000
H	10.885250000	8.321444000	11.547944000
C	13.189432000	3.886861000	13.719060000
H	12.996624000	3.210526000	14.558160000
C	15.264571000	16.753958000	-3.162399000
C	9.378605000	14.090961000	8.430178000
C	15.585916000	0.598166000	10.724488000
H	15.726415000	-0.197278000	11.463650000
C	12.853212000	5.213427000	13.616383000
H	12.336382000	5.836477000	14.353636000
C	15.791738000	0.126094000	6.224193000
H	14.837844000	-0.086515000	6.730024000
O	14.691070000	16.636127000	-4.236101000
C	15.844721000	6.621899000	-0.788784000
C	10.753994000	9.512249000	13.366170000
C	11.412122000	9.776249000	14.586872000
H	10.933502000	10.469081000	15.290589000
C	12.653193000	9.220360000	14.944399000
C	14.279520000	-1.696574000	16.488496000
C	18.190883000	0.808497000	4.879263000
C	14.348213000	13.611278000	-1.261330000
H	13.737498000	12.711254000	-1.425552000

C	17.748000000	6.315940000	0.713285000
H	18.261026000	6.572821000	1.653619000
C	9.422034000	10.212237000	13.043851000
C	18.339471000	5.408634000	-0.176258000
C	14.437116000	14.584305000	-2.261099000
H	13.907650000	14.457599000	-3.216656000
C	14.990965000	0.491815000	15.535091000
H	15.527942000	0.661137000	16.479793000
C	15.881266000	15.925502000	-0.833262000
H	16.486965000	16.827321000	-0.669321000
C	15.018840000	1.449103000	14.516862000
H	15.588737000	2.379697000	14.655324000
C	13.446882000	17.248629000	8.148026000
C	17.673555000	5.154095000	-1.391398000
H	18.140809000	4.458637000	-2.100198000
C	15.786361000	14.953878000	0.169098000
H	16.325055000	15.094614000	1.117830000
C	16.435266000	5.731147000	-1.732675000
C	15.732756000	14.234647000	6.727256000
H	15.813690000	13.541347000	5.864167000
H	15.574922000	13.644280000	7.653908000
H	16.687496000	14.790136000	6.815914000
C	17.450218000	-0.346632000	4.553843000
H	17.822052000	-0.981754000	3.742695000
C	13.625906000	0.013468000	13.132772000
H	13.080934000	-0.170561000	12.195110000
C	16.270686000	-0.732041000	5.219566000
C	13.591859000	-0.942656000	14.153810000
H	13.027760000	-1.875192000	14.015291000
C	13.322013000	9.567079000	16.291995000
C	13.814883000	18.029689000	6.865022000
H	14.509082000	17.455465000	6.224300000

H	14.299916000	18.992945000	7.128869000
H	12.906308000	18.262278000	6.271361000
C	13.570238000	7.140298000	-0.157075000
H	12.626214000	7.143979000	-0.737579000
H	13.565517000	8.022302000	0.517014000
H	13.610542000	6.215422000	0.455406000
C	16.143136000	18.835670000	-3.848715000
H	16.590577000	18.426474000	-4.776938000
H	16.798822000	19.617496000	-3.425704000
H	15.149877000	19.264406000	-4.091684000
C	19.689726000	4.780362000	0.144910000
H	19.825196000	4.866349000	1.245274000
C	19.462951000	1.189559000	4.091929000
C	13.507174000	-3.802200000	17.227644000
H	12.884103000	-4.622344000	16.828636000
H	13.052378000	-3.396117000	18.153668000
H	14.522558000	-4.178630000	17.465650000
C	15.555122000	7.856001000	13.390970000
H	15.575578000	6.970687000	12.721604000
H	16.496389000	7.870373000	13.975615000
H	15.502920000	8.777065000	12.773601000
C	14.714575000	16.976936000	8.991313000
H	14.462044000	16.418471000	9.916360000
H	15.182915000	17.936599000	9.295109000
H	15.468980000	16.394066000	8.432252000
C	12.518767000	18.146607000	8.987552000
H	11.586455000	18.411037000	8.448245000
H	13.042862000	19.095280000	9.222026000
H	12.238663000	17.679646000	9.953771000
C	15.746182000	5.399195000	-3.073904000
C	20.694128000	1.162085000	5.026847000
H	20.594549000	1.880425000	5.861211000

H	21.611360000	1.423193000	4.458347000
H	20.841347000	0.149950000	5.457268000
C	18.693405000	2.988812000	7.677163000
H	19.660325000	3.528326000	7.723380000
H	18.777353000	2.052006000	8.266598000
H	17.912929000	3.635437000	8.130057000
C	12.449865000	10.514577000	17.136843000
H	12.280224000	11.490749000	16.638481000
H	12.961647000	10.722278000	18.098386000
H	11.461344000	10.073907000	17.378965000
C	19.290947000	2.599323000	3.476105000
H	18.418414000	2.627210000	2.791079000
H	20.192025000	2.867752000	2.886959000
H	19.142173000	3.370921000	4.253249000
C	20.827425000	5.572769000	-0.523440000
H	20.737442000	5.538618000	-1.630078000
H	21.818609000	5.153423000	-0.251306000
H	20.812642000	6.638859000	-0.217835000
C	8.367438000	9.823222000	14.103316000
H	8.183904000	8.728943000	14.102350000
H	7.402055000	10.330887000	13.896663000
H	8.683380000	10.112154000	15.126208000
C	8.816728000	15.209351000	9.323466000
H	8.720652000	16.170838000	8.778906000
H	9.447321000	15.382936000	10.219356000
H	7.804633000	14.930769000	9.681219000
C	9.640947000	11.741835000	13.075811000
H	9.955711000	12.093504000	14.079137000
H	8.704154000	12.276357000	12.813963000
H	10.422703000	12.049576000	12.351521000
C	19.734523000	0.211929000	2.933327000
H	19.919451000	-0.823270000	3.285682000

H	20.641245000	0.538773000	2.384795000
H	18.900909000	0.184975000	2.202110000
C	8.889596000	9.821092000	11.655215000
H	9.598564000	10.090110000	10.846080000
H	7.937678000	10.352484000	11.452909000
H	8.682687000	8.734046000	11.580936000
C	8.428144000	13.896316000	7.228003000
H	8.365815000	14.818446000	6.613983000
H	7.403744000	13.649717000	7.577617000
H	8.762297000	13.071597000	6.566537000
C	19.766664000	3.291883000	-0.221507000
H	18.937858000	2.711022000	0.231173000
H	20.721312000	2.852032000	0.133238000
H	19.728873000	3.135501000	-1.320047000
C	9.433958000	12.786771000	9.257682000
H	9.794718000	11.928449000	8.655486000
H	8.426251000	12.525370000	9.641866000
H	10.111477000	12.896719000	10.128623000
C	13.538688000	8.275504000	17.114531000
H	12.570106000	7.777474000	17.328430000
H	14.014795000	8.517102000	18.087991000
H	14.186621000	7.556413000	16.580395000
C	14.383534000	4.715282000	-2.815132000
H	14.513970000	3.781771000	-2.229535000
H	13.905012000	4.443389000	-3.779309000
H	13.686056000	5.372511000	-2.264891000
C	16.591282000	4.435064000	-3.927523000
H	17.585514000	4.856547000	-4.179811000
H	16.065622000	4.237015000	-4.883612000
H	16.747323000	3.456100000	-3.430218000
C	15.547330000	6.695354000	-3.893768000
H	14.918835000	7.426732000	-3.353203000

H	15.057454000	6.463799000	-4.862857000
H	16.523458000	7.174343000	-4.116668000
C	14.673274000	10.277438000	16.044775000
H	15.387550000	9.634049000	15.499556000
H	15.138601000	10.557933000	17.012939000
H	14.529250000	11.208661000	15.458727000
C	15.493954000	-2.014899000	4.872395000
C	15.404871000	-2.903811000	6.133206000
H	16.414751000	-3.193305000	6.490547000
H	14.840464000	-3.834575000	5.915198000
H	14.887309000	-2.387189000	6.966833000
C	16.175296000	-2.820374000	3.753658000
H	16.240373000	-2.246781000	2.806559000
H	15.590529000	-3.738724000	3.542622000
H	17.200215000	-3.138479000	4.034059000
C	14.068723000	-1.639746000	4.408370000
H	13.507999000	-1.092913000	5.193139000
H	13.487907000	-2.552255000	4.158661000
H	14.098759000	-0.997159000	3.504253000
Cu	14.716571000	10.365101000	3.535994000
Cu	14.453052000	4.603858000	9.686939000
